# Conserved RNA structures in the intergenic regions of ambisense viruses

**DOI:** 10.1038/s41598-017-16875-4

**Published:** 2017-11-30

**Authors:** Michael Kiening, Friedemann Weber, Dmitrij Frishman

**Affiliations:** 1 Department of Bioinformatics, Wissenschaftszentrum Weihenstephan, Technische Universität München, Maximus-von-Imhof-Forum 3, D-85354 Freising, Germany; 2 Institute for Virology, FB10-Veterinary Medicine, Justus-Liebig University, D-35392 Giessen, Germany; 3 St Petersburg State Polytechnic University, St Petersburg, 195251 Russia

## Abstract

Ambisense viruses are negative-sense single-stranded RNA viruses that use a unique expression strategy. Their genome contains at least one ambisense RNA segment that carries two oppositely oriented reading frames separated by an intergenic region. It is believed that a structural RNA element within the intergenic region is involved in transcription termination. However, a general overview over the structural repertoire of ambisense intergenic regions is currently lacking. In this study we investigated the structural potential of the intergenic regions of all known ambisense viruses and compared their structural repertoire by structure-guided clustering. Intergenic regions of most ambisense viruses possess a high potential to build stable secondary structures and many viruses share common structural motifs in the intergenic regions of their ambisense segments. We demonstrate that (i) within the phylogenetic virus groups sets of conserved functional structures are present, but that (ii) between the groups conservation is low to non-existent. These results reflect a high degree of freedom to regulate ambisense transcription termination and also imply that the genetic strategy of having an ambisense RNA genome has evolved several times independently.

## Introduction

Ambisense viruses comprise a subsection of the segmented negative-sense single-stranded RNA viruses. In contrast to the genome of the purely negative-sense RNA viruses, the ambisense genome contains at least one segment with an additional positive-sense reading frame^1^. The two parts are oppositely oriented and separated by a noncoding intergenic region (IGR), where transcription termination takes place^2^. The gene of the negative-sense reading frame is directly transcribed from the viral RNA (vRNA) that is delivered by the infecting particles. To express the gene of the positive-sense reading frame, however, the genome first has to be faithfully copied by the viral RNA-dependent RNA polymerase into the viral complementary RNA (vcRNA) which, in turn, serves as template for transcription. Ambisense viruses are found within the entire family *Arenaviridae*, within the genera Phlebovirus and Tenuivirus of the family *Phenuiviridae* (order Bunyavirales), and in the family *Tospoviridae* (order Bunyavirales). Arenaviruses and Phleboviruses infect animals and humans, while Tospoviruses and Tenuiviruses infect plants.

It has been shown for some of the ambisense viruses, e.g. Arenavirus or Phleboviruses that transcription termination takes place within the IGR^3–8^ and it is suspected that a secondary structure element is involved in the termination process. Within the S segment of Arenaviruses Lassa virus and Mopeia virus this element is predicted to be a single- or a two-hairpin structure, respectively^9^. Within the IGRs of viruses belonging to the family Tospoviridae, stable tetraloop structures were identified^10^ and suggested to act in transcription termination^11^. Phleboviruses are thought to not contain any stable secondary structures in the IGR^7^.

So far, a general overview of the structural repertoire of ambisense IGRs is lacking. To fill this knowledge gap, we investigated the presence of conserved structural elements within the IGR of all known ambisense viruses. We demonstrate that the IGRs of most ambisense viruses have a high potential to build stable secondary structures, many of which are conserved within the phylogenetic groups, but not between them. Our findings imply that such structures are functional and that the ambisense coding strategy may have arisen several times independently during the evolution of segmented negative-strand RNA viruses.

## Material and Methods

### Data set of ambisense RNA segments

GenBank^12^ files containing genomic RNA sequences of the four known ambisense virus groups – Arenavirus (AV), Phlebovirus (PV), Tospovirus (TV), and Tenuivirus (TEV) - were collected using the NCBI taxonomy browser^13^. The sequences were filtered for segments fulfilling the typical criteria for the ambisense transcription strategy: two coding regions (CDSs) on opposite strands separated by an intervening noncoding IGR^1^. Subsequently, redundancy reduction was performed such that no two genomic segments shared 100% sequence identity in their IGRs. Sequences containing ambiguity codes corresponding to incompletely specified bases were excluded from consideration. In our final data set each virus segment was represented by its sequences in both 5′–3′ and 3′-5′ orientation, which are referred to as viral (v) and viral complementary (vc), respectively (Table 1).

**Table 1:** Ambisense virus data sets.

Virus	Segment	Strand	^a^Data set name	Number of sequences	^b^Average GC-content	^b^Average length [bp]
Arenavirus	S	v	AV-S-v (-IGR)	97	0.44 (0.69)	3386 (78)
	S	vc	AV-S-vc (-IGR)			
	L	v	AV-L-v (-IGR)	61	0.40 (0.75)	7179 (124)
	L	vc	AV-L-vc (-IGR)			
Tospovirus	S	v	TV-S-v (-IGR)	70	0.34 (0.22)	3045 (681)
	S	vc	TV-S-vc (-IGR)			
	M	v	TV-M-v (-IGR)	77	0.35 (0.22)	4809 (325)
	M	vc	TV-M-vc (-IGR)			
Phlebovirus	S	v	PV-S-v (-IGR)	168	0.46 (0.51)	1769 (120)
	S	vc	PV-S-vc (-IGR)			
Tenuivirus	2	v	TEV-2-v (-IGR)	35	0.39 (0.36)	3555 (314)
	2	vc	TEV-2-vc (-IGR)			
	3	v	TEV-3-v (-IGR)	53	0.38 (0.26)	2511 (754)
	3	vc	TEV-3-vc (-IGR)			
	4	v	TEV-4-v (-IGR)	53	0.39 (0.35)	2218 (665)
	4	vc	TEV-4-vc (-IGR)			

^a^Data set names are composed of three abbreviations: virus name-segment name–strand. If only the intergenic regions are used for a specific analysis, -IGR is added to the data set name. ^b^Values in parantheses refer to IRs only.

### Evaluating the structural potential of RNA sequences

**Figure 1:**
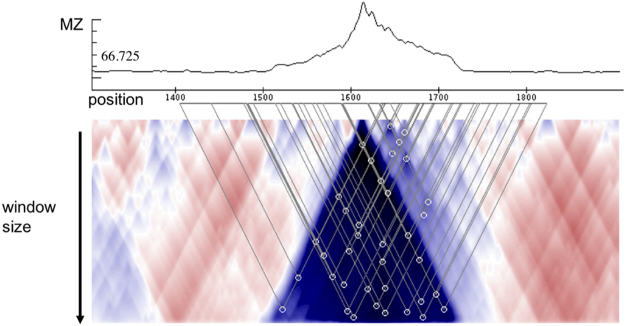
Fragment of the *RNAsurface* heatmap and the MZ plot for the Arenavirus segment S (GeneBank ID: AB261991), including the IR (positions 1579–1642). The line plot in the upper half of the picture shows the MZ values (y-axis) for each sequence position (x-axis). The heatmap below represents the surface of the structural potential, where the triangles represent locally optimal segments. Each point on the surface corresponds to a RNA subsequence. Colors represent the significance of the secondary structure predicted for the subsequence at each point. Blue corresponds to highly structured regions while red corresponds to unstructured regions. Circles in the heatmap denote local optimal segments.

To evaluate the structural potential within the IGRs and CDSs of ambisense viruses we employed the *RNAsurface* algorithm^14^. *RNAsurface* converts the values of minimal free energy (MFE) for a given RNA sequence into a z-score calculated as:

1 $${\rm{z}}=\frac{{\rm{E}}-{\rm{\mu }}}{{\rm{\sigma }}},$$

where E denotes the MFE while µ and *σ* are the average and standard deviation of the energy distribution of random sequences with comparable nucleotide composition and length. *RNAsurface* uses z-score evaluation to reconstruct the structural potential surface of an RNA sequence, which can be visualized by a two dimensional heat map (see Fig. 1). The x-axis corresponds to sequence positions while the y-axis corresponds to segment length. Each point and its color on the heat map represent a substring of the sequence and its structural potential. z-scores for all possible substrings of length between a certain minimal and maximal window size (W_min_, W_max_) are calculated. Locally optimal segments are visualized as peaks within the structural potential surface. A segment is regarded as locally optimal if small changes of its boundaries lead to worse z-scores. We used the default values of W_min_ and W_max_ of 30 and 200 nucleotides, respectively.

The structural potential surface defined in this fashion can be reduced to a one-dimensional curve reflecting the structuredness of a sequence segment. For each individual sequence position i the maximum squared z-score (MZ) among all negative z-scores of sequences of length between W_min_ and W_max_ covering this position is computed.

*RNAsurface* was applied to each strand of CDS and IGR RNA sequences separately. To make the resulting MZ curves comparable between sequences of different length n, sequence positions p were converted to relative positions p_rel_ on the percentage scale, with the leftmost and rightmost positions of each sequence corresponding to 0% and 100% for CDS1, 100% and 200% for the IGR, and 200% and 300% for CDS2 respectively, according to the following equations:

2 $$\begin{array}{rcl}{\rm{p}} < {{\rm{IR}}}_{{\rm{start}}}\to {{\rm{p}}}_{{\rm{rel}}} & = & \frac{{\rm{p}}}{{{\rm{IR}}}_{{\rm{start}}}-1},\\ \quad \,\,{\rm{p}}\in {\rm{IR}}\to {{\rm{p}}}_{{\rm{rel}}} & = & (\frac{{\rm{p}}-{{\rm{IR}}}_{{\rm{start}}}}{{{\rm{IR}}}_{{\rm{start}}}-{{\rm{IR}}}_{{\rm{end}}}}\ast 100)+100,\\ {\rm{p}} > {{\rm{IR}}}_{{\rm{end}}}\to {{\rm{p}}}_{{\rm{rel}}} & = & (\frac{{\rm{p}}-{{\rm{IR}}}_{{\rm{end}}}}{{\rm{n}}-{{\rm{IR}}}_{{\rm{end}}}}\ast 100)+200,\end{array}$$

with ${{\rm{IR}}}_{{\rm{start}}}$ and ${{\rm{IR}}}_{{\rm{end}}}$ being the first and last residue within the IGR respectively.

### Clustering and identification of evolutionary conserved RNA structures in the intergenic regions of ambisense segments

In addition to the analysis of the structural potential described above we delineated conserved structural motifs in all 16 IGR of ambisense viruses listed in Table 1 by performing motif clustering using *RNAclust* ^15^. Applying this technique to CDS regions would be computationally prohibitive because they are too long to build structure guided alignments in reasonable time. In a first step *RNAclust* employs *RNAfold* from the Vienna package^16^ to calculate for each input sequence the base pairing matrix, which contains the probability for each base to be paired with each other base of the sequence. These matrices are then used to calculate pairwise alignments between all input sequences using *LocARNA* ^17^. Subsequently, a hierarchical tree, based on the pairwise alignment scores is derived, using the WPGMA (weighted pair group method with averaging)^18^ method. Each node in the tree corresponds to a possible cluster of sequences sharing a structural motif. For each node a multiple sequence alignment (MSA) and a consensus secondary structure are computed using *mLocARNA* and *RNAalifold* ^19^, respectively. *(m)LocARNA* performs a variation of the Sankoff Fold and Align algorithm and thus produces structure guided (multiple) sequence alignments^17,18^.

To obtain the final motif clusters on the tree we employed *RNAsoup*, a script implementing an adapted version of the Duda and Hart^20^ rule to find the optimal motif clusters. Briefly, the null hypothesis is that the sequences i to n belonging to an internal node C form one cluster and should not be further split into separate clusters corresponding to the child nodes of C, C1 and C2. The squared error for this hypothesis (Je(1)) is calculated as a sum of differences between the MFE of each individual sequence (Ei) and the consensus of all sequences belonging to C (Econs)^21^:

3 $$Je(1)=\sum _{i=1}^{n}{({E}_{i}-{E}_{cons})}^{2}$$

The squared error for the opposite hypothesis, namely that the cluster C should be split into separate clusters C1 and C2, is calculated as:

4 $$Je(2)=\sum _{j=1}^{2}\sum _{i=1}^{{n}_{j}}{({E}_{i}-{E}_{cons})}^{2}$$

The null hypothesis is rejected if:

5 $$\frac{Je(1)}{Je(2)} < 1-\frac{2}{\pi }k\sqrt{\frac{2-\frac{16}{{\pi }^{2}}}{n}}$$

where k is a user-defined parameter, with larger k values resulting in larger clusters and vice versa. The procedure results in an hierarchical tree containing all input sequences, which are then divided into clusters. Since altering the levels of k leads to a change in the cluster size, each value of k represents a possible clustering. Based on the analysis of Rfam sequence families^15^ it was previously shown that k values between 0.8 and 1.2 result in the best structural consistency of RNA sequences within each family. We discuss the optimal choice of the k value for our study in the Results section. For the visualization of the structures FORNA^22^ was used.

### Potential functionality of structures

The clusters predicted by the *RNAclust* pipeline were further checked for functionality using *RNAz* ^23^. For each set of aligned RNA sequences secondary structure prediction was affected by *RNAz*, which implements the ‘Align, then Fold’ strategy. The *RNAz* method uses the *RNAfold* algorithm from the Vienna package to calculate secondary structures and the corresponding MFE for each individual RNA sequence in the alignment. In addition, for each aligned sequence set *RNAz* calculates a consensus secondary structure and its MFE using the *RNAalifold* algorithm from the Vienna package. Subsequently *RNAz* calculates three measures of structure conservation: i) the MFE z-score for each individual sequence, ii) the mean MFE z-score amongst all sequences, and iii) the structure conservation index (SCI) of the entire alignment. The SCI is calculated as the average MFE value of the sequences contained in the input MSA, divided by the MFE value of the consensus structure. Similar to the *RNAsurface* described above, an *RNAz* z-score describes the number of standard deviations by which the MFE of a given sequence deviates from the MFEs of a set of randomized sequences with the same length and base composition. However, since MFE calculations for a large set of randomized sequences are computationally prohibitive, *RNAz* predicts z-score values for each sequence by a support vector regression that estimates the mean and standard deviation of random MFEs dependent on the nucleotide composition of the given sequence. Negative and positive z-scores indicate structures that are more and less stable, respectively, than would be expected by chance^23^. The mean z-score is calculated as the sum of all individual z-scores divided by the number of sequences. *RNAz* assumes that conserved and thermodynamically stable structures are functional, in which case it outputs ‘RNA’, otherwise it outputs ‘OTHER’. For this purpose a p-value, called class probability, is calculated. p-values greater or smaller than 0.5 trigger the prediction of the ‘RNA’ or ‘OTHER’ class, respectively. The default classifier of *RNAz* is trained using MSAs that are created without any structure information. Predicting conserved structures based on alignments derived from *LocARNA* would result in an over-prediction of functional RNAs, as *LocARNA* alignments are already guided by structures. To avoid such over-prediction the option ‘-l′ for *LocARNA* type alignments was used, which enables a different training model in *RNAz* that is optimized on structure guided alignments.

### Deducing recurring structural motifs using shape abstraction

The clustering procedure described above joins together sequences that display global structural similarity, as judged by the SCI calculated over the entire sequence length. In order to find local structural motifs shared by ambisense viruses we converted the consensus structures of each cluster into the abstract shape notation^24^. Shapes are defined at five levels of abstraction – from the most realistic to the most abstract ones:

- *Level 1:Nesting pattern for all loop types and all unpaired regions*.
- *Level 2:Nesting pattern for all loop types and unpaired regions in external loop and multiloop*.
- *Level 3:Nesting pattern for all loop types, but no unpaired regions*.
- *Level 4:Helix nesting pattern and unpaired regions in external loop and multiloop*.
- *Level 5:Helix nesting pattern and no unpaired regions*.

In order to infer the overall structural similarity between RNA molecules we used the most abstract level 5, which strongly compresses structural diversity. In addition level 3, which provides the best trade-off between accuracy and abstraction, was used to assess specific differences between the consensus structures.

To convert the dot-bracket notation of *RNAalifold* into shape notation the tool RNAshape^25^ was used. Only shapes of the same type can be compared with each other. For each data set we locally aligned the consensus shapes of all clusters against each other using the dynamic programming tool water from the EMBOSS toolkit^26^. As water accepts only nucleotide sequences as input, we replaced the shape notation with nucleotides (‘[‘=’A’, ‘]’=’T’, ‘_’=G), and used a simple scoring scheme (match +1, mismatch 0, gap −1). The nucleotide alignments were then translated back into the shape notation.

### Investigation of the relationship between sequence and structure conservation in the intergenic and coding regions

To investigate sequence-structure relationships in ambisense virus segments we calculated two measures of evolutionary conservation using *RNAz*: SCI and mean pairwise identity (mPID). In order to find out whether the relationship between mPID and SCI is different if pure sequence alignments, rather than structure guided alignments, are employed we also conducted the same analysis using the multiple sequence alignment method *clustalOmega* ^27^. To derive the SCI and mPID values for the three regions (CDS1, CDS2, IGR) in each cluster we first multiply aligned sequences and then split the MSAs into overlapping windows using the helper perl script rnaz*Window.pl*, which is part of the *RNAz* package. As the full-length CDS are too long to produce structure guided alignments in reasonable time, we realigned the alignment windows initially produced with *clustalOmega* using *mLocARNA*. In a last step the alignment windows generated by both methods were passed to *RNAz* and correlation coefficients between mPID and SCI for each region were calculated separately.

### Data Availability

The datasets generated during and/or analysed during the current study are available from the corresponding author on reasonable request.

## Results and Discussion

### Highest structural potential within IGR in most sequences

**Figure 2:**
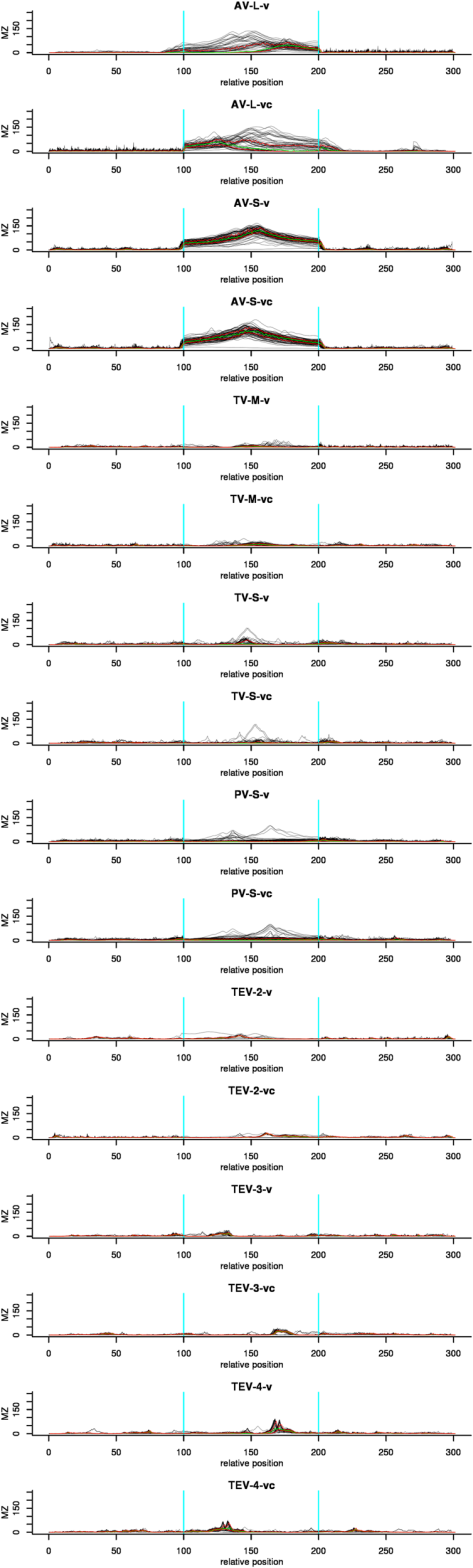
Structural potential plots for all data sets listed in Table 1. The x-axis corresponds to the relative sequence position, with the ranges 0–100%, 100–200%, and 200–300% corresponding to CDS1, IR, and CDS2, respectively. Black lines indicate maximum squared z-score values (MZ) of individual sequences derived from *RNAsurface*. Green and red lines correspond to the median and 25%/75% quartiles, respectively. Vertical blue lines highlight the borders of the IR. High MZ values indicate potentially structured regions.

**Figure 3:**
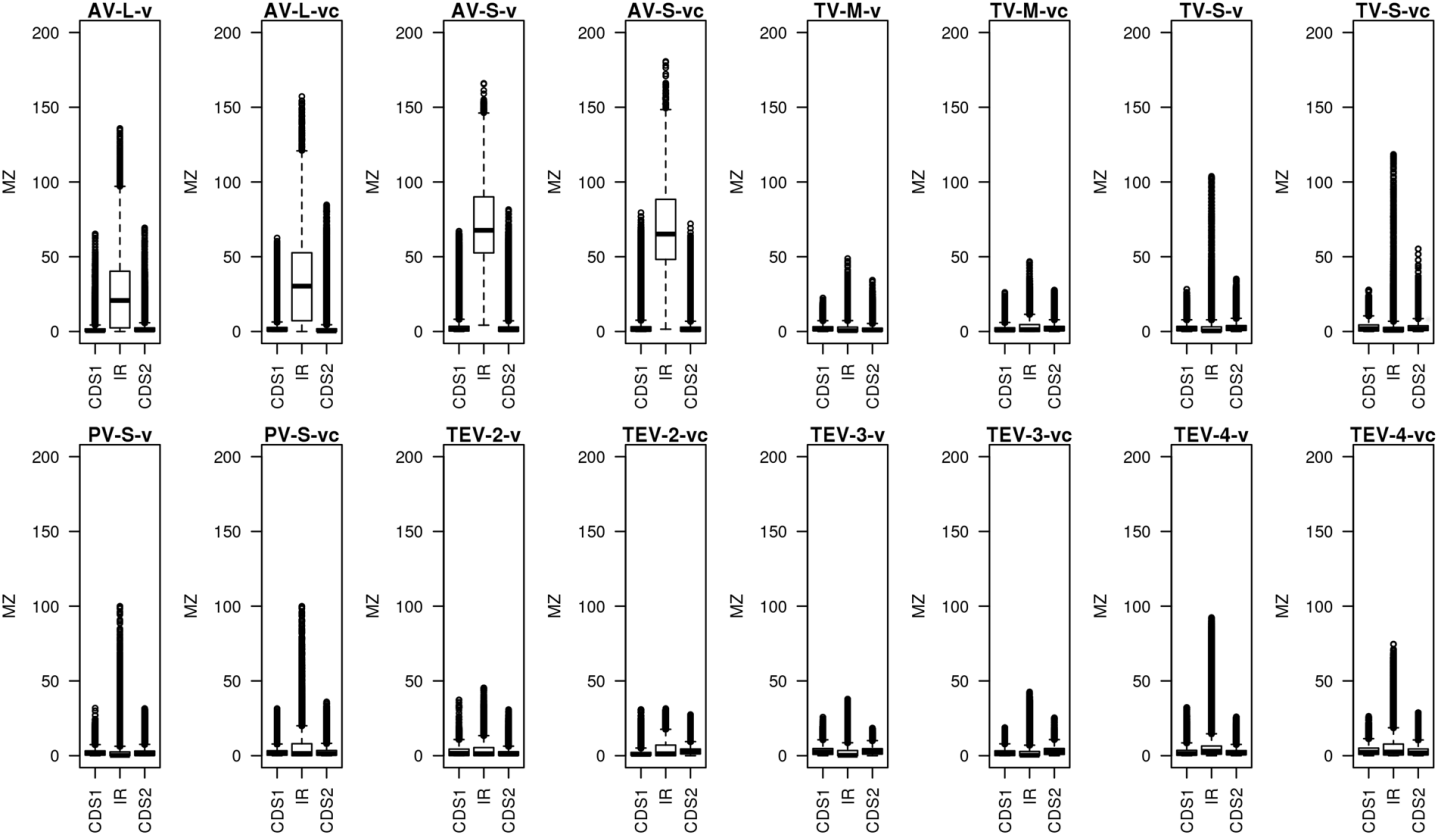
Boxplots showing the distribution of MZ values in each data set for CDS1, IR, and CDS2. MZ values reflects the potential of a sequence to form stable secondary structures.

For each of the data sets listed in Table 1 we compared the structural potential of the IGRs to that of the CDSs by calculating MZ values for each sequence position using *RNAsurface* (Figs 2 and 3). Sequence positions were converted to a percentage scale for each part of the sequences (CDS1, IGR, CDS2), as explained in *Methods*. The largest contrast in structure potential between CDSs and IGR was identified in the Arenavirus data sets. The median MZ values within the IGRs are between 20 for AV-L-v and 67 for AV-S-v, while in the CDSs they range between 0.2 and 2. In TV and TEV the difference in structural potential between CDSs and IGR is less pronounced, although the highest MZ peaks are still located within the IGR (see boxplots in Fig. 3). Within the TV sequences and the sequences of TEV segment 4 the higher structuredness of the IGR is still quite apparent, while in TEV segments 2 and 3 the difference between CDS and IGR is very small. The CDSs of PV appear to contain potentially structured regions, while their IGRs can be subdivided into three groups: i) those with no structural potential within IGRs at all (24% and 43% for v and vc, respectively), ii) those containing local optimal segments, whose MZ peak is however lower than that of the CDSs (25% and 45% for v and vc, respectively), and iii) those containing the highest MZ peak in the entire sequence, including CDSs (51% and 12% for v and vc, respectively). Thus, our analysis shows that there is high structural potential within the IGSRs of most of the ambisense virus sequences.

### Structural Clustering

Using the *RNAclust* pipeline, hierarchical trees and multiple structure guided sequence alignments were calculated for each data set. The trees, and a table showing all sequences in their corresponding clusters can be found in the supplementary material (Figures S1, S2 and Tables S1A, S1B). The full clustering results including all alignments are available upon request. Sequence clusters potentially sharing common structural motifs were delineated from trees using a certain cutoff value of the parameter k (see *Methods*).

**Figure 4:**
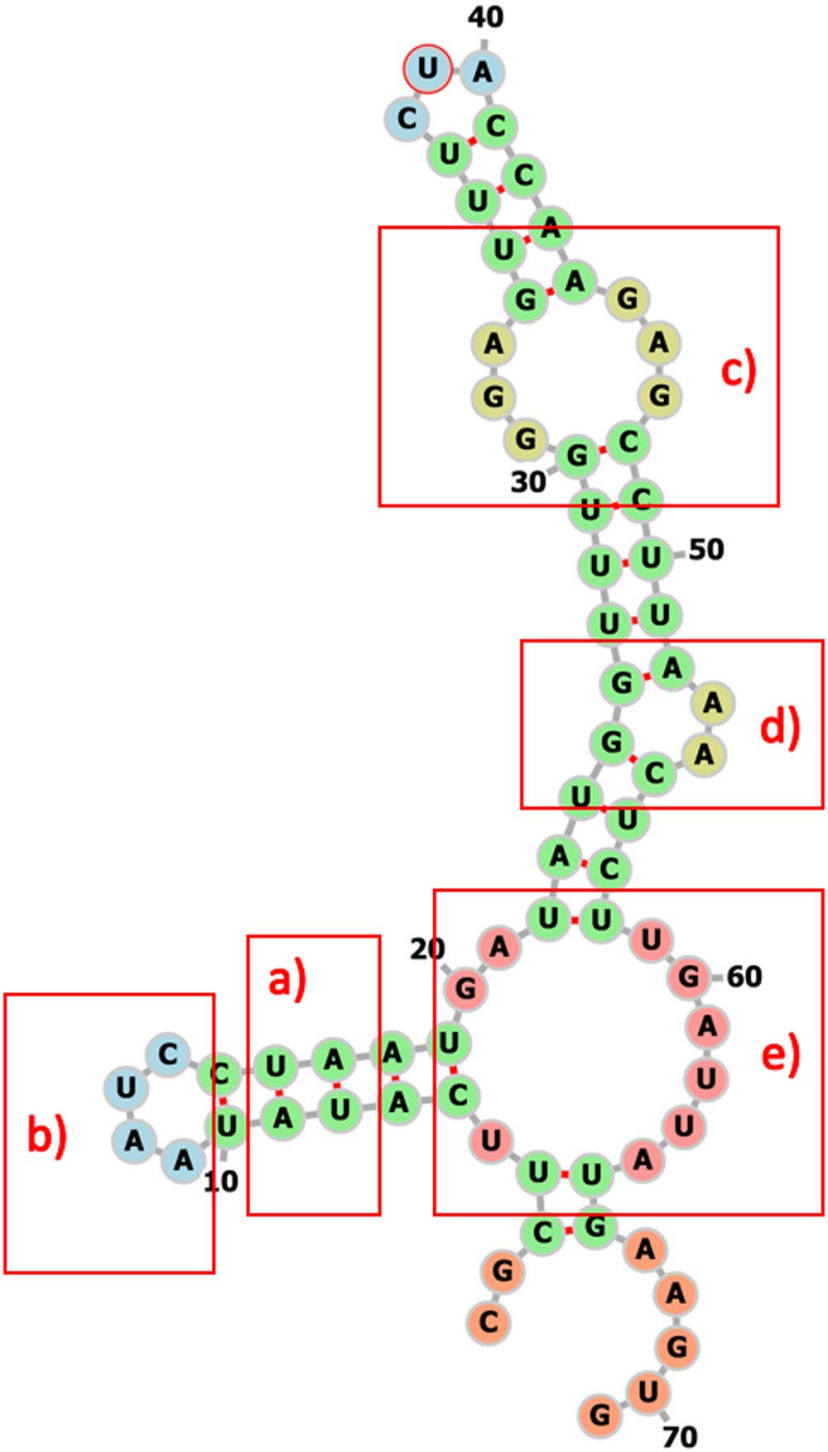
Example structure visualizing the structural features used in our analysis: (**a**) stem, (**b**) loop, (**c**) internal loop, (**d**) bulge, (**e**) multi loop.

The consensus structure of each cluster is described in terms of the structural features it contains (Table S2). We define the following five structural features (Fig. 4):

1. Stem: a series of consecutive base pairs that can include bulges and/or internal loops.
2. Loop: a series of unpaired residues followed by one base pair. Smaller loops are named according to the amount of unpaired residues in the loop, e.g. triloop (three unpaired residues), tetraloop (four unpaired residues) or pentaloop (five unpaired residues).
3. Internal loop: two or more unpaired residues on both sides of a stem.
4. Bulge: a series of unpaired residues on one side of a stem.
5. Multiloop (Mloop): a region where at least three stems come together. A stem can be followed by either unpaired residues or by another stem.

We identified a large number of structural motifs in the clustered IGRs of ambisense viruses (Tables S2 and S3). The clusters are characterized by the mPID of the underlying structure guided MSA, the SCI, the *RNAz* prediction of functionality (‘RNA’ or ‘OTHER’), and by structural features contained in the consensus structure, if available. We paid special attention to the hairpin loop motifs, which are believed to play a role in transcription termination of ambisense RNAs^1^. Using the recommended values of k (between 0.80 and 1.20) we were not able to reproduce previously reported structural features of TV sequences (e.g. y-shaped tetraloops^10^), which only became visible in our clusters at k = 0.6. Based on this limited benchmark we used the value of k = 0.6 in this study. A lower value of k results in more fine-grained clusters, which is acceptable as no wrong structures are accumulated in a cluster. The size of clusters and their number vary drastically between data sets (Figure S2).

### Conserved stem-loop structures in Arenaviruses

**Figure 5:**
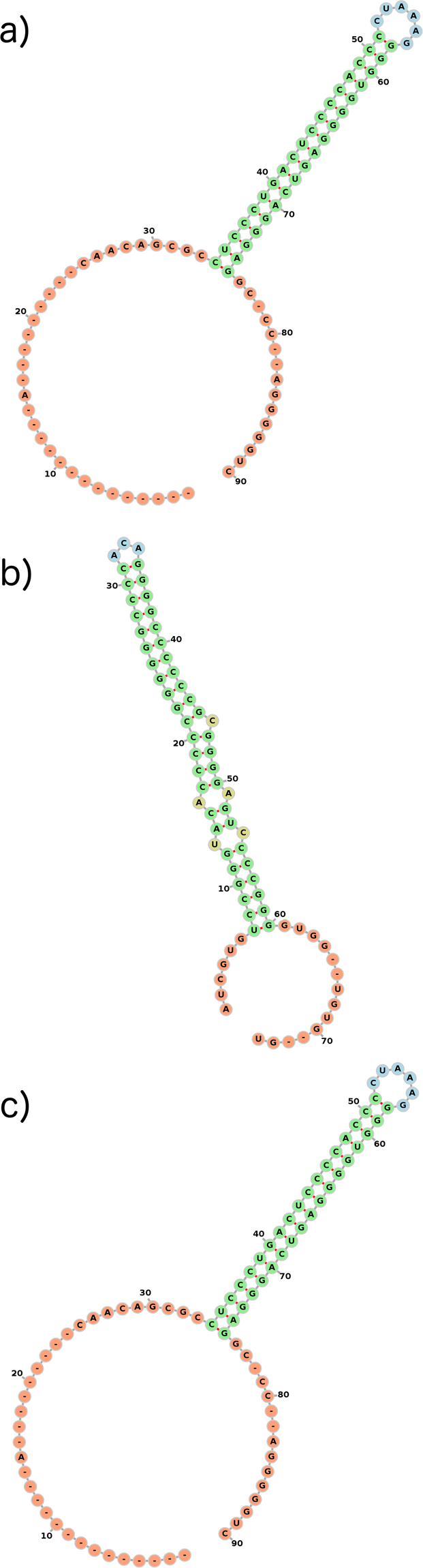
Consensus secondary structures, visualized with *FORNA*: (**a**) AV-S-v-IR cluster 3 (**b**) AV-L-v-IR cluster 55 (**c**) TV-M-v-IR cluster 11.

In general, clustering results echo those obtained by analysing the structural potential of IGRs. Arenavirus sequences show the strongest structural conservation among all data sets, as evidenced by the consistently high SCI values in the AV-S-v/vc-IGR data sets (Table S2). Almost all the clusters containing more than one sequence (either viral and viral complementary) are predicted by *RNAz* to contain functional RNAs (Table S2 and Figure S2). The only structural features present in the consensus structures of the S segment are stems and loops (Table S3). The stem size varies between 5 and 18 base pairs, and the loop size between 3 and 7 unpaired bases. The biggest cluster in the AV-S-v-IGR set (node ID 3 in the hierarchical tree) consists of 56 sequences. The only consensus stem-loop structure for this cluster, predicted with *RNAalifold*, contains the hexaloop sequence ‘CCUAAAGG’ (Fig. 5a), with the first and the last bases forming a base pair. The mPID of the alignment is 0.65 while the SCI is 0.94 (Table S2). The high SCI value indicates that the structures of the individual sequences within the cluster are energetically very close to the consensus structure, and thus are strongly conserved. The cluster contains 18 species (Tables S1A, B), of which four have been previously reported to contain the single stem-loop feature: Pichinde^28^, Lymphotic choriomeningitis virus (LCV)^29^, Lassa virus^30^ and Lujo virus^31^. Thus, our analysis proposes 14 further species to fall into this structural class. The smaller clusters have more than one stem-loop structure. Clusters 155, 138 and 180 consist of 13, 9 and 5 sequences, respectively, and have two stem-loops. This structural feature was already described for the Tacaribe virus^6^, which did not fit into any cluster in our analysis. All sequences but one, the Morogoro virus, that share the two stem-loop feature belong to the group of new world Arenaviruses. Cluster 119 consists of 10 sequences, all belonging to the species Junin virus, and has three stem-loops, two triloops and one pentaloop. The two triloops found in the Junin virus sequences are already described in the literature^32^, while the pentaloop has not been mentioned before. All Machupo virus sequences form one cluster (138) and also share two highly conserved triloops. All but one found consensus structures consist of loops with GC closing pairs and thus are very stable. The AV-S-vc-IGR set is subdivided into a larger number of smaller clusters, but shows a similar distribution of stem-loops, also with very high SCI values.

The IGR structures of the L segment of AV have received much less attention in the literature so far. The distinction between the New World and the Old World Arenaviruses becomes even more apparent within the L segment, as all clusters only contain sequences from one of these groups. In contrast to S segment, which only harbors stems and loops, some consensus structures of the L segment contain two further structural features, internal loops and Mloops (Table S3). The stem size varies between 3 and 27 base pairs, the loop size between 3 and more than 10 unpaired residues, and the internal loop size between 2 and 9 unpaired residues. The biggest cluster (node ID 55 with 10 sequences), containing only Old World Arenaviruses of the species Lassa virus, Mopeia virus, Mobala virus and Luna virus Arenaviruses also adopts the single stem-loop feature with an additional internal loop (Fig. 5b). The consensus structure of this cluster has quite a high SCI value of 0.74 and the underlying MSA has a mPID of 0.57 (Table S2). The representatives of the New World Arenaviruses, Machupo virus, Tacaribe virus, Sabia virus and Chapere virus share one conserved triloop, while all other members of this group have at least two stem-loops. The clusters comprising LCV (92, 97, 106) tend to contain more than one stem-loop, connected through an Mloop (Table S3, structures 10, 11). The stems sometimes also contain internal loops. Only one cluster containing LCV sequences (113) shows a different consensus with only one stem-loop.

As expected, Arenavirus sequences belonging to the same species tend to appear in the same cluster (this also applies to all other ambisense virus genera). Our analysis thus indicates that structures are more conserved within species than between them. It is also remarkable that all clusters in the AV-S-v/vc-IGR data set, except the cluster 180 in the v set and the cluster 69 in the vc set, as well as all clusters in the AV-L-v-IGR data set, and all clusters in the AV-L-vc-IGR data set, except clusters 8 and 16, are predicted to contain functional RNAs by *RNAz* (Tables S2 and S3). This finding is in line with the previous studies that show that a stable hairpin in the IGR of Arenaviruses is required for transcription termination^6^.

### Conserved motifs within Tospovirus segment M

The TV-M-v/vc-IGRs contain diverse structural features, with stem sizes varying between 4 and 44 base pairs and loops containing between 3 and 10 unpaired nucleotides (Table S3). All clusters of the v set contain at least one multiloop with 3 base pairs, internal loops between 2 and 11 nucleotides, as well as occasional bulges of size 1 to 3 nucleotides. The four tomato spotted wilt virus (TSWV) clusters (5, 31, 71, 90) share two strongly conserved y-shaped tetraloops: ‘UGAAAA’ and ‘CCGAAG’ (Table S3, structure 16). Clusters 71 and 90 additionally have a pentaloop (‘UGACAAG’) and a decaloop (‘UAAUCUGACUAA’) in common, while cluster 5 contains 2 additional triloops (‘CAAUG’, ‘UCAAA’) that are not conserved in other clusters. The presence of y-shaped tetraloops in the IGR of the TV-M segment was reported previously^10^. Cluster 110 also contains three tetraloops (‘AAAUAU’, ‘CUUAGG’, ‘UUUU-A’), of which two adopt the y-shape (Fig. 5c). Cluster 115 is the only cluster where no consensus structure could be found at all. The vc sequences show the same picture. All TSWV clusters (49, 56, 64, 84) share the tetraloop ‘UUUUCA’, (Table S3, structures 18, 20, 22) with the exception of the cluster 6, in which this tetraloop exhibits the sequence ‘UACAAA’. The y-shape is not always present in the vc sequences, where some of the structures tend to build a longer stem loop (Table S3, structure 22). The TSWV clusters also contain a number of partially conserved internal loops as well as triloops, tetraloops and several larger loops up to a septaloop.

The IGRs of the TV-M segment, with a mean length of 324 nucleotides, are quite long compared to the size of the local structural elements, that range in size between 40 and 80 nucleotides. For this reason the overall conservation signal in terms of the global SCI tends to be weak.

### Phleboviruses

Based on the structural elements contained in the IGR, PV sequences can be separated into several groups. The first group (clusters 6, 296, 299, 130, 135, 138, 146, 151, 156, 165, 200, and 282) contains only one stem-loop and in some cases internal loops (Table S3, structure 23). The stem size within this group varies between 3 and 15 base pairs (Table S3). The loops typically contain between 4 and 15 unpaired residues, although larger loops with more than 20 unpaired residues also exist. The second group (clusters 289, 319, 271, 227, 127, 230, and 214) possesses consensus structures containing several stem-loops, with the stem length between 3 and 40 base pairs, the loop size ranging between 3 and more than 20 unpaired nucleotides, the internal loops containing between 2 and over 20 unpaired nucleotides, and some bulges of size 1 or 2 (Table S3, structure 25). The third group (clusters 221, 235, 279, 286, 306, 309, and 332) is characterized by the presence of an additional multiloop with 3 to 6 base pairs (Table S3, structure 24). In all three groups we find clusters predicted to contain functional RNAs by *RNAz* (see Methods) as well as clusters predicted to not contain functional RNAs (Tables S2 and S3). Finally, the fourth group, comprising all other clusters, does not contain any structural elements at all. We were unable to find any published indication that transcription termination by phleboviral IGRs depends on a specific secondary structure element. However, for Rift Valley fever virus it was shown that the UTR of the L segment (which is not ambisense) forms a functional stem-loop structure that takes part in transcription termination^8^. Our analysis indicates that at least some Pheboviruses contain similar local structural elements in the IGR that could serve as transcription regulating elements.

### Tenuiviruses

Sequences from the TEV-2-v/vc-IGR data sets possess the lowest structural potential compared to all other data sets. The structural potential of a sequence is assessed based on the z-scores of its MFE structures and thus reflects the significance of these MFE structures^14^. Low structural potential in a set of sequences may imply that the MFE structures are random and thus unlikely to be evolutionarily conserved. Indeed, the *RNAclust* analysis revealed that although the IGRs are very similar at the sequence level, the structural conservation in terms of the SCI is weak for the v sequences and even lower for the vc sequences. The biggest cluster of the v set (node ID 4), making up 70% of the data set, has a mPID of 96% and a SCI of 78%. In the vc set, all sequences are clustered into one cluster (0) sharing a mPID of 93% and a SCI of 25%. All consensus structures of both sets are predicted to be non-functional by *RNAz*. We were thus unable to detect any structural conservation in the IGRs of these sequences.

Similarly, the sequences of the TEV-4-v/vc-IGR data sets were grouped into a single cluster and showed almost no structural conservation, although they share a mPID of 81% and 87% in the v and vc set respectively, and both alignments were classified to contain a functional RNA by *RNAz*.

### The relation between the sequence and structural similarity in the intergenic and coding regions of ambisense viruses

**Figure 6:**
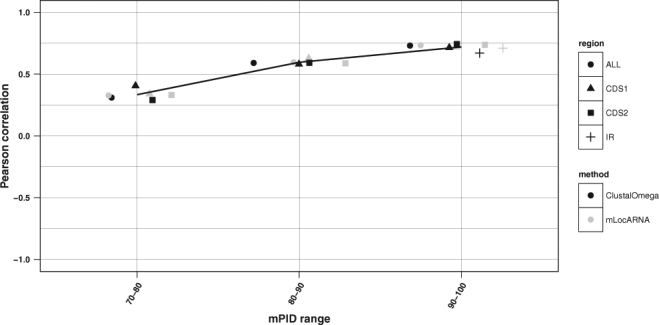
Correlation of mPID and SCI within different mPID threshold ranges. The shape of the data points refers to the three regions: triangles – CDS1, squares – CDS2, pluses – IR; circles represent all three regions taken together. Colours correspond to the alignment method used: grey – *mLocARNA*, black *ClustalOmega*. Only significant (confidence interval of 0.95, P-value < 0.05) correlations are shown. For better visibility of overlapping points a horizontal jitter function was applied. The black line shows the regression performed on all data points, and the grey shaded area the standard error. Correlations for the mPID values below 50% were not significant and are thus not shown.

**Figure 7:**
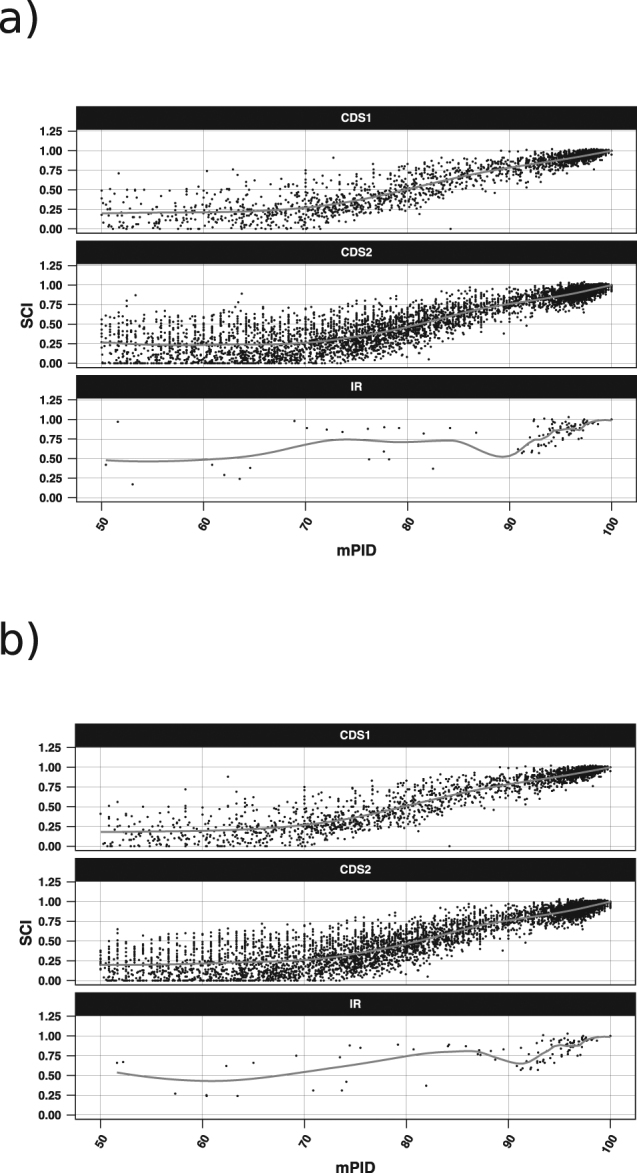
Comparison of sequence similarity in terms of the mPID on the x-axis and structure similarity in terms of the SCI on the y-axis, for the CDSs and IRs of all data sets for (**a**) Multiple sequence alignments generated with *ClustalOmega*. (**b**) Structure guided multiple sequence alignments generated with *mLocaRNA*. Each point corresponds to an alignment that covers a window of 120 nucleotides either of CDS1, CDS2 or the IR.

Previous research suggests that there is a certain relationship between mPID and structural conservation in RNA sequences. For non-coding RNAs, such as small nuclear RNAs, the correlation between sequence identity and structural similarity quickly increases as the sequence similarity approaches ~60% and saturates between 60–100%^33^, while in mRNAs this correlation only exists at sequence identity levels between 85% and 100%^34^. We investigated the relation between SCI and mPID in ambisense virus IGR and CDS based both on pure multiple sequence alignments computed by *ClustalOmega* and on structure guided alignments created with *mLocarna*. The SCI and mPID values were calculated for MSAs produced by both alignment algorithms for all nodes in the *RNAclust* hierarchical trees (at k = 0.6). Since the SCI and mPID values follow a normal distribution according to the Kolmogorov-Smirnov test^35^, we computed Pearson correlation coefficients between them and retained only significant correlations (p-value < 0.05; two sided t-test; Fig. 6). mPID values calculated both from sequence and structure-based alignments are strongly positively correlated with SCI, in the intergenic as well as in the coding regions (Figs 6 and 7; Table 2). The correlation is generally stronger for higher mPID values, which is in line with our previous results obtained for yeast mRNAs^34^.

**Table 2:** Correlation between mPID and SCI across all data sets.

Region	Pearson correlation coefficient between SCI and mPID	
	Sequence alignments (MAFFT)	Structure-guided alignments (mLocARNA)
CDS1	0.85	0.89
CDS2	0.86	0.89
IGR	0.34	0.89

### Inference of shape motifs

To find conserved local motifs in the IGRs of ambisense viruses, we converted the consensus secondary structures of RNAs in each cluster into shapes at the five levels of abstraction described in *Methods*. At each abstraction level shapes were locally aligned using the program *water* from the EMBOSS toolkit. To assess the differences between the consensus structures we chose layer 3 as the best tradeoff between the accuracy and abstraction, because it depicts all nesting patterns for all loop types, but no unpaired regions. To define the maximum motif the structures have in common, the most abstract level 5 was used. All shape consensus structures are listed in Table S4.

Within the AV-S-v/vc-IGR data sets the consensus structures already showed a high overall similarity between different clusters. Expectedly, the shape analysis led to qualitatively similar results: the consensus structures of the largest clusters, that make up 58% and 40% of the v and vc data set, respectively, contain a single stem-loop ([] in shape notation). 28% of the v set and 31% of the vc set share an additional stem-loop ([][]), and sometimes also internal loops (e.g. [][[]]). Only 10% of both data sets contain a third stem-loop ([][][]), occasionally with internal loops. Likewise, the results for the AV-L-v/vc-IGR data sets are as follows. 32% of the v and vc sets share one stem-loop (containing internal loops), 25% of both sets share two stem-loops (containing internal loops), three stem-loops are harbored by 15% of the data sets, and 10% of the v sequences as well as 16% of the vc sequences harbor four stem-loops.

The TV-M-v/vc-IGR data set showed a weak structural conservation based on the *RNAclust* analysis, but it is known from previous research that the sequences contain conserved structural elements^10^. Interestingly, this conservation is visible in the shape analysis, with the exception of cluster 115 (25% of the sequences) in the v set and cluster 122 (12% of the sequences) in the vs set, which did not produce any consensus structure. Clusters 90 and 71 from the v set show the exact same shape, consisting of four stem-loops, including the y-shaped loops already described in literature, and cluster 5 only differs from those by an additional internal loop. These three clusters make up ~43% of the sequences. Clusters 31 and 110 (30%) harbor only three stem-loops, but also share the y-shaped loops. The level 5 shape motif visible from the local alignments of all clusters of the v set, except 115 and 110, is [[[][]][]], while in the consensus structure of cluster 110 the third stem-loop is situated before the y-shaped loop: [[][[][]]]. Within the vc set, the structures are more diverse, but also share a conserved core. Cluster 64 (13%) consists of a single long stem-loop containing several internal loops and bulges, while cluster 111 (6%) harbors two loops. The majority of sequences share three stem-loops (31%). The remaining 30% of the sequences harbor four or more loop structures. The y-shaped motif is only visible in clusters 84 ([[][][[][]][]]) and 147 ([][[[][]][][]]), which account for 21% of the sequences; in both cases the y-shaped loops are part of a multiloop.

In line with the *RNAclust* analysis several groups become apparent within the Phlebovirus data sets (PV-S-v/vc-IGR):

- 11% of the v set and 21% of the vc set do not contain any structure.
- 58% of the v set as well as 29% of the vc sequences harbor one stem-loop ([]) and in some cases also internal loops ([[]]).
- Two stem-loops are harbored by 13% and 44% of the v and vc sequences, respectively.
- Within the viral sequences, 4% harbor three stem-loops.
- Finally, two groups of vc sequences, accounting for 10% and 5% of the data, respectively, are characterized by the presence of a multiloop and three ([[][][]]) to four ([[][][][]])stem-loops.

In contrast to what is currently known, the vast majority of the Phlebovirus sequences harbors at least one single stem-loop with a varying number of internal loops within the stems on the v strand and two stem-loops on the vc strand. Only a small proportion of sequences does not show any structural feature.

## Conclusions

Segmented negative-strand RNA viruses are in principle only capable of expressing one gene per segment^36^. To expand the coding capacity, they express additional genes either via polyprotein expression, insertion of ORFs with a shifted reading frame, or by the ambisense strategy^37,38^. The IGR is an inherent feature of the ambisense RNA viruses, pathogens of major medical and economical importance. Here, we present the first comprehensive comparison of the predicted IGR structures among and between the arenaviruses, tospoviruses, phleboviruses and tenuiviruses.

In order to obtain a general overview of the structural repertoire of the ambisense RNA segments, we explored their potential to build stable secondary structures by clustering the sequences globally based on structural similarity, predicting consensus structures for each cluster, and identifying local structural motifs using shape abstraction. The shape analysis showed that the consensus structures of the clusters can be further compressed due to their similar shapes, reducing the structural repertoire of the IGR to three to four recurring patterns within each data set. Arenaviruses showed the highest structural potential within their IGRs, and a very low degree of structuredness in the two CDSs. The structural potential of the Arenavirus IGRs is also clearly the strongest among all ambisense viruses. The most prominent feature of Arenavirus IGRs is the presence of at least one stem-loop structure, which is presumed to be a transcription termination signal. This structure has been previously discovered in four of the Arenavirus species, and our analysis also suggests that it is conserved in 14 further species. Approximately 60% of the sequences contain only one stem-loop structure (shape []). Further 30% of the IGRs contain two stem-loop structures (shape [][]) in 10 species, including the Tacaribe virus, where they have been previously described^6^. Finally, 10% of the IGRs, all belonging to the species Junin virus, contain three stem-loop structures (shape [][][]) – an arrangement that has not been mentioned in literature so far. According to previous research this species also belongs to the group, which contains two stem-loops^32^. Interestingly, only the sequences belonging to the “New world Arenaviruses” harbor more than one stem-loop. Almost all structures were classified as functional, which reinforces the assumption that they may play a role in transcription termination.

In contrast to Arenaviruses, Tospovirus sequences showed only a moderate structural potential in the IGR, although it is still higher than in the CDS regions. We found conserved y-shaped tetraloops and triloops in the IGRs of the M segment on the viral strand, with the y-shaped loops containing a varying amount of internal loops: i) [[[[[[][]]]]], ii) [[[][[]]]], and iii) [[[[[[][]]][]]. Sequences of the viral complementary strand tend to fold into a long single stem-loop ([]), while the y shape is only present in 21% of the vc sequences. 48% of the sequences harbor one to seven additional stem-loops. All consensus structures of the Tospovirus clusters, viral as well as viral complementary, were predicted to be functional.

The Phlebovirus IGRs have so far not been thought to contain any functionally relevant structural features^7^, which led us to expect a low structural potential in their IGRs. This is indeed true for most of the analysed Phlebovirus sequences. Nevertheless, a subset of Phlebovirus sequences, comprising approximately 76% and 57% of the vRNA and vcRNA data set respectively, showed a high potential to build stable secondary structures in the IGR. For 51% and 12% of the v and vc sequences, respectively, the potential is higher than the structural potential of the two CDS. *RNAclust* analysis confirmed that these structures are highly conserved within the corresponding clusters. Only 11% of the sequences did not produce any consensus structure. Shape analysis revealed that the most prominent feature on the vRNA strand is a single stem-loop with a varying amount of internal loops, and on the vcRNA strand two stem-loops with internal loops. The found structures are predicted to be functional. Tenuiviruses appear to be the only ambisense viruses that lack conserved structures in the IGRs, in spite of the high level of sequence similarity. Still the consensus structures of segment four were predicted to be functional.

The structure conservation index was used to identify conserved structures in the IGRs of ambisense viruses and to measure their reliability. As this measure is dependent on the sequence similarity of the underlying alignment^23^, we investigated the relation between sequence and structure conservation in terms of the mPID and the SCI respectively both for pure sequence and structure guided MSAs. The analysis showed that SCI and mPID are highly correlated both in the intergenic and in the coding regions, and the correlation is stronger for higher mPID values. Similarity of sequence-structure relationships between the coding and non-coding regions suggests that *RNAz* and other methods for predicting stable and conserved RNA secondary structures, which are usually trained on non-coding regions, can also be applied to coding sequences.

Genomic RNA of ambisense RNA viruses is largely encapsidated by N proteins^39^. In non-ambisense bunyaviruses, transcription is believed to be terminated by 3’ UTR structures formed in either the genome template or the nascent mRNA^40^. Similarly, whether the IGR-located transcription termination signal of ambisense bunyaviruses acts on the mRNA level, or whether it already forms on the nucleocapsids, is currently unknown.

To summarize, we detected certain levels of structural conservation (or the absence of any predicted structures) within the ambisense virus groups, but not between them. This may indicate that transcriptional termination *per se* could be achieved by many different types of IGRs. Other, group-specific, IGR functions may exist that constrain the structural freedom despite the fact that the IGR can be a target of antiviral host defences^41^.

In any case, our results indicate that the ambisense gene expression strategy has evolved several times independently as a means to alleviate the one-gene-per-segment restriction of the segmented negative-strand RNA viruses. These findings may improve our understanding of negative-strand RNA virus gene regulation and evolution and prompt further research into the structure-function relationship of the IGR.

## Electronic supplementary material

Supplementary information
