# Supplementary material for: Conserved RNA structures in the intergenic regions of ambisense viruses
Source: Sci Rep. 2017 Nov 30;7:16625. doi: 10.1038/s41598-017-16875-4 (PMC5709424; doi:10.1038/s41598-017-16875-4)
Supplement: Supplementary file 1 — Supplementary information [file 41598_2017_16875_MOESM1_ESM.doc]

# **Supplementary Material - Conserved RNA structures in the intergenic regions of ambisense viruses**

Michael Kiening^1^, Friedemann Weber^2^, Dmitrij Frishman^1,3,*^

^1^Department of Bioinformatics, Wissenschaftszentrum Weihenstephan, Technische Universität München, Maximus-von-Imhof-Forum 3, D-85354 Freising, Germany

^2^Institute for Virology, FB10-Veterinary Medicine, Justus-Liebig University, D-35392 Giessen, Germany

^3^St Petersburg State Polytechnic University, St Petersburg, 195251, Russia

^*^ To whom correspondence should be addressed at: Department of Bioinformatics, Wissenschaftszentrum Weihenstephan, Technische Universität München, Maximus-von-Imhof-Forum 3, D-85354 Freising, Germany. Tel: +498161712134; Fax: +498161712186; Email: [d.frishman@wzw.tum.de](mailto:d.frishman@wzw.tum.de)

| a) | 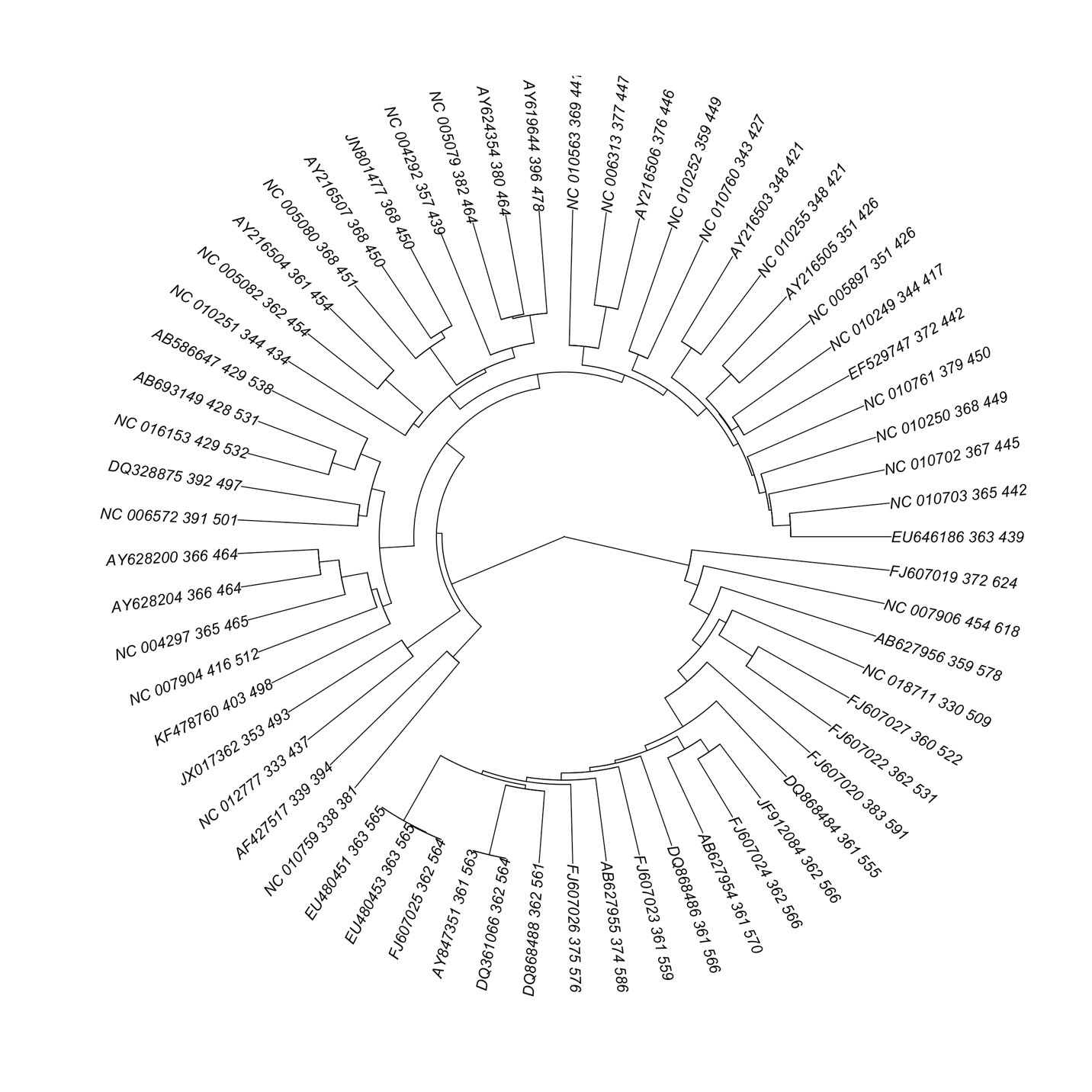 |
| --- | --- |
| b) | 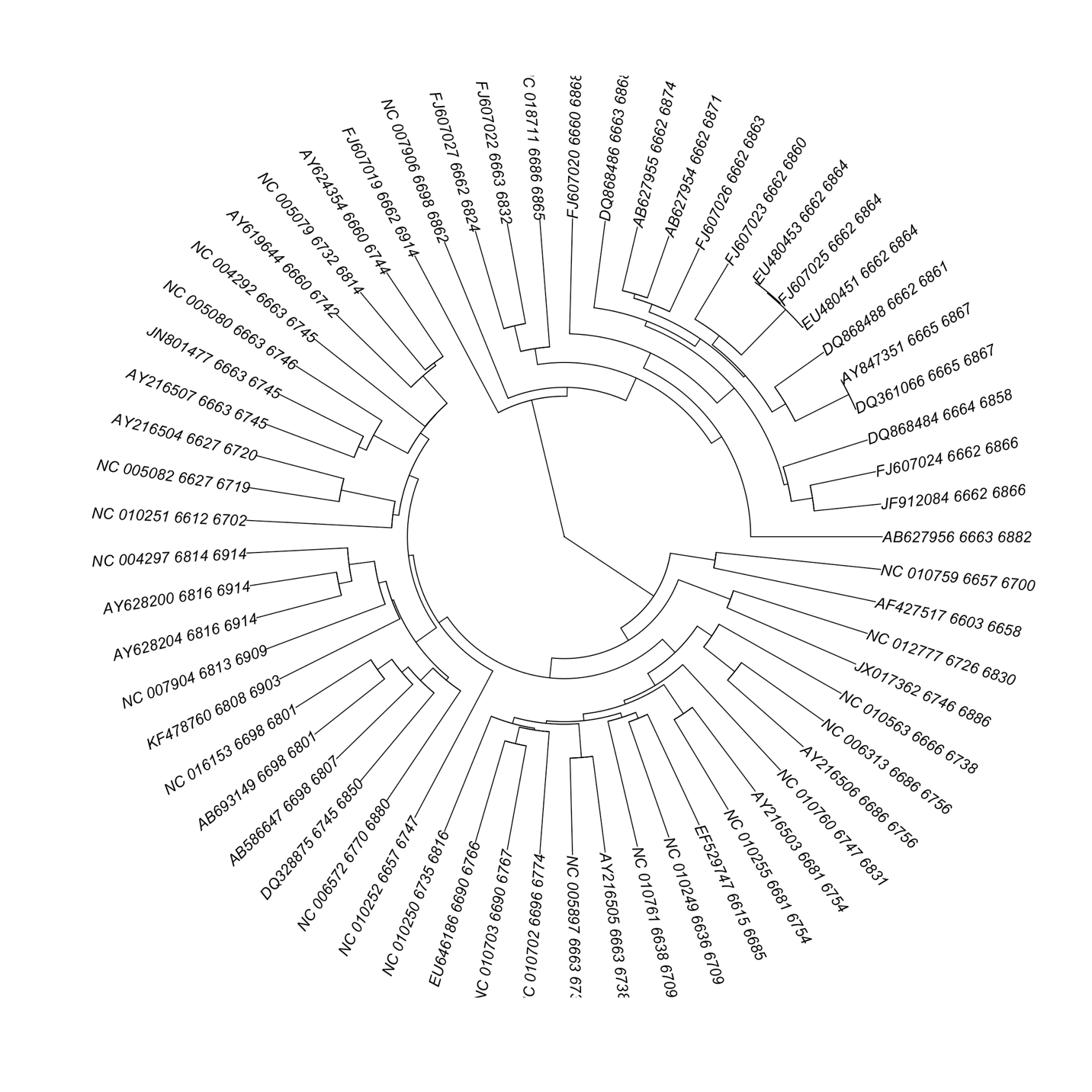 |
| c) | 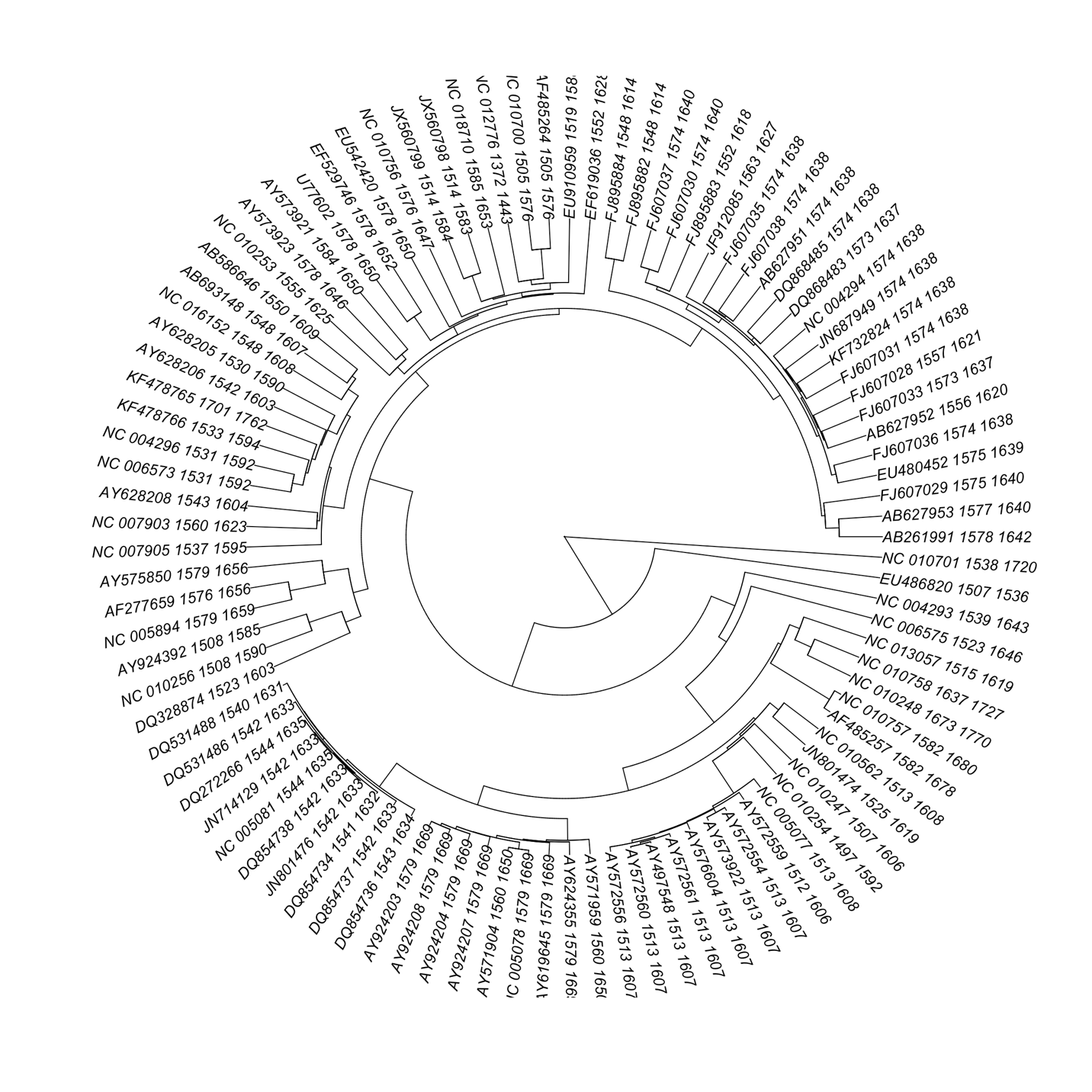 |
| d) | 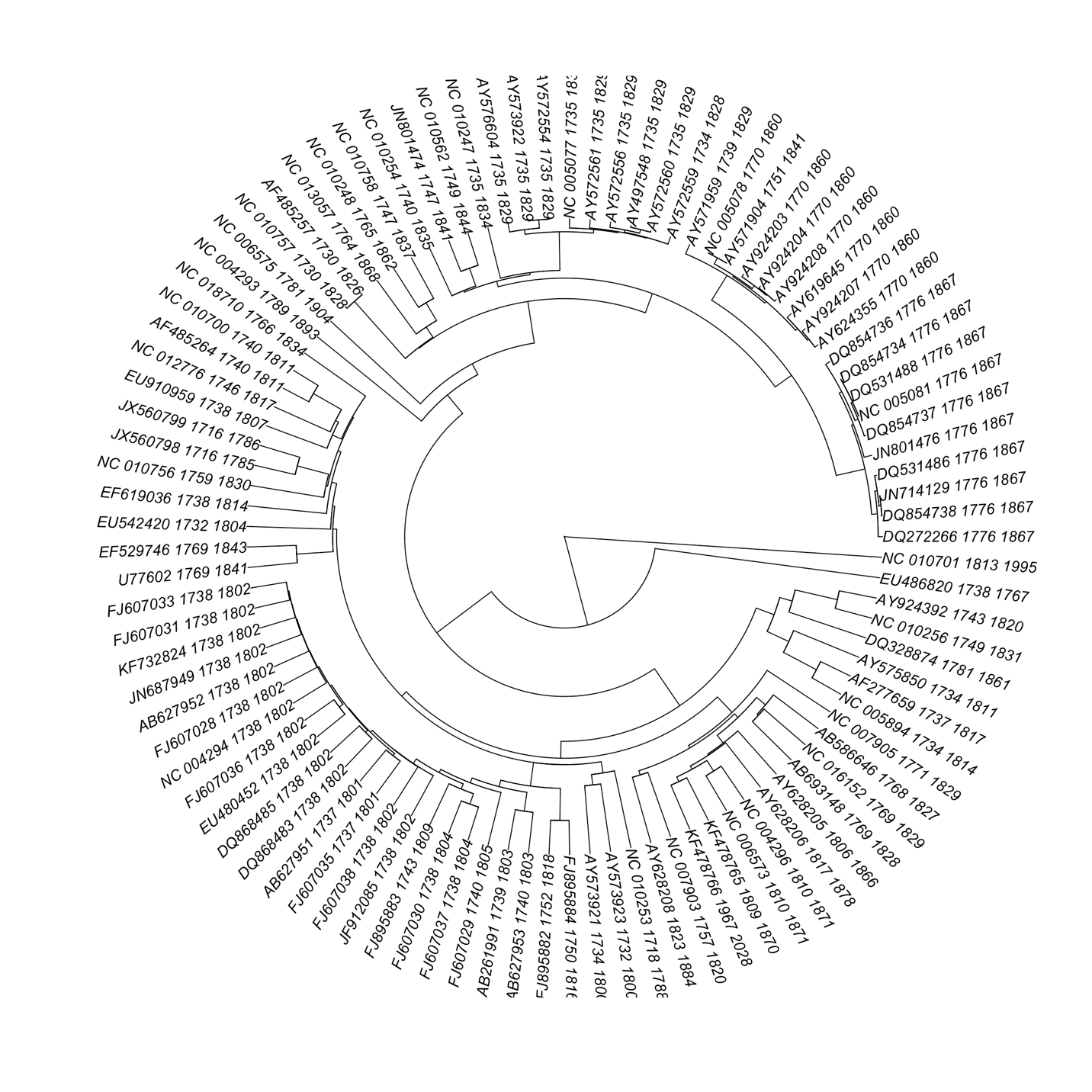 |
| e) | 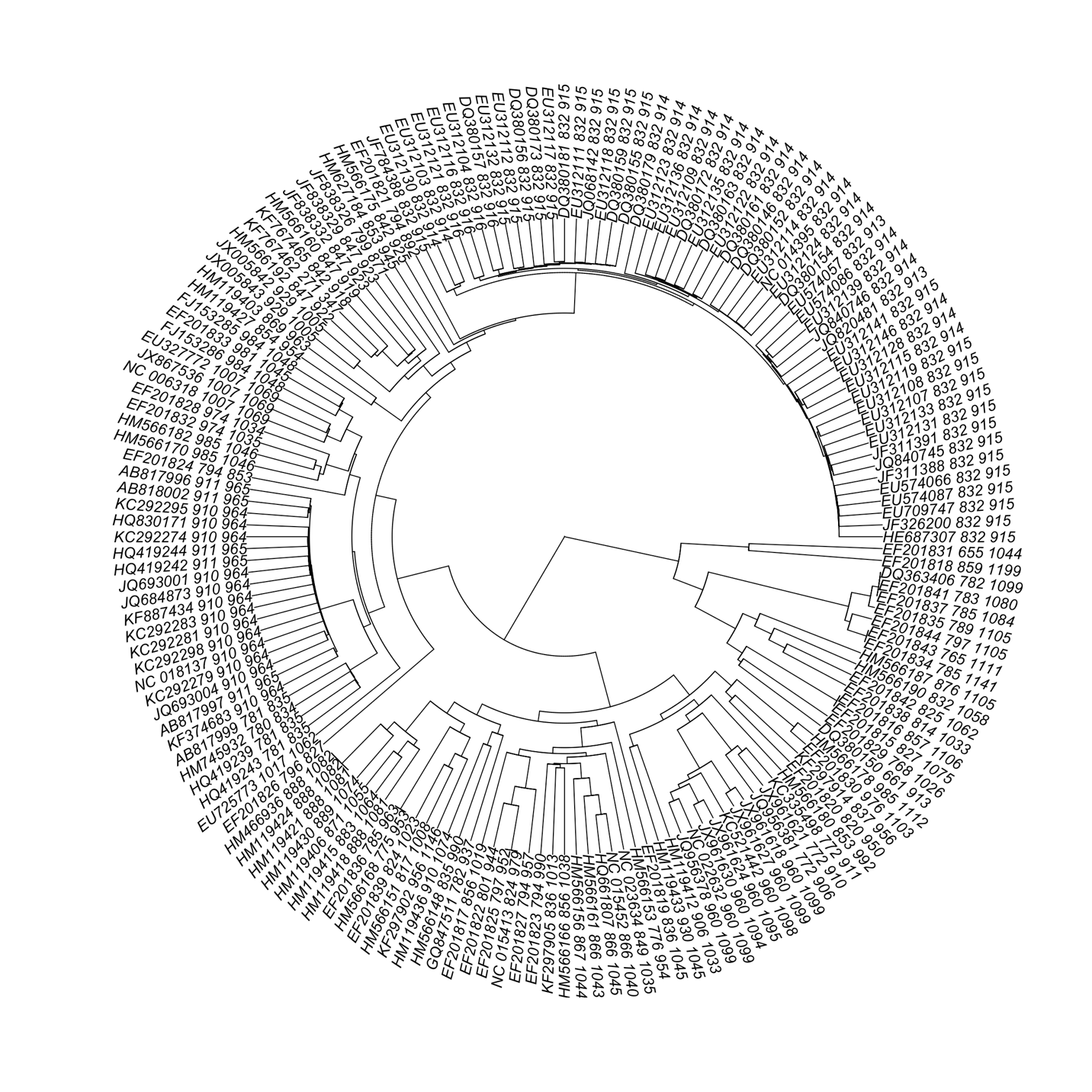 |
| f) | 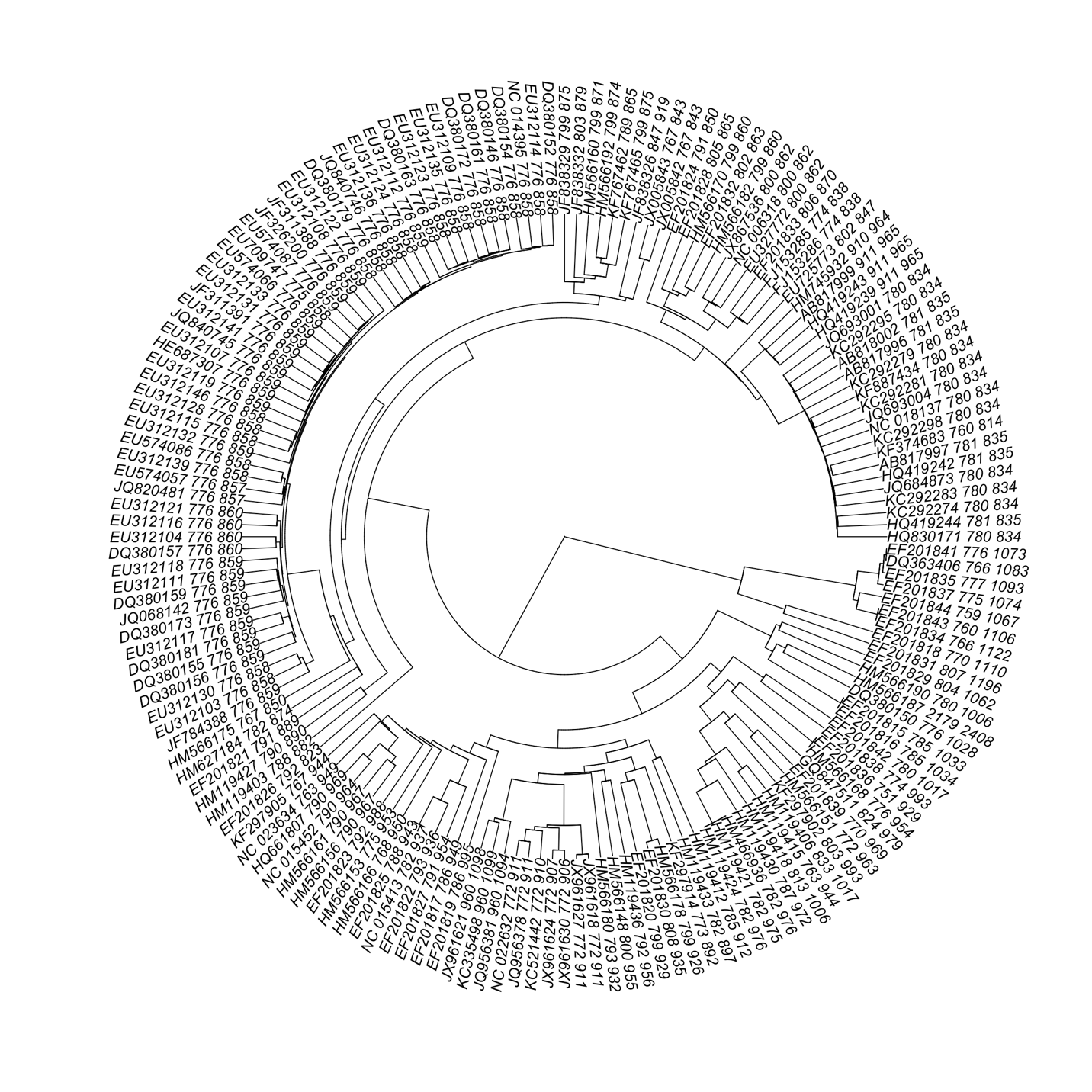 |
| g) | 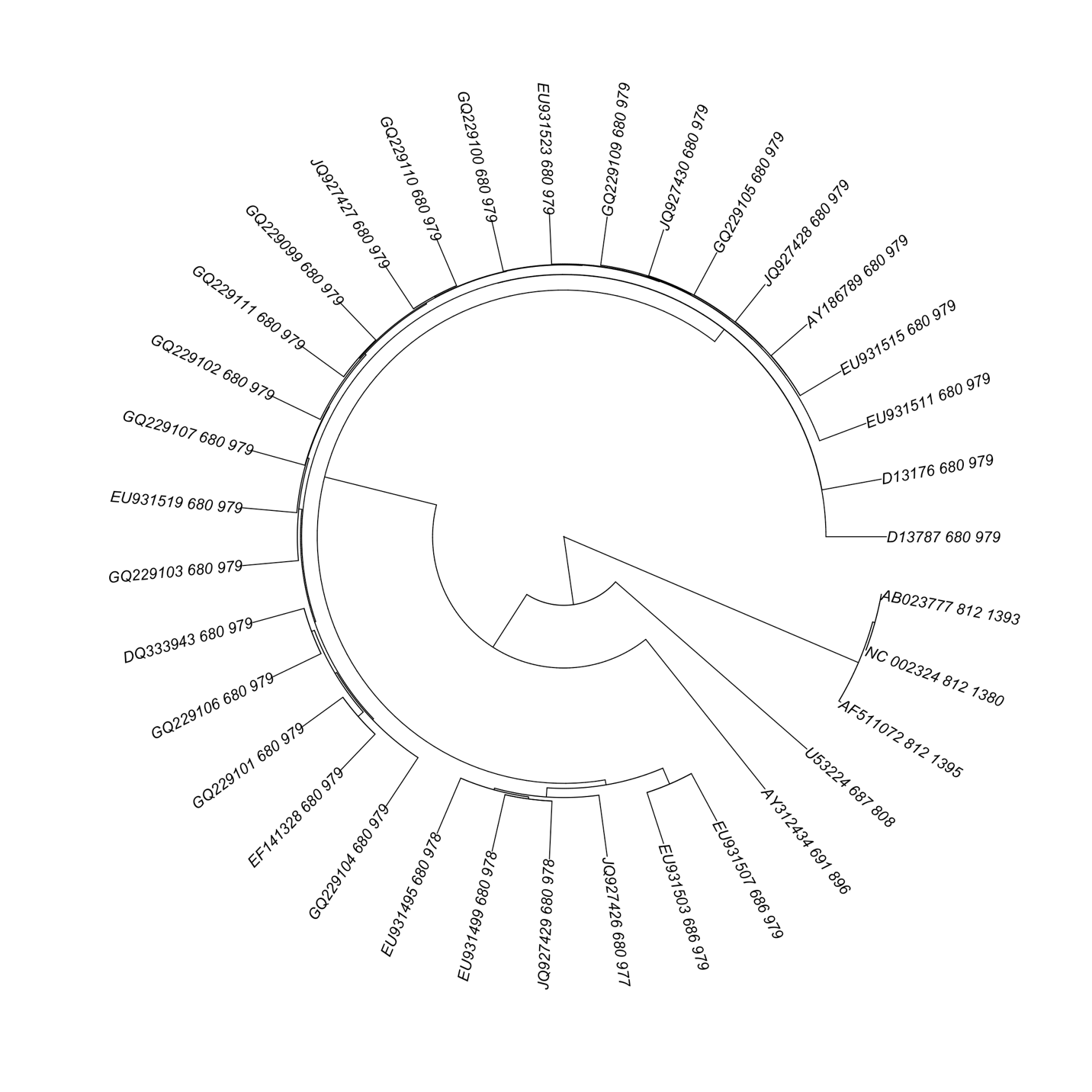 |
| h) | 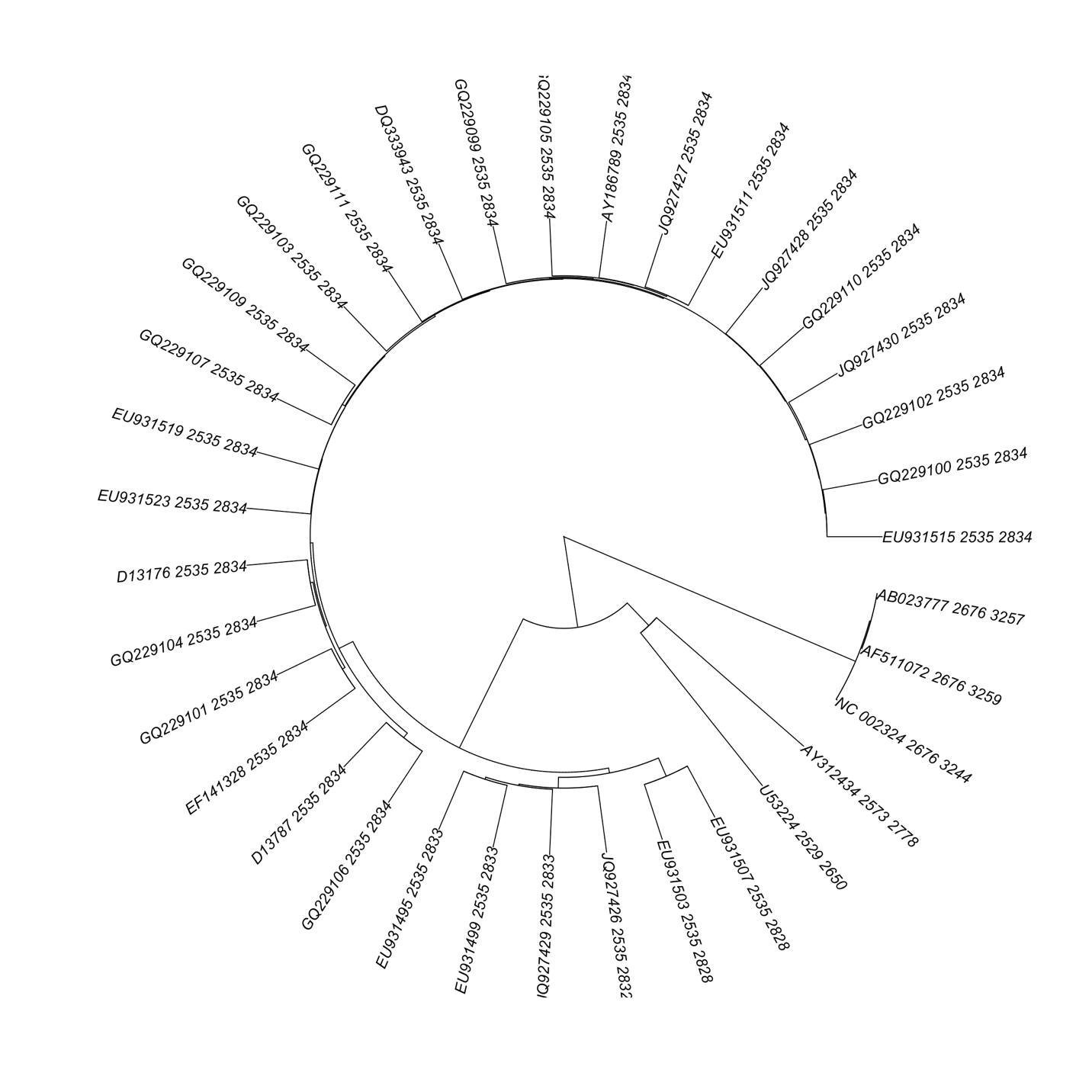 |
| i) | 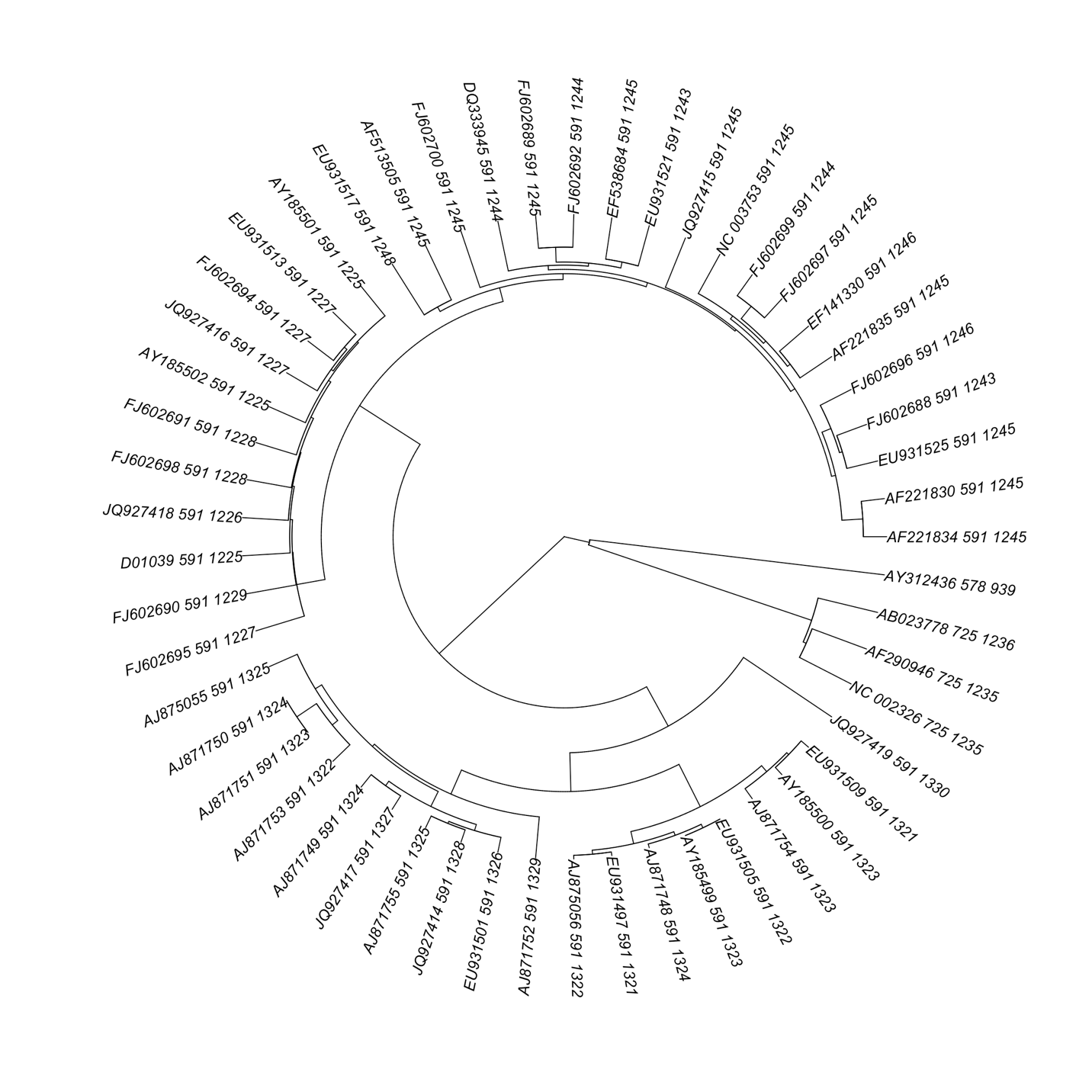 |
| j) | 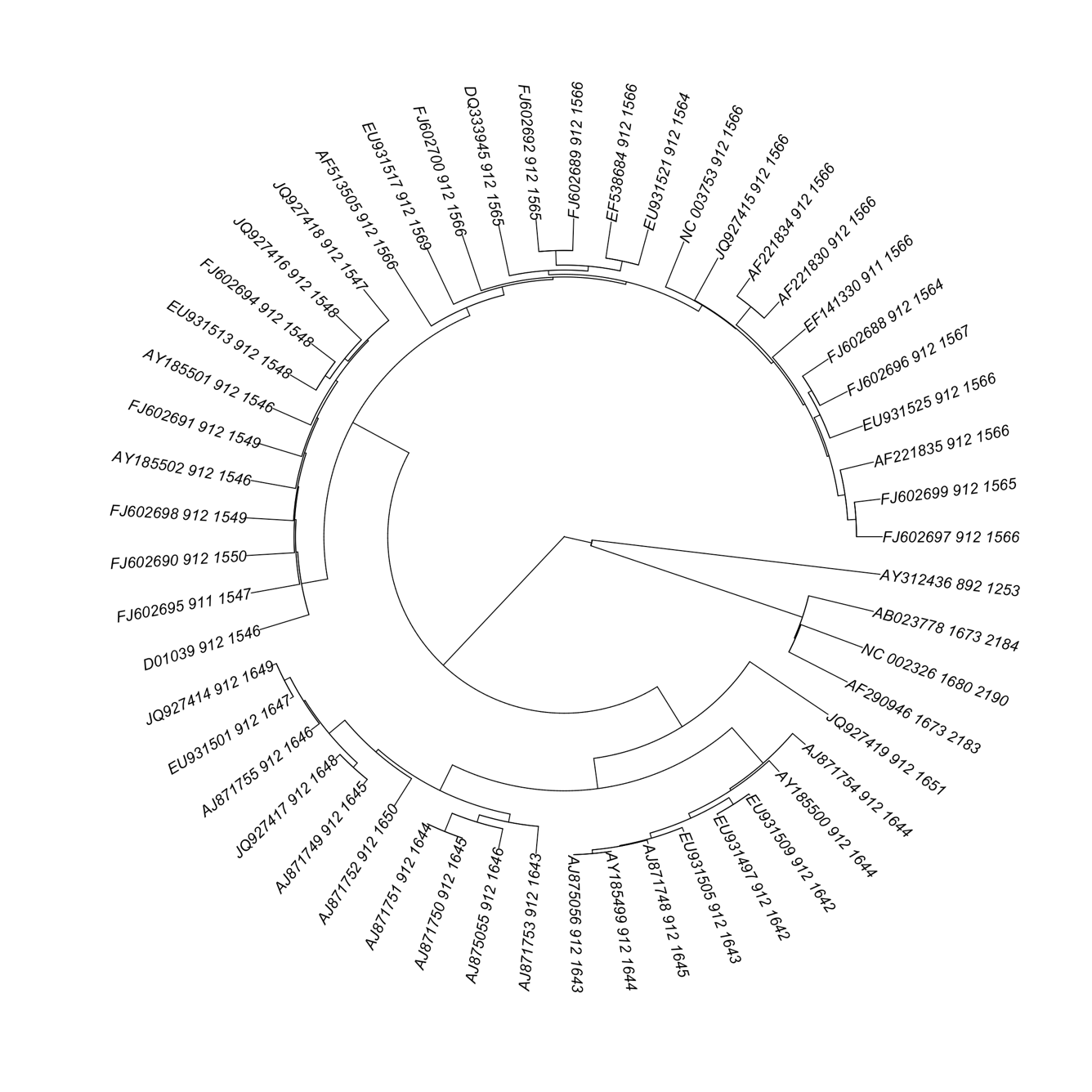 |
| k) | 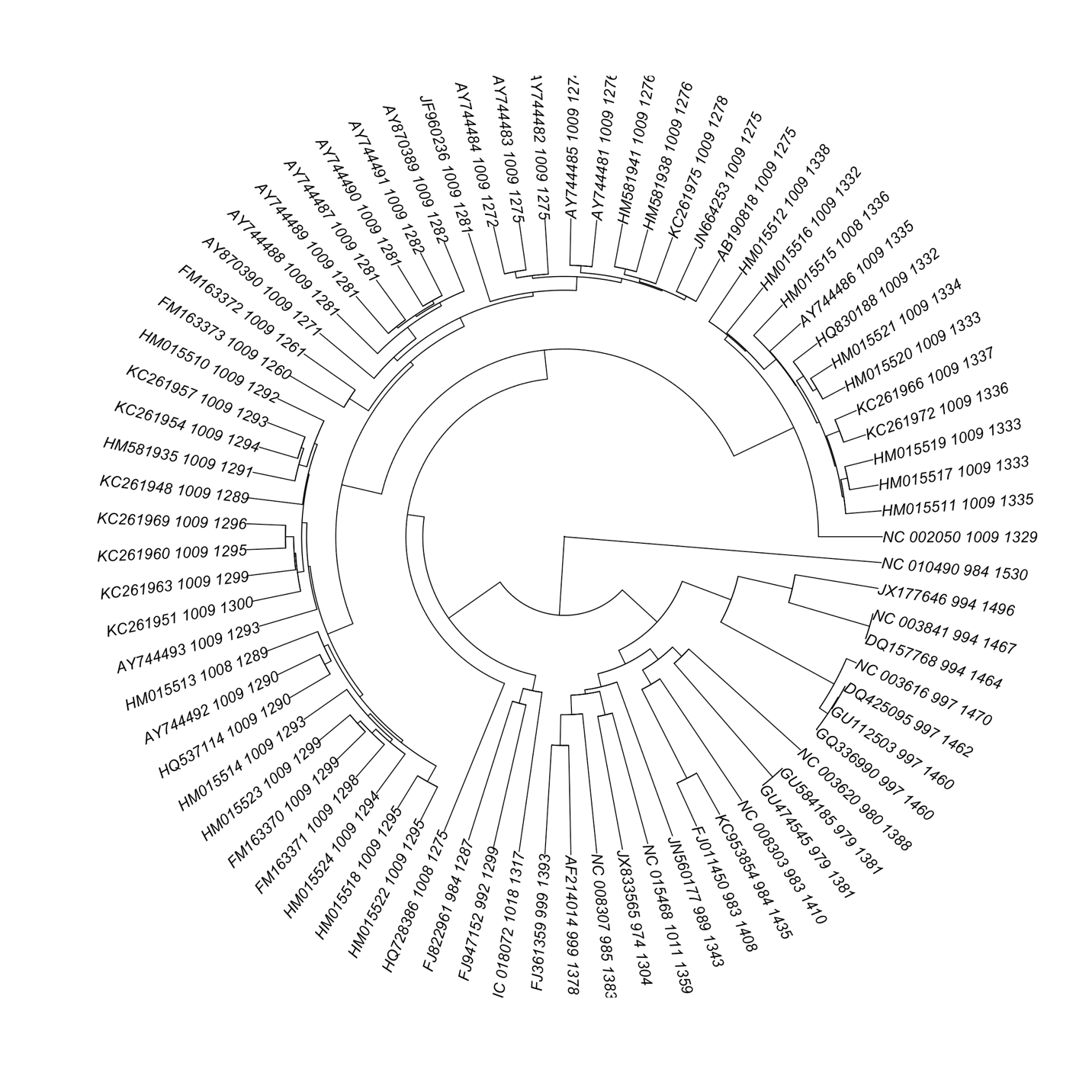 |
| l) | 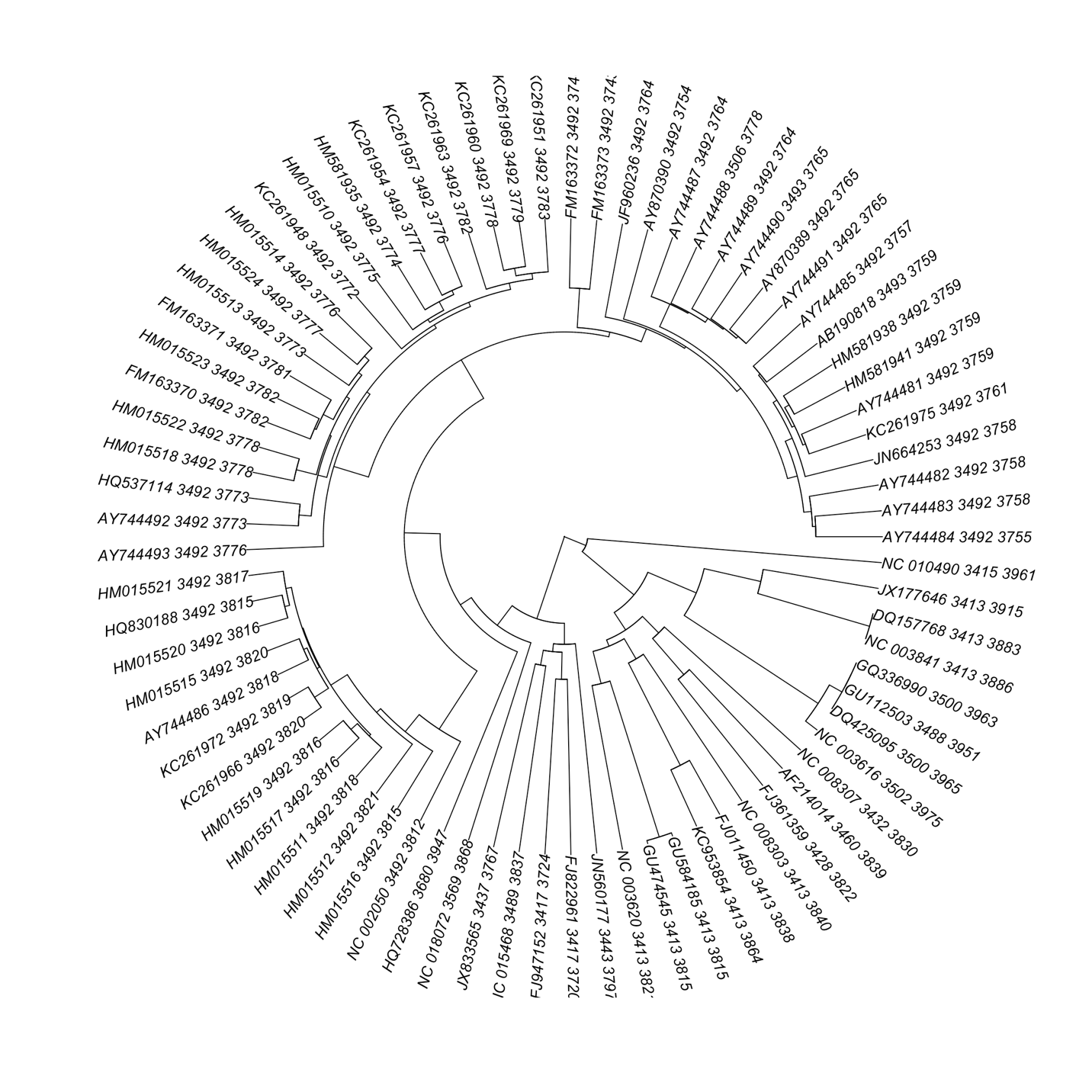 |

S 1: Distance trees calculated by RNAclust. The leafs are annotated with the corresponding REFseq ID followed by the positions of the first and the last base of the IR: a) AV-L-v-IR b) AV-L-vc-IR c) AV-S-v-IR d) AV-S-vc-IR e) PV-S-v-IR f) PV-S-vc-IR g) TEV-2-v-IR h) TEV-2-vc-IR i) TEV-4-v-IR j) TEV-4-vc-IR k) TV-M-v-IR l) TV-M-vc-IR.

| Dataset | ID | Cluster ID | Virus Name | mPID | SCI | z-score |
| --- | --- | --- | --- | --- | --- | --- |
| arenavirus - S | AB261991 | 3 | Lymphocytic choriomeningitis virus | 0.65 | 0.94 | -7.51 |
|  | AB627953 | 3 | Lymphocytic choriomeningitis virus | 0.65 | 0.94 | -7.42 |
|  | FJ607029 | 3 | Lymphocytic choriomeningitis virus | 0.65 | 0.94 | -8.19 |
|  | EU480452 | 3 | Lymphocytic choriomeningitis virus | 0.65 | 0.94 | -9.72 |
|  | FJ607036 | 3 | Lymphocytic choriomeningitis virus | 0.65 | 0.94 | -9.45 |
|  | AB627952 | 3 | Lymphocytic choriomeningitis virus | 0.65 | 0.94 | -9.17 |
|  | FJ607033 | 3 | Lymphocytic choriomeningitis virus | 0.65 | 0.94 | -9.82 |
|  | FJ607028 | 3 | Lymphocytic choriomeningitis virus | 0.65 | 0.94 | -9.59 |
|  | FJ607031 | 3 | Lymphocytic choriomeningitis virus | 0.65 | 0.94 | -9.23 |
|  | KF732824 | 3 | Lymphocytic choriomeningitis virus | 0.65 | 0.94 | -9.39 |
|  | JN687949 | 3 | Lymphocytic choriomeningitis virus | 0.65 | 0.94 | -8.94 |
|  | NC_004294 | 3 | Lymphocytic choriomeningitis virus | 0.65 | 0.94 | -9.03 |
|  | DQ868483 | 3 | Lymphocytic choriomeningitis virus | 0.65 | 0.94 | -8.12 |
|  | DQ868485 | 3 | Lymphocytic choriomeningitis virus | 0.65 | 0.94 | -8.56 |
|  | AB627951 | 3 | Lymphocytic choriomeningitis virus | 0.65 | 0.94 | -9.68 |
|  | FJ607038 | 3 | Lymphocytic choriomeningitis virus | 0.65 | 0.94 | -8.2 |
|  | FJ607035 | 3 | Lymphocytic choriomeningitis virus | 0.65 | 0.94 | -7.05 |
|  | JF912085 | 3 | Lymphocytic choriomeningitis virus | 0.65 | 0.94 | -6.24 |
|  | FJ895883 | 3 | Lymphocytic choriomeningitis virus | 0.65 | 0.94 | -8.45 |
|  | FJ607030 | 3 | Lymphocytic choriomeningitis virus | 0.65 | 0.94 | -9.24 |
|  | FJ607037 | 3 | Lymphocytic choriomeningitis virus | 0.65 | 0.94 | -8.86 |
|  | FJ895882 | 3 | Lymphocytic choriomeningitis virus | 0.65 | 0.94 | -7.17 |
|  | FJ895884 | 3 | Lymphocytic choriomeningitis virus | 0.65 | 0.94 | -7.35 |
|  | EF619036 | 3 | Big brushy tank virus | 0.65 | 0.94 | -7.38 |
|  | EU910959 | 3 | Arenavirus H0380005 | 0.65 | 0.94 | -8.04 |
|  | AF485264 | 3 | Whitewater Arroyo virus | 0.65 | 0.94 | -9.36 |
|  | NC_010700 | 3 | Whitewater Arroyo virus | 0.65 | 0.94 | -7.59 |
|  | NC_012776 | 3 | Lujo virus | 0.65 | 0.94 | -9.19 |
|  | NC_018710 | 3 | Lunk virus NKS-1 | 0.65 | 0.94 | -7.02 |
|  | JX560798 | 3 | North American arenavirus | 0.65 | 0.94 | -8.17 |
|  | JX560799 | 3 | North American arenavirus | 0.65 | 0.94 | -7.86 |
|  | NC_010756 | 3 | Parana virus | 0.65 | 0.94 | -8.27 |
|  | EU542420 | 3 | Pirital virus | 0.65 | 0.94 | -8.56 |
|  | EF529746 | 3 | Pichinde virus | 0.65 | 0.94 | -8.63 |
|  | U77602 | 3 | Pichinde virus | 0.65 | 0.94 | -6.49 |
|  | AY573921 | 3 | Pirital virus | 0.65 | 0.94 | -6.24 |
|  | AY573923 | 3 | Pirital virus | 0.65 | 0.94 | -8.67 |
|  | NC_010253 | 3 | Allpahuayo virus | 0.65 | 0.94 | -8.75 |
|  | AB586646 | 3 | Luna virus | 0.65 | 0.94 | -8.03 |
|  | AB693148 | 3 | Luna virus | 0.65 | 0.94 | -9.01 |
|  | NC_016152 | 3 | Luna virus | 0.65 | 0.94 | -7.43 |
|  | AY628205 | 3 | Lassa virus | 0.65 | 0.94 | -7.21 |
|  | AY628206 | 3 | Lassa virus | 0.65 | 0.94 | -8.07 |
|  | KF478765 | 3 | Lassa virus | 0.65 | 0.94 | -8.11 |
|  | KF478766 | 3 | Lassa virus | 0.65 | 0.94 | -8.56 |
|  | NC_004296 | 3 | Lassa virus | 0.65 | 0.94 | -8.14 |
|  | NC_006573 | 3 | Mopeia Lassa virus reassortant 29 | 0.65 | 0.94 | -8.14 |
|  | AY628208 | 3 | Lassa virus | 0.65 | 0.94 | -8.07 |
|  | NC_007903 | 3 | Mobala virus | 0.65 | 0.94 | -8.2 |
|  | NC_007905 | 3 | Ippy virus | 0.65 | 0.94 | -9.19 |
|  | AY575850 | 3 | Pirital virus | 0.65 | 0.94 | -8.05 |
|  | AF277659 | 3 | Pirital virus | 0.65 | 0.94 | -6.13 |
|  | NC_005894 | 3 | Pirital virus | 0.65 | 0.94 | -7.16 |
|  | AY924392 | 3 | Bear Canyon virus | 0.65 | 0.94 | -8.48 |
|  | NC_010256 | 3 | Bear Canyon virus | 0.65 | 0.94 | -9.04 |
|  | DQ328874 | 3 | Mopeia virus | 0.65 | 0.94 | -6.11 |
|  | DQ531488 | 119 | Junin virus | 0.95 | 1.01 | -6.23 |
|  | DQ531486 | 119 | Junin virus | 0.95 | 1.01 | -7.32 |
|  | DQ272266 | 119 | Junin virus | 0.95 | 1.01 | -7.25 |
|  | JN714129 | 119 | Junin virus | 0.95 | 1.01 | -7.2 |
|  | NC_005081 | 119 | Junin virus | 0.95 | 1.01 | -6.81 |
|  | DQ854738 | 119 | Junin virus | 0.95 | 1.01 | -7.4 |
|  | JN801476 | 119 | Junin virus | 0.95 | 1.01 | -6.55 |
|  | DQ854734 | 119 | Junin virus | 0.95 | 1.01 | -6.84 |
|  | DQ854737 | 119 | Junin virus | 0.95 | 1.01 | -7.09 |
|  | DQ854736 | 119 | Junin virus | 0.95 | 1.01 | -8.23 |
|  | AY924203 | 138 | Machupo virus | 0.94 | 0.99 | -7.96 |
|  | AY924208 | 138 | Machupo virus | 0.94 | 0.99 | -7.91 |
|  | AY924204 | 138 | Machupo virus | 0.94 | 0.99 | -8.42 |
|  | AY924207 | 138 | Machupo virus | 0.94 | 0.99 | -9.09 |
|  | AY571904 | 138 | Machupo virus | 0.94 | 0.99 | -8.28 |
|  | NC_005078 | 138 | Machupo virus | 0.94 | 0.99 | -8.28 |
|  | AY619645 | 138 | Machupo virus | 0.94 | 0.99 | -7.97 |
|  | AY624355 | 138 | Machupo virus | 0.94 | 0.99 | -8.56 |
|  | AY571959 | 138 | Machupo virus | 0.94 | 0.99 | -8.62 |
|  | AY572556 | 155 | Guanarito virus | 0.76 | 0.97 | -10.58 |
|  | AY572560 | 155 | Guanarito virus | 0.76 | 0.97 | -10.89 |
|  | AY497548 | 155 | Guanarito virus | 0.76 | 0.97 | -10.88 |
|  | AY572561 | 155 | Guanarito virus | 0.76 | 0.97 | -10.04 |
|  | AY576604 | 155 | Guanarito virus | 0.76 | 0.97 | -8.91 |
|  | AY573922 | 155 | Guanarito virus | 0.76 | 0.97 | -8.58 |
|  | AY572554 | 155 | Guanarito virus | 0.76 | 0.97 | -9.28 |
|  | AY572559 | 155 | Guanarito virus | 0.76 | 0.97 | -9.7 |
|  | NC_005077 | 155 | Guanarito virus | 0.76 | 0.97 | -10.16 |
|  | NC_010254 | 155 | Cupixi virus | 0.76 | 0.97 | -8.04 |
|  | NC_010247 | 155 | Amapari virus | 0.76 | 0.97 | -9.46 |
|  | JN801474 | 155 | Sabia virus | 0.76 | 0.97 | -9.25 |
|  | NC_010562 | 155 | Chapare virus | 0.76 | 0.97 | -8.72 |
|  | AF485257 | 180 | Flexal virus | 0.68 | 1.02 | -9.39 |
|  | NC_010757 | 180 | Flexal virus | 0.68 | 1.02 | -8.31 |
|  | NC_010248 | 180 | Oliveros virus | 0.68 | 1.02 | -7.52 |
|  | NC_010758 | 180 | Latino virus | 0.68 | 1.02 | -9.65 |
|  | NC_013057 | 180 | Morogoro virus | 0.68 | 1.02 | -10.05 |
|  | NC_006575 | 189 | Mopeia virus AN20410 | -100 | -100 | null |
|  | NC_004293 | 190 | Tacaribe virus | -100 | -100 | null |
|  | EU486820 | 191 | Arenavirus AV 96010025 | -100 | -100 | null |
|  | NC_010701 | 192 | Tamiami virus | -100 | -100 | null |
| arenavirus - S_complement | DQ272266 | 8 | Junin virus | 0.95 | 0.98 | -5.74 |
|  | DQ854738 | 8 | Junin virus | 0.95 | 0.98 | -6.42 |
|  | JN714129 | 8 | Junin virus | 0.95 | 0.98 | -6.45 |
|  | DQ531486 | 8 | Junin virus | 0.95 | 0.98 | -6.35 |
|  | JN801476 | 8 | Junin virus | 0.95 | 0.98 | -5.72 |
|  | DQ854737 | 8 | Junin virus | 0.95 | 0.98 | -7.87 |
|  | NC_005081 | 8 | Junin virus | 0.95 | 0.98 | -6.77 |
|  | DQ531488 | 8 | Junin virus | 0.95 | 0.98 | -6.92 |
|  | DQ854734 | 8 | Junin virus | 0.95 | 0.98 | -7.26 |
|  | DQ854736 | 8 | Junin virus | 0.95 | 0.98 | -7.55 |
|  | AY624355 | 27 | Machupo virus | 0.88 | 0.98 | -7.69 |
|  | AY924207 | 27 | Machupo virus | 0.88 | 0.98 | -9.12 |
|  | AY619645 | 27 | Machupo virus | 0.88 | 0.98 | -7.83 |
|  | AY924208 | 27 | Machupo virus | 0.88 | 0.98 | -6.59 |
|  | AY924204 | 27 | Machupo virus | 0.88 | 0.98 | -7.04 |
|  | AY924203 | 27 | Machupo virus | 0.88 | 0.98 | -5.7 |
|  | AY571904 | 27 | Machupo virus | 0.88 | 0.98 | -7.39 |
|  | NC_005078 | 27 | Machupo virus | 0.88 | 0.98 | -7.39 |
|  | AY571959 | 27 | Machupo virus | 0.88 | 0.98 | -7.09 |
|  | AY572559 | 47 | Guanarito virus | 0.93 | 0.96 | -7.59 |
|  | AY572560 | 47 | Guanarito virus | 0.93 | 0.96 | -9.25 |
|  | AY497548 | 47 | Guanarito virus | 0.93 | 0.96 | -9.09 |
|  | AY572556 | 47 | Guanarito virus | 0.93 | 0.96 | -8.57 |
|  | AY572561 | 47 | Guanarito virus | 0.93 | 0.96 | -7.35 |
|  | NC_005077 | 47 | Guanarito virus | 0.93 | 0.96 | -8.29 |
|  | AY572554 | 58 | Guanarito virus | 0.93 | 0.98 | -6.85 |
|  | AY573922 | 58 | Guanarito virus | 0.93 | 0.98 | -6.47 |
|  | AY576604 | 58 | Guanarito virus | 0.93 | 0.98 | -6.93 |
|  | NC_010247 | 63 | Amapari virus | -100 | -100 | null |
|  | NC_010562 | 64 | Chapare virus | 0.69 | 1.03 | -8.59 |
|  | JN801474 | 64 | Sabia virus | 0.69 | 1.03 | -9.22 |
|  | NC_010254 | 64 | Cupixi virus | 0.69 | 1.03 | -5.78 |
|  | NC_010758 | 69 | Latino virus | 0.46 | 1.01 | -9.57 |
|  | NC_010248 | 69 | Oliveros virus | 0.46 | 1.01 | -8.49 |
|  | NC_013057 | 69 | Morogoro virus | 0.46 | 1.01 | -11.64 |
|  | AF485257 | 69 | Flexal virus | 0.46 | 1.01 | -8.53 |
|  | NC_010757 | 69 | Flexal virus | 0.46 | 1.01 | -7.75 |
|  | NC_006575 | 78 | Mopeia virus AN20410 | -100 | -100 | null |
|  | NC_004293 | 79 | Tacaribe virus | -100 | -100 | null |
|  | NC_018710 | 83 | Lunk virus NKS-1 | 0.56 | 0.93 | -8 |
|  | NC_010700 | 83 | Whitewater Arroyo virus | 0.56 | 0.93 | -6.32 |
|  | AF485264 | 83 | Whitewater Arroyo virus | 0.56 | 0.93 | -7.85 |
|  | NC_012776 | 83 | Lujo virus | 0.56 | 0.93 | -9.99 |
|  | EU910959 | 83 | Arenavirus H0380005 | 0.56 | 0.93 | -8.49 |
|  | JX560799 | 83 | North American arenavirus | 0.56 | 0.93 | -8.66 |
|  | JX560798 | 83 | North American arenavirus | 0.56 | 0.93 | -9.25 |
|  | NC_010756 | 83 | Parana virus | 0.56 | 0.93 | -7.89 |
|  | EF619036 | 83 | Big brushy tank virus | 0.56 | 0.93 | -7.39 |
|  | EU542420 | 83 | Pirital virus | 0.56 | 0.93 | -7.16 |
|  | EF529746 | 83 | Pichinde virus | 0.56 | 0.93 | -8.93 |
|  | U77602 | 83 | Pichinde virus | 0.56 | 0.93 | -6.32 |
|  | FJ607033 | 107 | Lymphocytic choriomeningitis virus | 0.92 | 0.97 | -8.39 |
|  | FJ607031 | 107 | Lymphocytic choriomeningitis virus | 0.92 | 0.97 | -7.91 |
|  | KF732824 | 107 | Lymphocytic choriomeningitis virus | 0.92 | 0.97 | -7.93 |
|  | JN687949 | 107 | Lymphocytic choriomeningitis virus | 0.92 | 0.97 | -7.7 |
|  | AB627952 | 107 | Lymphocytic choriomeningitis virus | 0.92 | 0.97 | -8.13 |
|  | FJ607028 | 107 | Lymphocytic choriomeningitis virus | 0.92 | 0.97 | -8.06 |
|  | NC_004294 | 107 | Lymphocytic choriomeningitis virus | 0.92 | 0.97 | -7.87 |
|  | FJ607036 | 107 | Lymphocytic choriomeningitis virus | 0.92 | 0.97 | -7.61 |
|  | EU480452 | 107 | Lymphocytic choriomeningitis virus | 0.92 | 0.97 | -7.42 |
|  | DQ868485 | 107 | Lymphocytic choriomeningitis virus | 0.92 | 0.97 | -8.37 |
|  | DQ868483 | 107 | Lymphocytic choriomeningitis virus | 0.92 | 0.97 | -8.23 |
|  | AB627951 | 107 | Lymphocytic choriomeningitis virus | 0.92 | 0.97 | -8.74 |
|  | FJ607035 | 107 | Lymphocytic choriomeningitis virus | 0.92 | 0.97 | -6.8 |
|  | FJ607038 | 107 | Lymphocytic choriomeningitis virus | 0.92 | 0.97 | -8.5 |
|  | JF912085 | 107 | Lymphocytic choriomeningitis virus | 0.92 | 0.97 | -6.93 |
|  | FJ895883 | 107 | Lymphocytic choriomeningitis virus | 0.92 | 0.97 | -9.23 |
|  | FJ607030 | 107 | Lymphocytic choriomeningitis virus | 0.92 | 0.97 | -7.97 |
|  | FJ607037 | 107 | Lymphocytic choriomeningitis virus | 0.92 | 0.97 | -7.72 |
|  | FJ607029 | 107 | Lymphocytic choriomeningitis virus | 0.92 | 0.97 | -7.54 |
|  | AB261991 | 107 | Lymphocytic choriomeningitis virus | 0.92 | 0.97 | -8 |
|  | AB627953 | 107 | Lymphocytic choriomeningitis virus | 0.92 | 0.97 | -8.17 |
|  | FJ895882 | 107 | Lymphocytic choriomeningitis virus | 0.92 | 0.97 | -6.85 |
|  | FJ895884 | 107 | Lymphocytic choriomeningitis virus | 0.92 | 0.97 | -7.17 |
|  | AY573921 | 152 | Pirital virus | 0.68 | 1.07 | -7.63 |
|  | AY573923 | 152 | Pirital virus | 0.68 | 1.07 | -7.24 |
|  | NC_010253 | 152 | Allpahuayo virus | 0.68 | 1.07 | -8.32 |
|  | AY628208 | 157 | Lassa virus | 0.87 | 0.92 | -7.54 |
|  | NC_007903 | 157 | Mobala virus | 0.87 | 0.92 | -7.87 |
|  | KF478766 | 157 | Lassa virus | 0.87 | 0.92 | -8.13 |
|  | KF478765 | 157 | Lassa virus | 0.87 | 0.92 | -7.58 |
|  | NC_006573 | 157 | Mopeia Lassa virus reassortant 29 | 0.87 | 0.92 | -8.19 |
|  | NC_004296 | 157 | Lassa virus | 0.87 | 0.92 | -8.19 |
|  | AY628206 | 157 | Lassa virus | 0.87 | 0.92 | -7.25 |
|  | AY628205 | 157 | Lassa virus | 0.87 | 0.92 | -6.7 |
|  | AB693148 | 157 | Luna virus | 0.87 | 0.92 | -7.66 |
|  | NC_016152 | 157 | Luna virus | 0.87 | 0.92 | -7.53 |
|  | AB586646 | 157 | Luna virus | 0.87 | 0.92 | -7.57 |
|  | NC_007905 | 157 | Ippy virus | 0.87 | 0.92 | -6.09 |
|  | NC_005894 | 180 | Pirital virus | 0.46 | 0.92 | -7.22 |
|  | AF277659 | 180 | Pirital virus | 0.46 | 0.92 | -6.76 |
|  | AY575850 | 180 | Pirital virus | 0.46 | 0.92 | -7.36 |
|  | DQ328874 | 180 | Mopeia virus | 0.46 | 0.92 | -5.99 |
|  | NC_010256 | 180 | Bear Canyon virus | 0.46 | 0.92 | -9.6 |
|  | AY924392 | 180 | Bear Canyon virus | 0.46 | 0.92 | -7.82 |
|  | EU486820 | 191 | Arenavirus AV 96010025 | -100 | -100 | null |
|  | NC_010701 | 192 | Tamiami virus | -100 | -100 | null |
| arenavirus - L | EU646186 | 9 | Whitewater Arroyo virus | 0.65 | 1 | -5.65 |
|  | NC_010703 | 9 | Whitewater Arroyo virus | 0.65 | 1 | -5.53 |
|  | NC_010702 | 9 | Tamiami virus | 0.65 | 1 | -5.27 |
|  | NC_010250 | 9 | Oliveros virus | 0.65 | 1 | -5.29 |
|  | NC_010761 | 9 | Parana virus | 0.65 | 1 | -5.36 |
|  | EF529747 | 19 | Pichinde virus | 0.43 | 0.97 | -6.32 |
|  | NC_010249 | 19 | Allpahuayo virus | 0.43 | 0.97 | -4.51 |
|  | NC_005897 | 22 | Pirital virus | 0.74 | 0.94 | -3.9 |
|  | AY216505 | 22 | Pirital virus | 0.74 | 0.94 | -3.93 |
|  | NC_010255 | 25 | Bear Canyon virus | 0.69 | 1.01 | -7.89 |
|  | AY216503 | 25 | Bear Canyon virus | 0.69 | 1.01 | -7.49 |
|  | NC_010760 | 28 | Latino virus | 0.29 | 1.04 | -3.84 |
|  | NC_010252 | 28 | Cupixi virus | 0.29 | 1.04 | -2.08 |
|  | AY216506 | 31 | Sabia virus | 0.8 | 0.91 | -2.35 |
|  | NC_006313 | 31 | Sabia virus | 0.8 | 0.91 | -2.35 |
|  | NC_010563 | 31 | Chapare virus | 0.8 | 0.91 | -3.53 |
|  | AY619644 | 38 | Machupo virus | 0.75 | 0.85 | -2.38 |
|  | AY624354 | 38 | Machupo virus | 0.75 | 0.85 | -1.41 |
|  | NC_005079 | 38 | Machupo virus | 0.75 | 0.85 | -3.34 |
|  | NC_004292 | 38 | Tacaribe virus | 0.75 | 0.85 | -2.05 |
|  | JN801477 | 45 | Junin virus | 0.93 | 0.91 | -2.96 |
|  | AY216507 | 45 | Junin virus | 0.93 | 0.91 | -2.96 |
|  | NC_005080 | 45 | Junin virus | 0.93 | 0.91 | -3.45 |
|  | AY216504 | 50 | Guanarito virus | 0.69 | 0.81 | 0.04 |
|  | NC_005082 | 50 | Guanarito virus | 0.69 | 0.81 | -0.38 |
|  | NC_010251 | 50 | Amapari virus | 0.69 | 0.81 | -3.74 |
|  | AB586647 | 55 | Luna virus | 0.57 | 0.74 | -3.91 |
|  | AB693149 | 55 | Luna virus | 0.57 | 0.74 | -1.75 |
|  | NC_016153 | 55 | Luna virus | 0.57 | 0.74 | -2.34 |
|  | DQ328875 | 55 | Mopeia virus | 0.57 | 0.74 | -2.24 |
|  | NC_006572 | 55 | Mopeia Lassa virus reassortant 29 | 0.57 | 0.74 | -2.39 |
|  | AY628200 | 55 | Lassa virus | 0.57 | 0.74 | -1.97 |
|  | AY628204 | 55 | Lassa virus | 0.57 | 0.74 | -1.21 |
|  | NC_004297 | 55 | Lassa virus | 0.57 | 0.74 | -2.13 |
|  | NC_007904 | 55 | Mobala virus | 0.57 | 0.74 | -0.54 |
|  | KF478760 | 55 | Lassa virus | 0.57 | 0.74 | -2.78 |
|  | JX017362 | 74 | Lujo virus | 0.55 | 0.75 | -2.99 |
|  | NC_012777 | 74 | Lujo virus | 0.55 | 0.75 | -1.31 |
|  | AF427517 | 77 | Pichinde virus | 0.19 | 0.76 | -1.04 |
|  | NC_010759 | 77 | Flexal virus | 0.19 | 0.76 | -0.81 |
|  | EU480451 | 92 | Lymphocytic choriomeningitis virus | 0.96 | 0.89 | -3.56 |
|  | EU480453 | 92 | Lymphocytic choriomeningitis virus | 0.96 | 0.89 | -3.05 |
|  | FJ607025 | 92 | Lymphocytic choriomeningitis virus | 0.96 | 0.89 | -3.87 |
|  | AY847351 | 97 | Lymphocytic choriomeningitis virus | 0.9 | 0.74 | -1.29 |
|  | DQ361066 | 97 | Lymphocytic choriomeningitis virus | 0.9 | 0.74 | -1.66 |
|  | DQ868488 | 97 | Lymphocytic choriomeningitis virus | 0.9 | 0.74 | -3.84 |
|  | FJ607026 | 102 | Lymphocytic choriomeningitis virus | -100 | -100 | null |
|  | AB627955 | 103 | Lymphocytic choriomeningitis virus | -100 | -100 | null |
|  | FJ607023 | 104 | Lymphocytic choriomeningitis virus | -100 | -100 | null |
|  | DQ868486 | 105 | Lymphocytic choriomeningitis virus | -100 | -100 | null |
|  | AB627954 | 106 | Lymphocytic choriomeningitis virus | 0.74 | 0.68 | -1.55 |
|  | FJ607024 | 106 | Lymphocytic choriomeningitis virus | 0.74 | 0.68 | -2.44 |
|  | JF912084 | 106 | Lymphocytic choriomeningitis virus | 0.74 | 0.68 | -2.93 |
|  | DQ868484 | 111 | Lymphocytic choriomeningitis virus | -100 | -100 | null |
|  | FJ607020 | 112 | Lymphocytic choriomeningitis virus | -100 | -100 | null |
|  | FJ607022 | 113 | Lymphocytic choriomeningitis virus | 0.58 | 0.58 | -2.5 |
|  | FJ607027 | 113 | Lymphocytic choriomeningitis virus | 0.58 | 0.58 | -0.74 |
|  | NC_018711 | 113 | Lunk virus NKS-1 | 0.58 | 0.58 | -3.51 |
|  | AB627956 | 118 | Lymphocytic choriomeningitis virus | -100 | -100 | null |
|  | NC_007906 | 119 | Ippy virus | -100 | -100 | null |
|  | FJ607019 | 120 | Lymphocytic choriomeningitis virus | -100 | -100 | null |
| arenavirus - L_complement | AB627956 | 5 | Lymphocytic choriomeningitis virus | -100 | -100 | null |
|  | JF912084 | 8 | Lymphocytic choriomeningitis virus | 0.67 | 0.63 | -2.63 |
|  | FJ607024 | 8 | Lymphocytic choriomeningitis virus | 0.67 | 0.63 | -1.38 |
|  | DQ868484 | 8 | Lymphocytic choriomeningitis virus | 0.67 | 0.63 | 0.89 |
|  | DQ361066 | 16 | Lymphocytic choriomeningitis virus | 0.81 | 0.81 | -0.86 |
|  | AY847351 | 16 | Lymphocytic choriomeningitis virus | 0.81 | 0.81 | -0.52 |
|  | DQ868488 | 16 | Lymphocytic choriomeningitis virus | 0.81 | 0.81 | -1.59 |
|  | EU480451 | 21 | Lymphocytic choriomeningitis virus | 0.73 | 0.83 | -3.79 |
|  | FJ607025 | 21 | Lymphocytic choriomeningitis virus | 0.73 | 0.83 | -3.15 |
|  | EU480453 | 21 | Lymphocytic choriomeningitis virus | 0.73 | 0.83 | -2.73 |
|  | FJ607023 | 21 | Lymphocytic choriomeningitis virus | 0.73 | 0.83 | -0.84 |
|  | FJ607026 | 28 | Lymphocytic choriomeningitis virus | 0.61 | 0.83 | -2.39 |
|  | AB627954 | 28 | Lymphocytic choriomeningitis virus | 0.61 | 0.83 | -0.56 |
|  | AB627955 | 28 | Lymphocytic choriomeningitis virus | 0.61 | 0.83 | -1.58 |
|  | DQ868486 | 33 | Lymphocytic choriomeningitis virus | -100 | -100 | null |
|  | FJ607020 | 34 | Lymphocytic choriomeningitis virus | -100 | -100 | null |
|  | NC_018711 | 35 | Lunk virus NKS-1 | 0.53 | 0.63 | -2.85 |
|  | FJ607022 | 35 | Lymphocytic choriomeningitis virus | 0.53 | 0.63 | -1.21 |
|  | FJ607027 | 35 | Lymphocytic choriomeningitis virus | 0.53 | 0.63 | -2.24 |
|  | NC_007906 | 40 | Ippy virus | -100 | -100 | null |
|  | FJ607019 | 41 | Lymphocytic choriomeningitis virus | -100 | -100 | null |
|  | AY624354 | 49 | Machupo virus | 0.87 | 0.79 | -2.34 |
|  | NC_005079 | 49 | Machupo virus | 0.87 | 0.79 | -2.68 |
|  | AY619644 | 49 | Machupo virus | 0.87 | 0.79 | -2.66 |
|  | NC_004292 | 49 | Tacaribe virus | 0.87 | 0.79 | -2.66 |
|  | NC_005080 | 56 | Junin virus | 0.95 | 0.88 | -2.45 |
|  | JN801477 | 56 | Junin virus | 0.95 | 0.88 | -2.31 |
|  | AY216507 | 56 | Junin virus | 0.95 | 0.88 | -2.31 |
|  | AY216504 | 61 | Guanarito virus | 0.63 | 0.7 | -1.51 |
|  | NC_005082 | 61 | Guanarito virus | 0.63 | 0.7 | -1.99 |
|  | NC_010251 | 61 | Amapari virus | 0.63 | 0.7 | -2.85 |
|  | NC_004297 | 66 | Lassa virus | 0.64 | 0.68 | -2.6 |
|  | AY628200 | 66 | Lassa virus | 0.64 | 0.68 | -2.66 |
|  | AY628204 | 66 | Lassa virus | 0.64 | 0.68 | -2.16 |
|  | NC_007904 | 66 | Mobala virus | 0.64 | 0.68 | -2.1 |
|  | KF478760 | 66 | Lassa virus | 0.64 | 0.68 | -1.66 |
|  | NC_016153 | 66 | Luna virus | 0.64 | 0.68 | -1.16 |
|  | AB693149 | 66 | Luna virus | 0.64 | 0.68 | -2.09 |
|  | AB586647 | 66 | Luna virus | 0.64 | 0.68 | -2.94 |
|  | DQ328875 | 66 | Mopeia virus | 0.64 | 0.68 | -2.58 |
|  | NC_006572 | 66 | Mopeia Lassa virus reassortant 29 | 0.64 | 0.68 | -3.13 |
|  | NC_010252 | 85 | Cupixi virus | -100 | -100 | null |
|  | NC_010250 | 90 | Oliveros virus | 0.77 | 1 | -4.54 |
|  | EU646186 | 90 | Whitewater Arroyo virus | 0.77 | 1 | -5.2 |
|  | NC_010703 | 90 | Whitewater Arroyo virus | 0.77 | 1 | -5.75 |
|  | NC_010702 | 90 | Tamiami virus | 0.77 | 1 | -5.08 |
|  | NC_005897 | 90 | Pirital virus | 0.77 | 1 | -4.22 |
|  | AY216505 | 90 | Pirital virus | 0.77 | 1 | -4.22 |
|  | NC_010761 | 101 | Parana virus | 0.56 | 0.99 | -5.09 |
|  | NC_010249 | 101 | Allpahuayo virus | 0.56 | 0.99 | -3.48 |
|  | EF529747 | 101 | Pichinde virus | 0.56 | 0.99 | -5.3 |
|  | NC_010255 | 106 | Bear Canyon virus | 0.69 | 1.02 | -8.92 |
|  | AY216503 | 106 | Bear Canyon virus | 0.69 | 1.02 | -8.49 |
|  | NC_010760 | 109 | Latino virus | -100 | -100 | null |
|  | AY216506 | 110 | Sabia virus | 0.8 | 0.91 | -3 |
|  | NC_006313 | 110 | Sabia virus | 0.8 | 0.91 | -3 |
|  | NC_010563 | 110 | Chapare virus | 0.8 | 0.91 | -3.56 |
|  | JX017362 | 115 | Lujo virus | 0.55 | 0.79 | -3.12 |
|  | NC_012777 | 115 | Lujo virus | 0.55 | 0.79 | -1.37 |
|  | AF427517 | 118 | Pichinde virus | 0.27 | 0.98 | -0.96 |
|  | NC_010759 | 118 | Flexal virus | 0.27 | 0.98 | -1.69 |
| tospovirus - M | NC_002050 | 5 | Tomato spotted wilt virus | 0.89 | 0.67 | -3.08 |
|  | HM015511 | 5 | Tomato spotted wilt virus | 0.89 | 0.67 | -3.15 |
|  | HM015517 | 5 | Tomato spotted wilt virus | 0.89 | 0.67 | -3.86 |
|  | HM015519 | 5 | Tomato spotted wilt virus | 0.89 | 0.67 | -3.38 |
|  | KC261972 | 5 | Tomato spotted wilt virus | 0.89 | 0.67 | -4.22 |
|  | KC261966 | 5 | Tomato spotted wilt virus | 0.89 | 0.67 | -4 |
|  | HM015520 | 5 | Tomato spotted wilt virus | 0.89 | 0.67 | -3.08 |
|  | HM015521 | 5 | Tomato spotted wilt virus | 0.89 | 0.67 | -2.81 |
|  | HQ830188 | 5 | Tomato spotted wilt virus | 0.89 | 0.67 | -4.65 |
|  | AY744486 | 5 | Tomato spotted wilt virus | 0.89 | 0.67 | -2.49 |
|  | HM015515 | 5 | Tomato spotted wilt virus | 0.89 | 0.67 | -2.87 |
|  | HM015516 | 5 | Tomato spotted wilt virus | 0.89 | 0.67 | -4.23 |
|  | HM015512 | 5 | Tomato spotted wilt virus | 0.89 | 0.67 | -4.65 |
|  | AB190818 | 31 | Tomato spotted wilt virus | 0.88 | 0.61 | -3.76 |
|  | JN664253 | 31 | Tomato spotted wilt virus | 0.88 | 0.61 | -3.46 |
|  | KC261975 | 31 | Tomato spotted wilt virus | 0.88 | 0.61 | -3.68 |
|  | HM581938 | 31 | Tomato spotted wilt virus | 0.88 | 0.61 | -4.1 |
|  | HM581941 | 31 | Tomato spotted wilt virus | 0.88 | 0.61 | -4.16 |
|  | AY744481 | 31 | Tomato spotted wilt virus | 0.88 | 0.61 | -4.92 |
|  | AY744485 | 31 | Tomato spotted wilt virus | 0.88 | 0.61 | -5.24 |
|  | AY744482 | 31 | Tomato spotted wilt virus | 0.88 | 0.61 | -3.62 |
|  | AY744483 | 31 | Tomato spotted wilt virus | 0.88 | 0.61 | -3.89 |
|  | AY744484 | 31 | Tomato spotted wilt virus | 0.88 | 0.61 | -3.69 |
|  | JF960236 | 31 | Tomato spotted wilt virus | 0.88 | 0.61 | -4.16 |
|  | AY870389 | 31 | Tomato spotted wilt virus | 0.88 | 0.61 | -1.33 |
|  | AY744491 | 31 | Tomato spotted wilt virus | 0.88 | 0.61 | -1.71 |
|  | AY744490 | 31 | Tomato spotted wilt virus | 0.88 | 0.61 | -1.92 |
|  | AY744487 | 31 | Tomato spotted wilt virus | 0.88 | 0.61 | -1.51 |
|  | AY744489 | 31 | Tomato spotted wilt virus | 0.88 | 0.61 | -1.57 |
|  | AY744488 | 31 | Tomato spotted wilt virus | 0.88 | 0.61 | -1.21 |
|  | AY870390 | 31 | Tomato spotted wilt virus | 0.88 | 0.61 | -2.23 |
|  | FM163372 | 31 | Tomato spotted wilt virus | 0.88 | 0.61 | -3.27 |
|  | FM163373 | 31 | Tomato spotted wilt virus | 0.88 | 0.61 | -2.9 |
|  | HM015510 | 71 | Tomato spotted wilt virus | 0.96 | 0.82 | -3.32 |
|  | KC261957 | 71 | Tomato spotted wilt virus | 0.96 | 0.82 | -2.78 |
|  | KC261954 | 71 | Tomato spotted wilt virus | 0.96 | 0.82 | -2.96 |
|  | HM581935 | 71 | Tomato spotted wilt virus | 0.96 | 0.82 | -2.96 |
|  | KC261948 | 71 | Tomato spotted wilt virus | 0.96 | 0.82 | -1.62 |
|  | KC261969 | 71 | Tomato spotted wilt virus | 0.96 | 0.82 | -2.94 |
|  | KC261960 | 71 | Tomato spotted wilt virus | 0.96 | 0.82 | -1.88 |
|  | KC261963 | 71 | Tomato spotted wilt virus | 0.96 | 0.82 | -3.15 |
|  | KC261951 | 71 | Tomato spotted wilt virus | 0.96 | 0.82 | -1.74 |
|  | AY744493 | 71 | Tomato spotted wilt virus | 0.96 | 0.82 | -2.1 |
|  | HM015513 | 90 | Tomato spotted wilt virus | 0.97 | 0.81 | -2.64 |
|  | AY744492 | 90 | Tomato spotted wilt virus | 0.97 | 0.81 | -2.35 |
|  | HQ537114 | 90 | Tomato spotted wilt virus | 0.97 | 0.81 | -1.6 |
|  | HM015514 | 90 | Tomato spotted wilt virus | 0.97 | 0.81 | -2.21 |
|  | HM015523 | 90 | Tomato spotted wilt virus | 0.97 | 0.81 | -2.01 |
|  | FM163370 | 90 | Tomato spotted wilt virus | 0.97 | 0.81 | -2.14 |
|  | FM163371 | 90 | Tomato spotted wilt virus | 0.97 | 0.81 | -2.14 |
|  | HM015524 | 90 | Tomato spotted wilt virus | 0.97 | 0.81 | -2.49 |
|  | HM015518 | 90 | Tomato spotted wilt virus | 0.97 | 0.81 | -3.49 |
|  | HM015522 | 90 | Tomato spotted wilt virus | 0.97 | 0.81 | -3.07 |
|  | HQ728386 | 109 | Soybean vein necrosis virus | -100 | -100 | null |
|  | FJ822961 | 110 | Calla lily chlorotic spot virus | 0.48 | 0.17 | -3.18 |
|  | FJ947152 | 110 | Tomato necrotic ringspot virus | 0.48 | 0.17 | -2.35 |
|  | NC_018072 | 110 | Bean necrotic mosaic virus | 0.48 | 0.17 | -8.27 |
|  | FJ361359 | 115 | Iris yellow spot virus | 0.41 | 0 | -4.44 |
|  | AF214014 | 115 | Iris yellow spot virus | 0.41 | 0 | -7.07 |
|  | NC_008307 | 115 | Melon yellow spot virus | 0.41 | 0 | 0.4 |
|  | JX833565 | 115 | Spider lily necrotic spot virus | 0.41 | 0 | -1.29 |
|  | NC_015468 | 115 | Groundnut ringspot and Tomato chlorotic spot virus reassortant | 0.41 | 0 | -2.45 |
|  | JN560177 | 115 | Tomato yellow ring virus | 0.41 | 0 | -2.13 |
|  | FJ011450 | 115 | Capsicum chlorosis virus | 0.41 | 0 | -1.04 |
|  | KC953854 | 115 | Capsicum chlorosis virus | 0.41 | 0 | -2.76 |
|  | NC_008303 | 115 | Capsicum chlorosis virus | 0.41 | 0 | -0.29 |
|  | GU474545 | 115 | Watermelon bud necrosis virus | 0.41 | 0 | -1.6 |
|  | GU584185 | 115 | Watermelon bud necrosis virus | 0.41 | 0 | -1.66 |
|  | NC_003620 | 115 | Groundnut bud necrosis virus | 0.41 | 0 | -3.36 |
|  | GQ336990 | 115 | Impatiens necrotic spot virus | 0.41 | 0 | -2.44 |
|  | GU112503 | 115 | Impatiens necrotic spot virus | 0.41 | 0 | -2.79 |
|  | DQ425095 | 115 | Impatiens necrotic spot virus | 0.41 | 0 | -2.8 |
|  | NC_003616 | 115 | Impatiens necrotic spot virus | 0.41 | 0 | -2.34 |
|  | DQ157768 | 115 | Watermelon silver mottle virus | 0.41 | 0 | -1.35 |
|  | NC_003841 | 115 | Watermelon silver mottle virus | 0.41 | 0 | -0.52 |
|  | JX177646 | 115 | Watermelon silver mottle virus | 0.41 | 0 | -2.44 |
|  | NC_010490 | 152 | Tomato zonate spot virus | -100 | -100 | null |
| tospovirus - M_complement | AY744484 | 6 | Tomato spotted wilt virus | 0.91 | 0.59 | -3.2 |
|  | AY744483 | 6 | Tomato spotted wilt virus | 0.91 | 0.59 | -2.84 |
|  | AY744482 | 6 | Tomato spotted wilt virus | 0.91 | 0.59 | -3.48 |
|  | JN664253 | 6 | Tomato spotted wilt virus | 0.91 | 0.59 | -3.4 |
|  | KC261975 | 6 | Tomato spotted wilt virus | 0.91 | 0.59 | -4.62 |
|  | AY744481 | 6 | Tomato spotted wilt virus | 0.91 | 0.59 | -4.64 |
|  | HM581941 | 6 | Tomato spotted wilt virus | 0.91 | 0.59 | -4.42 |
|  | HM581938 | 6 | Tomato spotted wilt virus | 0.91 | 0.59 | -4.22 |
|  | AB190818 | 6 | Tomato spotted wilt virus | 0.91 | 0.59 | -3.94 |
|  | AY744485 | 6 | Tomato spotted wilt virus | 0.91 | 0.59 | -5.17 |
|  | AY744491 | 6 | Tomato spotted wilt virus | 0.91 | 0.59 | -3.18 |
|  | AY870389 | 6 | Tomato spotted wilt virus | 0.91 | 0.59 | -3.42 |
|  | AY744490 | 6 | Tomato spotted wilt virus | 0.91 | 0.59 | -3.12 |
|  | AY744489 | 6 | Tomato spotted wilt virus | 0.91 | 0.59 | -2.49 |
|  | AY744488 | 6 | Tomato spotted wilt virus | 0.91 | 0.59 | -2.63 |
|  | AY744487 | 6 | Tomato spotted wilt virus | 0.91 | 0.59 | -2.68 |
|  | AY870390 | 6 | Tomato spotted wilt virus | 0.91 | 0.59 | -2.88 |
|  | JF960236 | 6 | Tomato spotted wilt virus | 0.91 | 0.59 | -4.37 |
|  | FM163373 | 6 | Tomato spotted wilt virus | 0.91 | 0.59 | -4.25 |
|  | FM163372 | 6 | Tomato spotted wilt virus | 0.91 | 0.59 | -4.33 |
|  | KC261951 | 49 | Tomato spotted wilt virus | 0.99 | 0.9 | -3.89 |
|  | KC261969 | 49 | Tomato spotted wilt virus | 0.99 | 0.9 | -4.46 |
|  | KC261960 | 49 | Tomato spotted wilt virus | 0.99 | 0.9 | -3.71 |
|  | KC261963 | 49 | Tomato spotted wilt virus | 0.99 | 0.9 | -4.84 |
|  | KC261957 | 56 | Tomato spotted wilt virus | 0.98 | 0.91 | -3.64 |
|  | KC261954 | 56 | Tomato spotted wilt virus | 0.98 | 0.91 | -3.25 |
|  | HM581935 | 56 | Tomato spotted wilt virus | 0.98 | 0.91 | -3.5 |
|  | HM015510 | 56 | Tomato spotted wilt virus | 0.98 | 0.91 | -4.26 |
|  | KC261948 | 63 | Tomato spotted wilt virus | -100 | -100 | null |
|  | HM015514 | 64 | Tomato spotted wilt virus | 0.96 | 0.92 | -3.62 |
|  | HM015524 | 64 | Tomato spotted wilt virus | 0.96 | 0.92 | -3.65 |
|  | HM015513 | 64 | Tomato spotted wilt virus | 0.96 | 0.92 | -4.49 |
|  | FM163371 | 64 | Tomato spotted wilt virus | 0.96 | 0.92 | -3.82 |
|  | HM015523 | 64 | Tomato spotted wilt virus | 0.96 | 0.92 | -3.16 |
|  | FM163370 | 64 | Tomato spotted wilt virus | 0.96 | 0.92 | -3.78 |
|  | HM015522 | 64 | Tomato spotted wilt virus | 0.96 | 0.92 | -3.66 |
|  | HM015518 | 64 | Tomato spotted wilt virus | 0.96 | 0.92 | -3.82 |
|  | HQ537114 | 64 | Tomato spotted wilt virus | 0.96 | 0.92 | -3.13 |
|  | AY744492 | 64 | Tomato spotted wilt virus | 0.96 | 0.92 | -3.83 |
|  | AY744493 | 83 | Tomato spotted wilt virus | -100 | -100 | null |
|  | HM015521 | 84 | Tomato spotted wilt virus | 0.96 | 0.7 | -3.37 |
|  | HQ830188 | 84 | Tomato spotted wilt virus | 0.96 | 0.7 | -4.08 |
|  | HM015520 | 84 | Tomato spotted wilt virus | 0.96 | 0.7 | -3.81 |
|  | HM015515 | 84 | Tomato spotted wilt virus | 0.96 | 0.7 | -3.69 |
|  | AY744486 | 84 | Tomato spotted wilt virus | 0.96 | 0.7 | -3.99 |
|  | KC261972 | 84 | Tomato spotted wilt virus | 0.96 | 0.7 | -4.67 |
|  | KC261966 | 84 | Tomato spotted wilt virus | 0.96 | 0.7 | -3.81 |
|  | HM015519 | 84 | Tomato spotted wilt virus | 0.96 | 0.7 | -4.41 |
|  | HM015517 | 84 | Tomato spotted wilt virus | 0.96 | 0.7 | -4.31 |
|  | HM015511 | 84 | Tomato spotted wilt virus | 0.96 | 0.7 | -3.64 |
|  | HM015512 | 84 | Tomato spotted wilt virus | 0.96 | 0.7 | -5.54 |
|  | HM015516 | 84 | Tomato spotted wilt virus | 0.96 | 0.7 | -3.22 |
|  | NC_002050 | 84 | Tomato spotted wilt virus | 0.96 | 0.7 | -2.3 |
|  | HQ728386 | 109 | Soybean vein necrosis virus | -100 | -100 | null |
|  | NC_018072 | 110 | Bean necrotic mosaic virus | -100 | -100 | null |
|  | JX833565 | 111 | Spider lily necrotic spot virus | 0.43 | 0.1 | -2.94 |
|  | NC_015468 | 111 | Groundnut ringspot and Tomato chlorotic spot virus reassortant | 0.43 | 0.1 | -1.9 |
|  | FJ947152 | 111 | Tomato necrotic ringspot virus | 0.43 | 0.1 | -2.13 |
|  | FJ822961 | 111 | Calla lily chlorotic spot virus | 0.43 | 0.1 | -3.16 |
|  | JN560177 | 111 | Tomato yellow ring virus | 0.43 | 0.1 | -1.8 |
|  | NC_003620 | 122 | Groundnut bud necrosis virus | 0.35 | 0 | -2.32 |
|  | GU474545 | 122 | Watermelon bud necrosis virus | 0.35 | 0 | -1.38 |
|  | GU584185 | 122 | Watermelon bud necrosis virus | 0.35 | 0 | -2.8 |
|  | KC953854 | 122 | Capsicum chlorosis virus | 0.35 | 0 | -4.31 |
|  | FJ011450 | 122 | Capsicum chlorosis virus | 0.35 | 0 | -1.63 |
|  | NC_008303 | 122 | Capsicum chlorosis virus | 0.35 | 0 | -0.45 |
|  | FJ361359 | 122 | Iris yellow spot virus | 0.35 | 0 | -4.12 |
|  | AF214014 | 122 | Iris yellow spot virus | 0.35 | 0 | -8.29 |
|  | NC_008307 | 122 | Melon yellow spot virus | 0.35 | 0 | -3.55 |
|  | NC_003616 | 140 | Impatiens necrotic spot virus | 0.95 | 0.76 | -3.87 |
|  | DQ425095 | 140 | Impatiens necrotic spot virus | 0.95 | 0.76 | -3.94 |
|  | GU112503 | 140 | Impatiens necrotic spot virus | 0.95 | 0.76 | -2.66 |
|  | GQ336990 | 140 | Impatiens necrotic spot virus | 0.95 | 0.76 | -3.05 |
|  | NC_003841 | 147 | Watermelon silver mottle virus | 0.83 | 0.57 | -2.4 |
|  | DQ157768 | 147 | Watermelon silver mottle virus | 0.83 | 0.57 | -3.39 |
|  | JX177646 | 147 | Watermelon silver mottle virus | 0.83 | 0.57 | -1.28 |
|  | NC_010490 | 152 | Tomato zonate spot virus | -100 | -100 | null |
| tenuivirus - 2 | D13787 | 4 | Rice stripe virus | 0.96 | 0.78 | -0.33 |
|  | D13176 | 4 | Rice stripe virus | 0.96 | 0.78 | 0.47 |
|  | EU931511 | 4 | Rice stripe virus | 0.96 | 0.78 | -0.66 |
|  | EU931515 | 4 | Rice stripe virus | 0.96 | 0.78 | -0.23 |
|  | AY186789 | 4 | Rice stripe virus | 0.96 | 0.78 | -0.62 |
|  | JQ927428 | 4 | Rice stripe virus | 0.96 | 0.78 | -0.78 |
|  | GQ229105 | 4 | Rice stripe virus | 0.96 | 0.78 | -0.34 |
|  | JQ927430 | 4 | Rice stripe virus | 0.96 | 0.78 | -0.29 |
|  | GQ229109 | 4 | Rice stripe virus | 0.96 | 0.78 | -0.87 |
|  | EU931523 | 4 | Rice stripe virus | 0.96 | 0.78 | -0.09 |
|  | GQ229100 | 4 | Rice stripe virus | 0.96 | 0.78 | -0.91 |
|  | GQ229110 | 4 | Rice stripe virus | 0.96 | 0.78 | -0.33 |
|  | JQ927427 | 4 | Rice stripe virus | 0.96 | 0.78 | -0.28 |
|  | GQ229099 | 4 | Rice stripe virus | 0.96 | 0.78 | -0.56 |
|  | GQ229111 | 4 | Rice stripe virus | 0.96 | 0.78 | -0.92 |
|  | GQ229102 | 4 | Rice stripe virus | 0.96 | 0.78 | -0.72 |
|  | GQ229107 | 4 | Rice stripe virus | 0.96 | 0.78 | -0.21 |
|  | EU931519 | 4 | Rice stripe virus | 0.96 | 0.78 | -0.95 |
|  | GQ229103 | 4 | Rice stripe virus | 0.96 | 0.78 | -0.13 |
|  | DQ333943 | 4 | Rice stripe virus | 0.96 | 0.78 | -2.28 |
|  | GQ229106 | 4 | Rice stripe virus | 0.96 | 0.78 | -1.07 |
|  | GQ229101 | 4 | Rice stripe virus | 0.96 | 0.78 | -0.5 |
|  | EF141328 | 4 | Rice stripe virus | 0.96 | 0.78 | -1.2 |
|  | GQ229104 | 4 | Rice stripe virus | 0.96 | 0.78 | -0.24 |
|  | EU931495 | 51 | Rice stripe virus | 0.93 | 0.77 | -0.25 |
|  | EU931499 | 51 | Rice stripe virus | 0.93 | 0.77 | -0.77 |
|  | JQ927429 | 51 | Rice stripe virus | 0.93 | 0.77 | 0.16 |
|  | JQ927426 | 51 | Rice stripe virus | 0.93 | 0.77 | -1.14 |
|  | EU931503 | 51 | Rice stripe virus | 0.93 | 0.77 | -1.9 |
|  | EU931507 | 51 | Rice stripe virus | 0.93 | 0.77 | -1.31 |
|  | AY312434 | 62 | Iranian wheat stripe virus | -100 | -100 | null |
|  | U53224 | 63 | Maize stripe virus | -100 | -100 | null |
|  | AF511072 | 64 | Rice grassy stunt virus | 0.93 | 0.83 | -0.26 |
|  | NC_002324 | 64 | Rice grassy stunt virus | 0.93 | 0.83 | -0.19 |
|  | AB023777 | 64 | Rice grassy stunt virus | 0.93 | 0.83 | 0.82 |
| tenuivirus - 2_complement | EU931515 | 0 | Rice stripe virus | 0.93 | 0.25 | -1.24 |
|  | GQ229100 | 0 | Rice stripe virus | 0.93 | 0.25 | -1.86 |
|  | GQ229102 | 0 | Rice stripe virus | 0.93 | 0.25 | -2 |
|  | JQ927430 | 0 | Rice stripe virus | 0.93 | 0.25 | -1.77 |
|  | GQ229110 | 0 | Rice stripe virus | 0.93 | 0.25 | -1.6 |
|  | JQ927428 | 0 | Rice stripe virus | 0.93 | 0.25 | -0.51 |
|  | EU931511 | 0 | Rice stripe virus | 0.93 | 0.25 | -1.32 |
|  | JQ927427 | 0 | Rice stripe virus | 0.93 | 0.25 | -1.25 |
|  | AY186789 | 0 | Rice stripe virus | 0.93 | 0.25 | -1.48 |
|  | GQ229105 | 0 | Rice stripe virus | 0.93 | 0.25 | -0.69 |
|  | GQ229099 | 0 | Rice stripe virus | 0.93 | 0.25 | -0.91 |
|  | DQ333943 | 0 | Rice stripe virus | 0.93 | 0.25 | -1.81 |
|  | GQ229111 | 0 | Rice stripe virus | 0.93 | 0.25 | -2.11 |
|  | GQ229103 | 0 | Rice stripe virus | 0.93 | 0.25 | -0.54 |
|  | GQ229109 | 0 | Rice stripe virus | 0.93 | 0.25 | -0.38 |
|  | GQ229107 | 0 | Rice stripe virus | 0.93 | 0.25 | -0.41 |
|  | EU931519 | 0 | Rice stripe virus | 0.93 | 0.25 | -1.31 |
|  | EU931523 | 0 | Rice stripe virus | 0.93 | 0.25 | -0.75 |
|  | D13176 | 0 | Rice stripe virus | 0.93 | 0.25 | -2.37 |
|  | GQ229104 | 0 | Rice stripe virus | 0.93 | 0.25 | -1.22 |
|  | GQ229101 | 0 | Rice stripe virus | 0.93 | 0.25 | -0.44 |
|  | EF141328 | 0 | Rice stripe virus | 0.93 | 0.25 | -1.03 |
|  | D13787 | 0 | Rice stripe virus | 0.93 | 0.25 | -2.14 |
|  | GQ229106 | 0 | Rice stripe virus | 0.93 | 0.25 | -0.46 |
|  | EU931495 | 0 | Rice stripe virus | 0.93 | 0.25 | -0.18 |
|  | EU931499 | 0 | Rice stripe virus | 0.93 | 0.25 | 0.1 |
|  | JQ927429 | 0 | Rice stripe virus | 0.93 | 0.25 | 1.05 |
|  | JQ927426 | 0 | Rice stripe virus | 0.93 | 0.25 | 0.1 |
|  | EU931503 | 0 | Rice stripe virus | 0.93 | 0.25 | -1.48 |
|  | EU931507 | 0 | Rice stripe virus | 0.93 | 0.25 | 0.01 |
|  | U53224 | 0 | Maize stripe virus | 0.93 | 0.25 | -3.56 |
|  | AY312434 | 0 | Iranian wheat stripe virus | 0.93 | 0.25 | -1.02 |
|  | NC_002324 | 0 | Rice grassy stunt virus | 0.93 | 0.25 | -0.08 |
|  | AF511072 | 0 | Rice grassy stunt virus | 0.93 | 0.25 | 0.01 |
|  | AB023777 | 0 | Rice grassy stunt virus | 0.93 | 0.25 | -0.53 |
| tenuivirus - 4_cpu | AF221834 | 0 | Rice stripe virus | 0.81 | 0.04 | -3.32 |
|  | AF221830 | 0 | Rice stripe virus | 0.81 | 0.04 | -4.12 |
|  | EU931525 | 0 | Rice stripe virus | 0.81 | 0.04 | -3.58 |
|  | FJ602688 | 0 | Rice stripe virus | 0.81 | 0.04 | -3.25 |
|  | FJ602696 | 0 | Rice stripe virus | 0.81 | 0.04 | -4.01 |
|  | AF221835 | 0 | Rice stripe virus | 0.81 | 0.04 | -3.06 |
|  | EF141330 | 0 | Rice stripe virus | 0.81 | 0.04 | -3.09 |
|  | FJ602697 | 0 | Rice stripe virus | 0.81 | 0.04 | -3.82 |
|  | FJ602699 | 0 | Rice stripe virus | 0.81 | 0.04 | -3.77 |
|  | NC_003753 | 0 | Rice stripe virus | 0.81 | 0.04 | -3.02 |
|  | JQ927415 | 0 | Rice stripe virus | 0.81 | 0.04 | -1.86 |
|  | EU931521 | 0 | Rice stripe virus | 0.81 | 0.04 | -3.59 |
|  | EF538684 | 0 | Rice stripe virus | 0.81 | 0.04 | -1.86 |
|  | FJ602692 | 0 | Rice stripe virus | 0.81 | 0.04 | -3.53 |
|  | FJ602689 | 0 | Rice stripe virus | 0.81 | 0.04 | -3.66 |
|  | DQ333945 | 0 | Rice stripe virus | 0.81 | 0.04 | -4.41 |
|  | FJ602700 | 0 | Rice stripe virus | 0.81 | 0.04 | -3.47 |
|  | AF513505 | 0 | Rice stripe virus | 0.81 | 0.04 | -2.41 |
|  | EU931517 | 0 | Rice stripe virus | 0.81 | 0.04 | -3.81 |
|  | AY185501 | 0 | Rice stripe virus | 0.81 | 0.04 | -3.89 |
|  | EU931513 | 0 | Rice stripe virus | 0.81 | 0.04 | -4.21 |
|  | FJ602694 | 0 | Rice stripe virus | 0.81 | 0.04 | -4.4 |
|  | JQ927416 | 0 | Rice stripe virus | 0.81 | 0.04 | -4.36 |
|  | AY185502 | 0 | Rice stripe virus | 0.81 | 0.04 | -3.75 |
|  | FJ602691 | 0 | Rice stripe virus | 0.81 | 0.04 | -3.92 |
|  | FJ602698 | 0 | Rice stripe virus | 0.81 | 0.04 | -3.16 |
|  | JQ927418 | 0 | Rice stripe virus | 0.81 | 0.04 | -4.99 |
|  | D01039 | 0 | Rice stripe virus | 0.81 | 0.04 | -2.87 |
|  | FJ602690 | 0 | Rice stripe virus | 0.81 | 0.04 | -4.06 |
|  | FJ602695 | 0 | Rice stripe virus | 0.81 | 0.04 | -3.38 |
|  | AJ875055 | 0 | Rice stripe virus | 0.81 | 0.04 | -2.96 |
|  | AJ871750 | 0 | Rice stripe virus | 0.81 | 0.04 | -3.53 |
|  | AJ871751 | 0 | Rice stripe virus | 0.81 | 0.04 | -4.02 |
|  | AJ871753 | 0 | Rice stripe virus | 0.81 | 0.04 | -3.96 |
|  | AJ871749 | 0 | Rice stripe virus | 0.81 | 0.04 | -2 |
|  | JQ927417 | 0 | Rice stripe virus | 0.81 | 0.04 | -1.92 |
|  | AJ871755 | 0 | Rice stripe virus | 0.81 | 0.04 | -2.37 |
|  | JQ927414 | 0 | Rice stripe virus | 0.81 | 0.04 | -4.08 |
|  | EU931501 | 0 | Rice stripe virus | 0.81 | 0.04 | -2.44 |
|  | AJ871752 | 0 | Rice stripe virus | 0.81 | 0.04 | -2.72 |
|  | AJ875056 | 0 | Rice stripe virus | 0.81 | 0.04 | -3.09 |
|  | EU931497 | 0 | Rice stripe virus | 0.81 | 0.04 | -3.29 |
|  | AJ871748 | 0 | Rice stripe virus | 0.81 | 0.04 | -4.3 |
|  | AY185499 | 0 | Rice stripe virus | 0.81 | 0.04 | -4.92 |
|  | EU931505 | 0 | Rice stripe virus | 0.81 | 0.04 | -5.1 |
|  | AJ871754 | 0 | Rice stripe virus | 0.81 | 0.04 | -3.99 |
|  | AY185500 | 0 | Rice stripe virus | 0.81 | 0.04 | -4.28 |
|  | EU931509 | 0 | Rice stripe virus | 0.81 | 0.04 | -2.73 |
|  | JQ927419 | 0 | Rice stripe virus | 0.81 | 0.04 | -3.62 |
|  | NC_002326 | 0 | Rice grassy stunt virus | 0.81 | 0.04 | -0.77 |
|  | AF290946 | 0 | Rice grassy stunt virus | 0.81 | 0.04 | -1.26 |
|  | AB023778 | 0 | Rice grassy stunt virus | 0.81 | 0.04 | -1.17 |
|  | AY312436 | 0 | Iranian wheat stripe virus | 0.81 | 0.04 | -2.18 |
| tenuivirus - 4_complement | FJ602697 | 0 | Rice stripe virus | 0.87 | 0.16 | -3.52 |
|  | FJ602699 | 0 | Rice stripe virus | 0.87 | 0.16 | -3.43 |
|  | AF221835 | 0 | Rice stripe virus | 0.87 | 0.16 | -2.94 |
|  | EU931525 | 0 | Rice stripe virus | 0.87 | 0.16 | -1.54 |
|  | FJ602696 | 0 | Rice stripe virus | 0.87 | 0.16 | -2.61 |
|  | FJ602688 | 0 | Rice stripe virus | 0.87 | 0.16 | -2.02 |
|  | EF141330 | 0 | Rice stripe virus | 0.87 | 0.16 | -2.66 |
|  | AF221830 | 0 | Rice stripe virus | 0.87 | 0.16 | -2.79 |
|  | AF221834 | 0 | Rice stripe virus | 0.87 | 0.16 | -2.6 |
|  | JQ927415 | 0 | Rice stripe virus | 0.87 | 0.16 | -2.22 |
|  | NC_003753 | 0 | Rice stripe virus | 0.87 | 0.16 | -3.91 |
|  | EU931521 | 0 | Rice stripe virus | 0.87 | 0.16 | -1.96 |
|  | EF538684 | 0 | Rice stripe virus | 0.87 | 0.16 | -2.26 |
|  | FJ602689 | 0 | Rice stripe virus | 0.87 | 0.16 | -3.76 |
|  | FJ602692 | 0 | Rice stripe virus | 0.87 | 0.16 | -3.24 |
|  | DQ333945 | 0 | Rice stripe virus | 0.87 | 0.16 | -2.72 |
|  | FJ602700 | 0 | Rice stripe virus | 0.87 | 0.16 | -2.74 |
|  | EU931517 | 0 | Rice stripe virus | 0.87 | 0.16 | -2.83 |
|  | AF513505 | 0 | Rice stripe virus | 0.87 | 0.16 | -3.2 |
|  | JQ927418 | 0 | Rice stripe virus | 0.87 | 0.16 | -2.94 |
|  | JQ927416 | 0 | Rice stripe virus | 0.87 | 0.16 | -1.6 |
|  | FJ602694 | 0 | Rice stripe virus | 0.87 | 0.16 | -2.78 |
|  | EU931513 | 0 | Rice stripe virus | 0.87 | 0.16 | -2.67 |
|  | AY185501 | 0 | Rice stripe virus | 0.87 | 0.16 | -1.43 |
|  | FJ602691 | 0 | Rice stripe virus | 0.87 | 0.16 | -2.1 |
|  | AY185502 | 0 | Rice stripe virus | 0.87 | 0.16 | -2.48 |
|  | FJ602698 | 0 | Rice stripe virus | 0.87 | 0.16 | -2.03 |
|  | FJ602690 | 0 | Rice stripe virus | 0.87 | 0.16 | -2.47 |
|  | FJ602695 | 0 | Rice stripe virus | 0.87 | 0.16 | -2.2 |
|  | D01039 | 0 | Rice stripe virus | 0.87 | 0.16 | -0.94 |
|  | JQ927414 | 0 | Rice stripe virus | 0.87 | 0.16 | -3.15 |
|  | EU931501 | 0 | Rice stripe virus | 0.87 | 0.16 | -2.63 |
|  | AJ871755 | 0 | Rice stripe virus | 0.87 | 0.16 | -2.36 |
|  | JQ927417 | 0 | Rice stripe virus | 0.87 | 0.16 | -2.61 |
|  | AJ871749 | 0 | Rice stripe virus | 0.87 | 0.16 | -2.03 |
|  | AJ871752 | 0 | Rice stripe virus | 0.87 | 0.16 | -1.93 |
|  | AJ871751 | 0 | Rice stripe virus | 0.87 | 0.16 | -4.05 |
|  | AJ871750 | 0 | Rice stripe virus | 0.87 | 0.16 | -3.86 |
|  | AJ875055 | 0 | Rice stripe virus | 0.87 | 0.16 | -2.9 |
|  | AJ871753 | 0 | Rice stripe virus | 0.87 | 0.16 | -2.53 |
|  | AJ875056 | 0 | Rice stripe virus | 0.87 | 0.16 | -3.7 |
|  | AY185499 | 0 | Rice stripe virus | 0.87 | 0.16 | -4.22 |
|  | AJ871748 | 0 | Rice stripe virus | 0.87 | 0.16 | -5.38 |
|  | EU931505 | 0 | Rice stripe virus | 0.87 | 0.16 | -4.5 |
|  | EU931497 | 0 | Rice stripe virus | 0.87 | 0.16 | -3.25 |
|  | EU931509 | 0 | Rice stripe virus | 0.87 | 0.16 | -4.79 |
|  | AY185500 | 0 | Rice stripe virus | 0.87 | 0.16 | -4.39 |
|  | AJ871754 | 0 | Rice stripe virus | 0.87 | 0.16 | -4.15 |
|  | JQ927419 | 0 | Rice stripe virus | 0.87 | 0.16 | -2.82 |
|  | AF290946 | 0 | Rice grassy stunt virus | 0.87 | 0.16 | -2.68 |
|  | NC_002326 | 0 | Rice grassy stunt virus | 0.87 | 0.16 | -3.37 |
|  | AB023778 | 0 | Rice grassy stunt virus | 0.87 | 0.16 | -2.72 |
|  | AY312436 | 0 | Iranian wheat stripe virus | 0.87 | 0.16 | -1.63 |
| phlebovirus - S | HE687307 | 6 | Rift Valley fever virus | 0.93 | 0.93 | -3.16 |
|  | JF326200 | 6 | Rift Valley fever virus | 0.93 | 0.93 | -1.34 |
|  | EU709747 | 6 | Rift Valley fever virus | 0.93 | 0.93 | -1.23 |
|  | EU574087 | 6 | Rift Valley fever virus | 0.93 | 0.93 | -1.41 |
|  | EU574066 | 6 | Rift Valley fever virus | 0.93 | 0.93 | -1.4 |
|  | JF311388 | 6 | Rift Valley fever virus | 0.93 | 0.93 | -1.44 |
|  | JQ840745 | 6 | Rift Valley fever virus | 0.93 | 0.93 | -0.13 |
|  | JF311391 | 6 | Rift Valley fever virus | 0.93 | 0.93 | -1.4 |
|  | EU312131 | 6 | Rift Valley fever virus | 0.93 | 0.93 | -1.35 |
|  | EU312133 | 6 | Rift Valley fever virus | 0.93 | 0.93 | -0.23 |
|  | EU312107 | 6 | Rift Valley fever virus | 0.93 | 0.93 | -2.84 |
|  | EU312108 | 6 | Rift Valley fever virus | 0.93 | 0.93 | -2.89 |
|  | EU312119 | 6 | Rift Valley fever virus | 0.93 | 0.93 | -1.33 |
|  | EU312115 | 6 | Rift Valley fever virus | 0.93 | 0.93 | -1.42 |
|  | EU312128 | 6 | Rift Valley fever virus | 0.93 | 0.93 | -1.45 |
|  | EU312146 | 6 | Rift Valley fever virus | 0.93 | 0.93 | -1.36 |
|  | EU312141 | 6 | Rift Valley fever virus | 0.93 | 0.93 | -0.53 |
|  | JQ820481 | 6 | Rift Valley fever virus | 0.93 | 0.93 | -0.08 |
|  | JQ840746 | 6 | Rift Valley fever virus | 0.93 | 0.93 | -0.24 |
|  | EU312139 | 6 | Rift Valley fever virus | 0.93 | 0.93 | -0.71 |
|  | EU574086 | 6 | Rift Valley fever virus | 0.93 | 0.93 | -0.16 |
|  | EU574057 | 6 | Rift Valley fever virus | 0.93 | 0.93 | -0.08 |
|  | DQ380154 | 6 | Rift Valley fever virus | 0.93 | 0.93 | -1.28 |
|  | EU312124 | 6 | Rift Valley fever virus | 0.93 | 0.93 | -1.32 |
|  | NC_014395 | 6 | Rift Valley fever virus | 0.93 | 0.93 | -1.29 |
|  | EU312114 | 6 | Rift Valley fever virus | 0.93 | 0.93 | -1.25 |
|  | DQ380152 | 6 | Rift Valley fever virus | 0.93 | 0.93 | -0.77 |
|  | DQ380146 | 6 | Rift Valley fever virus | 0.93 | 0.93 | -2.82 |
|  | DQ380161 | 6 | Rift Valley fever virus | 0.93 | 0.93 | -1.29 |
|  | EU312122 | 6 | Rift Valley fever virus | 0.93 | 0.93 | -1.58 |
|  | DQ380163 | 6 | Rift Valley fever virus | 0.93 | 0.93 | -1.37 |
|  | EU312135 | 6 | Rift Valley fever virus | 0.93 | 0.93 | -1.43 |
|  | DQ380172 | 6 | Rift Valley fever virus | 0.93 | 0.93 | -1.32 |
|  | EU312109 | 6 | Rift Valley fever virus | 0.93 | 0.93 | -1.35 |
|  | EU312136 | 6 | Rift Valley fever virus | 0.93 | 0.93 | -1.67 |
|  | EU312123 | 6 | Rift Valley fever virus | 0.93 | 0.93 | -1.32 |
|  | DQ380179 | 6 | Rift Valley fever virus | 0.93 | 0.93 | -1.02 |
|  | DQ380155 | 6 | Rift Valley fever virus | 0.93 | 0.93 | -1.06 |
|  | DQ380159 | 6 | Rift Valley fever virus | 0.93 | 0.93 | -1.21 |
|  | EU312118 | 6 | Rift Valley fever virus | 0.93 | 0.93 | -1.44 |
|  | JQ068142 | 6 | Rift Valley fever virus | 0.93 | 0.93 | -1.47 |
|  | EU312111 | 6 | Rift Valley fever virus | 0.93 | 0.93 | 0.09 |
|  | DQ380181 | 6 | Rift Valley fever virus | 0.93 | 0.93 | -1.5 |
|  | EU312117 | 6 | Rift Valley fever virus | 0.93 | 0.93 | -1.47 |
|  | DQ380173 | 6 | Rift Valley fever virus | 0.93 | 0.93 | -1.22 |
|  | DQ380156 | 6 | Rift Valley fever virus | 0.93 | 0.93 | -1.4 |
|  | EU312112 | 6 | Rift Valley fever virus | 0.93 | 0.93 | -1.36 |
|  | EU312132 | 6 | Rift Valley fever virus | 0.93 | 0.93 | -1.28 |
|  | DQ380157 | 6 | Rift Valley fever virus | 0.93 | 0.93 | -0.02 |
|  | EU312104 | 6 | Rift Valley fever virus | 0.93 | 0.93 | -1.01 |
|  | EU312116 | 6 | Rift Valley fever virus | 0.93 | 0.93 | -1.01 |
|  | EU312121 | 6 | Rift Valley fever virus | 0.93 | 0.93 | -0.89 |
|  | EU312103 | 6 | Rift Valley fever virus | 0.93 | 0.93 | -2.98 |
|  | EU312130 | 6 | Rift Valley fever virus | 0.93 | 0.93 | -3.02 |
|  | JF784388 | 6 | Rift Valley fever virus | 0.93 | 0.93 | -2.81 |
|  | EF201821 | 6 | Corfou virus | 0.93 | 0.93 | -3.41 |
|  | HM566175 | 6 | Odrenisrou virus | 0.93 | 0.93 | -0.87 |
|  | HM627184 | 6 | Salobo virus | 0.93 | 0.93 | -0.7 |
|  | JF838326 | 122 | Chize virus | -100 | -100 | null |
|  | JF838329 | 125 | Grand Arbaud virus | -100 | -100 | null |
|  | JF838332 | 127 | Murre virus | 0.52 | 0.95 | -1.49 |
|  | HM566160 | 127 | EgAN 1825-61 virus | 0.52 | 0.95 | 0.12 |
|  | KF767465 | 130 | Zaliv Terpenia virus | 0.92 | 0.85 | -0.31 |
|  | KF767462 | 130 | Zaliv Terpenia virus | 0.92 | 0.85 | -0.31 |
|  | HM566192 | 130 | Zaliv Terpenia virus | 0.92 | 0.85 | -1.02 |
|  | JX005842 | 135 | Heartland virus | 0.73 | 0.75 | -1.03 |
|  | JX005843 | 135 | Heartland virus | 0.73 | 0.75 | -1.39 |
|  | HM119403 | 138 | Alenquer virus | 0.41 | 0.58 | -0.81 |
|  | HM119427 | 138 | Nique virus | 0.41 | 0.58 | -1.14 |
|  | FJ153285 | 146 | Toscana virus | 0.92 | 0.67 | 1.75 |
|  | EF201833 | 146 | Toscana virus | 0.92 | 0.67 | 1.96 |
|  | FJ153286 | 146 | Toscana virus | 0.92 | 0.67 | 1.72 |
|  | EU327772 | 151 | Toscana virus | 0.92 | 0.25 | 1.61 |
|  | JX867536 | 151 | Toscana virus | 0.92 | 0.25 | 1.4 |
|  | NC_006318 | 151 | Sandfly fever Naples virus | 0.92 | 0.25 | 1.63 |
|  | EF201828 | 156 | Sandfly fever Naples virus | 0.88 | 0.21 | -0.2 |
|  | EF201832 | 156 | Sandfly fever Naples virus | 0.88 | 0.21 | 1.13 |
|  | HM566182 | 156 | Sand fever Naples-like virus | 0.88 | 0.21 | 1.22 |
|  | HM566170 | 156 | Sandfly fever Naples virus | 0.88 | 0.21 | 0.11 |
|  | EF201824 | 163 | Sandfly fever Sicilian virus | -100 | -100 | null |
|  | AB817996 | 165 | Severe fever with thrombocytopenia syndrome virus | 0.94 | 0.92 | -1.27 |
|  | AB818002 | 165 | Severe fever with thrombocytopenia syndrome virus | 0.94 | 0.92 | -1.57 |
|  | KC292295 | 165 | SFTS virus HNXY_278 | 0.94 | 0.92 | -1.27 |
|  | HQ830171 | 165 | Phlebovirus JS6 | 0.94 | 0.92 | 0.55 |
|  | KC292274 | 165 | SFTS virus HNXY_188 | 0.94 | 0.92 | 0.71 |
|  | HQ419244 | 165 | Huaiyangshan virus | 0.94 | 0.92 | 0.71 |
|  | HQ419242 | 165 | Huaiyangshan virus | 0.94 | 0.92 | 0.44 |
|  | JQ693001 | 165 | Severe fever with thrombocytopenia syndrome virus | 0.94 | 0.92 | -0.18 |
|  | JQ684873 | 165 | Severe fever with thrombocytopenia syndrome virus | 0.94 | 0.92 | 0.44 |
|  | KF887434 | 165 | Severe fever with thrombocytopenia syndrome virus | 0.94 | 0.92 | 0.98 |
|  | KC292283 | 165 | SFTS virus HNXY_31 | 0.94 | 0.92 | 0.35 |
|  | KC292281 | 165 | SFTS virus HNXY_186 | 0.94 | 0.92 | 1.11 |
|  | KC292298 | 165 | SFTS virus HNXY_202 | 0.94 | 0.92 | 0.57 |
|  | NC_018137 | 165 | SFTS virus HB29 | 0.94 | 0.92 | 0.49 |
|  | KC292279 | 165 | SFTS virus HNXY_174 | 0.94 | 0.92 | -1.25 |
|  | JQ693004 | 165 | Severe fever with thrombocytopenia syndrome virus | 0.94 | 0.92 | -0.58 |
|  | AB817997 | 165 | Severe fever with thrombocytopenia syndrome virus | 0.94 | 0.92 | 0.07 |
|  | KF374683 | 165 | Severe fever with thrombocytopenia syndrome virus | 0.94 | 0.92 | 0.07 |
|  | AB817999 | 200 | Severe fever with thrombocytopenia syndrome virus | 0.96 | 0.75 | 0.69 |
|  | HM745932 | 200 | SFTS virus HB29 | 0.96 | 0.75 | 0.75 |
|  | HQ419239 | 200 | Huaiyangshan virus | 0.96 | 0.75 | 0.81 |
|  | HQ419243 | 200 | Huaiyangshan virus | 0.96 | 0.75 | 0.78 |
|  | EU725773 | 207 | Massilia virus | -100 | -100 | null |
|  | EF201826 | 208 | Sandfly fever Sicilian virus | -100 | -100 | null |
|  | HM466936 | 214 | Jacunda virus | 0.76 | 0.6 | -0.53 |
|  | HM119424 | 214 | Morumbi virus | 0.76 | 0.6 | -0.29 |
|  | HM119421 | 214 | Mucura virus | 0.76 | 0.6 | 0.61 |
|  | HM119430 | 214 | Serra Norte virus | 0.76 | 0.6 | 0.33 |
|  | HM119406 | 221 | Ariquemes virus | 0.53 | 0.6 | -1.75 |
|  | HM119415 | 221 | Maldonado virus | 0.53 | 0.6 | -1.5 |
|  | HM119418 | 221 | Itaituba virus | 0.53 | 0.6 | -4.28 |
|  | EF201836 | 227 | Phlebovirus sp. Co Ar 171616 | 0.75 | 0.86 | -0.46 |
|  | HM566168 | 227 | Sandfly fever Naples virus | 0.75 | 0.86 | -0.38 |
|  | EF201839 | 230 | Buenaventura virus | 0.5 | 0.32 | 0.27 |
|  | HM566151 | 230 | Phlebovirus CoAr 170255 | 0.5 | 0.32 | -0.65 |
|  | KF297902 | 230 | Gordil virus | 0.5 | 0.32 | 1.66 |
|  | HM119436 | 235 | Oriximina virus | 0.23 | 0.08 | 0.83 |
|  | HM566148 | 235 | Chagres virus | 0.23 | 0.08 | -1.05 |
|  | GQ847511 | 235 | Sandfly fever Turkey virus | 0.23 | 0.08 | -1.31 |
|  | EF201817 | 240 | Phlebovirus sp. Be An 356637 | 0.42 | 0 | -0.46 |
|  | EF201822 | 240 | Sandfly fever Sicilian virus | 0.42 | 0 | -0.27 |
|  | EF201825 | 240 | Sandfly fever Sicilian virus | 0.42 | 0 | -2.15 |
|  | NC_015413 | 240 | Sandfly fever Turkey virus | 0.42 | 0 | -2.06 |
|  | EF201827 | 240 | Sandfly fever Sicilian virus | 0.42 | 0 | -1.08 |
|  | EF201823 | 240 | Sandfly fever Sicilian virus | 0.42 | 0 | -2.46 |
|  | KF297905 | 240 | Gabek Forest virus | 0.42 | 0 | 2.6 |
|  | HM566166 | 240 | Munguba virus | 0.42 | 0 | -0.85 |
|  | HM566156 | 240 | Durania virus | 0.42 | 0 | -1.7 |
|  | HM566161 | 240 | Ixcanal virus | 0.42 | 0 | -4.02 |
|  | HQ661807 | 240 | Armero virus | 0.42 | 0 | -0.81 |
|  | NC_015452 | 240 | Aguacate virus | 0.42 | 0 | -3.19 |
|  | NC_023634 | 240 | Arumowot virus | 0.42 | 0 | -1.84 |
|  | HM566153 | 240 | Phlebovirus CoAr 171616 | 0.42 | 0 | -1.19 |
|  | EF201819 | 240 | Phlebovirus sp. VP-161A | 0.42 | 0 | 0.13 |
|  | HM119433 | 271 | Turuna virus | 0.38 | 0.35 | -2.5 |
|  | HM119412 | 271 | Echarate virus | 0.38 | 0.35 | -0.29 |
|  | JQ956378 | 279 | Bhanja virus | 0.58 | 0.89 | -6.18 |
|  | NC_022632 | 279 | Razdan virus | 0.58 | 0.89 | -6.47 |
|  | JX961630 | 282 | Palma virus | 0.69 | 0.93 | -6.17 |
|  | JX961624 | 282 | Bhanja virus | 0.69 | 0.93 | -5.59 |
|  | KC521442 | 285 | Bhanja virus | -100 | -100 | null |
|  | JX961627 | 286 | Forecariah virus | 0.72 | 0.9 | -3.95 |
|  | JX961618 | 286 | Bhanja virus | 0.72 | 0.9 | -4.89 |
|  | JQ956381 | 289 | Palma virus | 0.72 | 0.85 | -6.21 |
|  | JX961621 | 289 | Bhanja virus | 0.72 | 0.85 | -5.83 |
|  | KC335498 | 289 | Razdan virus | 0.72 | 0.85 | -7.96 |
|  | HM566180 | 294 | Precarious point virus | -100 | -100 | null |
|  | EF201820 | 296 | Phlebovirus sp. Be Ar 371637 | 0.35 | 0.83 | -1.92 |
|  | KF297914 | 296 | Karimabad virus | 0.35 | 0.83 | -1.41 |
|  | EF201830 | 299 | Sandfly fever Naples virus | 0.74 | 0.96 | 0.24 |
|  | HM566178 | 299 | Sand fever Naples-like virus | 0.74 | 0.96 | 0.31 |
|  | DQ380150 | 306 | Rift Valley fever virus | 0.24 | 0.77 | 0.72 |
|  | EF201829 | 306 | Sandfly fever Naples virus | 0.24 | 0.77 | 1.48 |
|  | EF201815 | 309 | Phlebovirus sp. Be An 578142 | 0.7 | 0.85 | -2.35 |
|  | EF201816 | 309 | Phlebovirus sp. Be An 416992 | 0.7 | 0.85 | -1.5 |
|  | EF201838 | 312 | Phlebovirus sp. VP-366G | 0.27 | 0 | -1.12 |
|  | EF201842 | 312 | Phlebovirus sp. VP-334K | 0.27 | 0 | 1.23 |
|  | HM566190 | 312 | Uriurana virus | 0.27 | 0 | 0.05 |
|  | HM566187 | 312 | Phlebovirus GGP-2011a | 0.27 | 0 | 1.04 |
|  | EF201834 | 319 | Punta Toro virus | 0.64 | 0.46 | -1.44 |
|  | EF201843 | 319 | Phlebovirus sp. PAN 479603 | 0.64 | 0.46 | -1.41 |
|  | EF201844 | 319 | Phlebovirus sp. PAN 483391 | 0.64 | 0.46 | -1.19 |
|  | EF201835 | 319 | Punta Toro virus | 0.64 | 0.46 | -0.97 |
|  | EF201837 | 319 | Phlebovirus sp. GML 902878 | 0.64 | 0.46 | -2.48 |
|  | EF201841 | 319 | Phlebovirus sp. Pa Ar 2381 | 0.64 | 0.46 | -2.71 |
|  | DQ363406 | 319 | Punta Toro virus | 0.64 | 0.46 | -0.26 |
|  | EF201818 | 332 | Phlebovirus sp. Be An 24262 | 0.31 | 0.3 | -3.06 |
|  | EF201831 | 332 | Sandfly fever Naples virus | 0.31 | 0.3 | -0.35 |
| phlebovirus - S_complement | HQ830171 | 7 | Phlebovirus JS6 | 0.96 | 0.66 | 0.87 |
|  | HQ419244 | 7 | Huaiyangshan virus | 0.96 | 0.66 | 0.78 |
|  | KC292274 | 7 | SFTS virus HNXY_188 | 0.96 | 0.66 | 0.78 |
|  | KC292283 | 7 | SFTS virus HNXY_31 | 0.96 | 0.66 | 1.09 |
|  | JQ684873 | 7 | Severe fever with thrombocytopenia syndrome virus | 0.96 | 0.66 | 0.81 |
|  | HQ419242 | 7 | Huaiyangshan virus | 0.96 | 0.66 | 0.81 |
|  | AB817997 | 7 | Severe fever with thrombocytopenia syndrome virus | 0.96 | 0.66 | 0.16 |
|  | KF374683 | 7 | Severe fever with thrombocytopenia syndrome virus | 0.96 | 0.66 | 0.16 |
|  | KC292298 | 7 | SFTS virus HNXY_202 | 0.96 | 0.66 | 0.71 |
|  | NC_018137 | 7 | SFTS virus HB29 | 0.96 | 0.66 | 0.75 |
|  | JQ693004 | 7 | Severe fever with thrombocytopenia syndrome virus | 0.96 | 0.66 | 0.23 |
|  | KC292281 | 7 | SFTS virus HNXY_186 | 0.96 | 0.66 | 0.78 |
|  | KF887434 | 7 | Severe fever with thrombocytopenia syndrome virus | 0.96 | 0.66 | 0.75 |
|  | KC292279 | 7 | SFTS virus HNXY_174 | 0.96 | 0.66 | 1.27 |
|  | AB817996 | 7 | Severe fever with thrombocytopenia syndrome virus | 0.96 | 0.66 | 1.3 |
|  | AB818002 | 7 | Severe fever with thrombocytopenia syndrome virus | 0.96 | 0.66 | 0.69 |
|  | KC292295 | 7 | SFTS virus HNXY_278 | 0.96 | 0.66 | 0.65 |
|  | JQ693001 | 7 | Severe fever with thrombocytopenia syndrome virus | 0.96 | 0.66 | 0.81 |
|  | HQ419239 | 42 | Huaiyangshan virus | 0.95 | 0.9 | 0.44 |
|  | HQ419243 | 42 | Huaiyangshan virus | 0.95 | 0.9 | 0.71 |
|  | AB817999 | 42 | Severe fever with thrombocytopenia syndrome virus | 0.95 | 0.9 | -1.57 |
|  | HM745932 | 42 | SFTS virus HB29 | 0.95 | 0.9 | 0.49 |
|  | EU725773 | 49 | Massilia virus | -100 | -100 | null |
|  | FJ153286 | 53 | Toscana virus | 0.92 | 0.98 | 0.08 |
|  | FJ153285 | 53 | Toscana virus | 0.92 | 0.98 | -1.05 |
|  | EF201833 | 53 | Toscana virus | 0.92 | 0.98 | 0.26 |
|  | EU327772 | 58 | Toscana virus | 0.91 | 1.05 | 0.38 |
|  | NC_006318 | 58 | Sandfly fever Naples virus | 0.91 | 1.05 | 0.59 |
|  | JX867536 | 58 | Toscana virus | 0.91 | 1.05 | 0.91 |
|  | HM566182 | 63 | Sand fever Naples-like virus | 0.88 | 0.68 | 0.18 |
|  | EF201832 | 63 | Sandfly fever Naples virus | 0.88 | 0.68 | 0.13 |
|  | HM566170 | 63 | Sandfly fever Naples virus | 0.88 | 0.68 | 1.26 |
|  | EF201828 | 63 | Sandfly fever Naples virus | 0.88 | 0.68 | 1.09 |
|  | EF201824 | 70 | Sandfly fever Sicilian virus | -100 | -100 | null |
|  | JX005842 | 74 | Heartland virus | 0.32 | 0.7 | -1.13 |
|  | JX005843 | 74 | Heartland virus | 0.32 | 0.7 | -1.66 |
|  | JF838326 | 74 | Chize virus | 0.32 | 0.7 | 0.12 |
|  | KF767465 | 79 | Zaliv Terpenia virus | 0.81 | 0.48 | -0.35 |
|  | KF767462 | 79 | Zaliv Terpenia virus | 0.81 | 0.48 | -1.39 |
|  | HM566192 | 79 | Zaliv Terpenia virus | 0.81 | 0.48 | -2.08 |
|  | HM566160 | 79 | EgAN 1825-61 virus | 0.81 | 0.48 | 0.48 |
|  | JF838332 | 79 | Murre virus | 0.81 | 0.48 | -2.56 |
|  | JF838329 | 79 | Grand Arbaud virus | 0.81 | 0.48 | -1.77 |
|  | DQ380152 | 93 | Rift Valley fever virus | 0.88 | 0.93 | 2.28 |
|  | EU312114 | 93 | Rift Valley fever virus | 0.88 | 0.93 | 2.12 |
|  | NC_014395 | 93 | Rift Valley fever virus | 0.88 | 0.93 | 2.33 |
|  | DQ380154 | 93 | Rift Valley fever virus | 0.88 | 0.93 | 2.19 |
|  | DQ380146 | 93 | Rift Valley fever virus | 0.88 | 0.93 | 0.74 |
|  | DQ380161 | 93 | Rift Valley fever virus | 0.88 | 0.93 | 2.02 |
|  | DQ380172 | 93 | Rift Valley fever virus | 0.88 | 0.93 | 2.18 |
|  | EU312109 | 93 | Rift Valley fever virus | 0.88 | 0.93 | 2.17 |
|  | EU312135 | 93 | Rift Valley fever virus | 0.88 | 0.93 | 1.57 |
|  | EU312123 | 93 | Rift Valley fever virus | 0.88 | 0.93 | 1.41 |
|  | DQ380163 | 93 | Rift Valley fever virus | 0.88 | 0.93 | 1.5 |
|  | EU312112 | 93 | Rift Valley fever virus | 0.88 | 0.93 | 2.37 |
|  | EU312124 | 93 | Rift Valley fever virus | 0.88 | 0.93 | 2.41 |
|  | EU312136 | 93 | Rift Valley fever virus | 0.88 | 0.93 | 1.44 |
|  | JQ840746 | 93 | Rift Valley fever virus | 0.88 | 0.93 | 1.51 |
|  | DQ380179 | 93 | Rift Valley fever virus | 0.88 | 0.93 | 1.75 |
|  | EU312122 | 93 | Rift Valley fever virus | 0.88 | 0.93 | 1.15 |
|  | EU312108 | 93 | Rift Valley fever virus | 0.88 | 0.93 | 0.52 |
|  | JF311388 | 93 | Rift Valley fever virus | 0.88 | 0.93 | 1.39 |
|  | JF326200 | 93 | Rift Valley fever virus | 0.88 | 0.93 | 1.75 |
|  | EU574087 | 93 | Rift Valley fever virus | 0.88 | 0.93 | 1.78 |
|  | EU709747 | 93 | Rift Valley fever virus | 0.88 | 0.93 | 1.82 |
|  | EU574066 | 93 | Rift Valley fever virus | 0.88 | 0.93 | 1.65 |
|  | EU312133 | 93 | Rift Valley fever virus | 0.88 | 0.93 | 2.08 |
|  | EU312131 | 93 | Rift Valley fever virus | 0.88 | 0.93 | 1.76 |
|  | JF311391 | 93 | Rift Valley fever virus | 0.88 | 0.93 | 1.63 |
|  | EU312141 | 93 | Rift Valley fever virus | 0.88 | 0.93 | 1.46 |
|  | JQ840745 | 93 | Rift Valley fever virus | 0.88 | 0.93 | 2 |
|  | EU312107 | 93 | Rift Valley fever virus | 0.88 | 0.93 | 0.81 |
|  | HE687307 | 93 | Rift Valley fever virus | 0.88 | 0.93 | 0.66 |
|  | EU312119 | 93 | Rift Valley fever virus | 0.88 | 0.93 | 2.19 |
|  | EU312146 | 93 | Rift Valley fever virus | 0.88 | 0.93 | 1.71 |
|  | EU312128 | 93 | Rift Valley fever virus | 0.88 | 0.93 | 1.63 |
|  | EU312115 | 93 | Rift Valley fever virus | 0.88 | 0.93 | 1.54 |
|  | EU312132 | 93 | Rift Valley fever virus | 0.88 | 0.93 | 2.38 |
|  | EU574086 | 93 | Rift Valley fever virus | 0.88 | 0.93 | 2.1 |
|  | EU312139 | 93 | Rift Valley fever virus | 0.88 | 0.93 | 2.13 |
|  | EU574057 | 93 | Rift Valley fever virus | 0.88 | 0.93 | 1.88 |
|  | JQ820481 | 93 | Rift Valley fever virus | 0.88 | 0.93 | 2.05 |
|  | EU312121 | 93 | Rift Valley fever virus | 0.88 | 0.93 | 1.71 |
|  | EU312116 | 93 | Rift Valley fever virus | 0.88 | 0.93 | 1.69 |
|  | EU312104 | 93 | Rift Valley fever virus | 0.88 | 0.93 | 1.94 |
|  | DQ380157 | 93 | Rift Valley fever virus | 0.88 | 0.93 | 1.67 |
|  | EU312118 | 93 | Rift Valley fever virus | 0.88 | 0.93 | 2.08 |
|  | EU312111 | 93 | Rift Valley fever virus | 0.88 | 0.93 | 1.97 |
|  | DQ380159 | 93 | Rift Valley fever virus | 0.88 | 0.93 | 1.91 |
|  | JQ068142 | 93 | Rift Valley fever virus | 0.88 | 0.93 | 1.76 |
|  | DQ380173 | 93 | Rift Valley fever virus | 0.88 | 0.93 | 1.11 |
|  | EU312117 | 93 | Rift Valley fever virus | 0.88 | 0.93 | 1.4 |
|  | DQ380181 | 93 | Rift Valley fever virus | 0.88 | 0.93 | 0.77 |
|  | DQ380155 | 93 | Rift Valley fever virus | 0.88 | 0.93 | 1.31 |
|  | DQ380156 | 93 | Rift Valley fever virus | 0.88 | 0.93 | -0.59 |
|  | EU312130 | 196 | Rift Valley fever virus | 0.95 | 1.05 | 0.36 |
|  | EU312103 | 196 | Rift Valley fever virus | 0.95 | 1.05 | 0.27 |
|  | JF784388 | 196 | Rift Valley fever virus | 0.95 | 1.05 | 0.32 |
|  | HM566175 | 201 | Odrenisrou virus | -100 | -100 | null |
|  | HM627184 | 202 | Salobo virus | 0.26 | 0.66 | 0.78 |
|  | EF201821 | 202 | Corfou virus | 0.26 | 0.66 | -0.28 |
|  | HM119427 | 205 | Nique virus | 0.4 | 0.64 | -1.96 |
|  | HM119403 | 205 | Alenquer virus | 0.4 | 0.64 | 1.19 |
|  | EF201826 | 208 | Sandfly fever Sicilian virus | -100 | -100 | null |
|  | KF297905 | 212 | Gabek Forest virus | 0.37 | 0 | 0.95 |
|  | NC_023634 | 212 | Arumowot virus | 0.37 | 0 | -0.87 |
|  | HQ661807 | 212 | Armero virus | 0.37 | 0 | 0.44 |
|  | NC_015452 | 212 | Aguacate virus | 0.37 | 0 | -1.24 |
|  | HM566161 | 212 | Ixcanal virus | 0.37 | 0 | -3.78 |
|  | HM566156 | 212 | Durania virus | 0.37 | 0 | 0.36 |
|  | EF201823 | 212 | Sandfly fever Sicilian virus | 0.37 | 0 | -0.62 |
|  | HM566153 | 212 | Phlebovirus CoAr 171616 | 0.37 | 0 | -0.48 |
|  | HM566166 | 212 | Munguba virus | 0.37 | 0 | 1.07 |
|  | EF201825 | 212 | Sandfly fever Sicilian virus | 0.37 | 0 | -0.95 |
|  | NC_015413 | 212 | Sandfly fever Turkey virus | 0.37 | 0 | -1.39 |
|  | EF201822 | 212 | Sandfly fever Sicilian virus | 0.37 | 0 | -0.63 |
|  | EF201827 | 212 | Sandfly fever Sicilian virus | 0.37 | 0 | -1.92 |
|  | EF201817 | 212 | Phlebovirus sp. Be An 356637 | 0.37 | 0 | -0.57 |
|  | EF201819 | 212 | Phlebovirus sp. VP-161A | 0.37 | 0 | 0.73 |
|  | JX961621 | 246 | Bhanja virus | 0.71 | 0.81 | -4.87 |
|  | KC335498 | 246 | Razdan virus | 0.71 | 0.81 | -6.47 |
|  | JQ956381 | 246 | Palma virus | 0.71 | 0.81 | -6.17 |
|  | NC_022632 | 251 | Razdan virus | 0.75 | 0.75 | -7.96 |
|  | JQ956378 | 251 | Bhanja virus | 0.75 | 0.75 | -7.83 |
|  | KC521442 | 251 | Bhanja virus | 0.75 | 0.75 | -5.83 |
|  | JX961624 | 251 | Bhanja virus | 0.75 | 0.75 | -6.63 |
|  | JX961630 | 251 | Palma virus | 0.75 | 0.75 | -6.21 |
|  | JX961627 | 251 | Forecariah virus | 0.75 | 0.75 | -5.45 |
|  | JX961618 | 251 | Bhanja virus | 0.75 | 0.75 | -5.8 |
|  | HM566180 | 264 | Precarious point virus | -100 | -100 | null |
|  | HM566148 | 265 | Chagres virus | 0.34 | 0.46 | -0.97 |
|  | HM119436 | 265 | Oriximina virus | 0.34 | 0.46 | 1.13 |
|  | EF201820 | 268 | Phlebovirus sp. Be Ar 371637 | 0.54 | 0.03 | 1.64 |
|  | EF201830 | 268 | Sandfly fever Naples virus | 0.54 | 0.03 | 0.02 |
|  | HM566178 | 268 | Sand fever Naples-like virus | 0.54 | 0.03 | 0.03 |
|  | KF297914 | 268 | Karimabad virus | 0.54 | 0.03 | -3.41 |
|  | HM119433 | 275 | Turuna virus | 0.39 | 0.24 | -0.1 |
|  | HM119412 | 275 | Echarate virus | 0.39 | 0.24 | -1.82 |
|  | HM119424 | 278 | Morumbi virus | 0.54 | 0 | -1.95 |
|  | HM119421 | 278 | Mucura virus | 0.54 | 0 | -0.8 |
|  | HM466936 | 278 | Jacunda virus | 0.54 | 0 | -2.22 |
|  | HM119430 | 278 | Serra Norte virus | 0.54 | 0 | -0.94 |
|  | HM119418 | 278 | Itaituba virus | 0.54 | 0 | -1.27 |
|  | HM119415 | 278 | Maldonado virus | 0.54 | 0 | -1.87 |
|  | HM119406 | 278 | Ariquemes virus | 0.54 | 0 | -1.7 |
|  | KF297902 | 278 | Gordil virus | 0.54 | 0 | -0.71 |
|  | HM566151 | 278 | Phlebovirus CoAr 170255 | 0.54 | 0 | -0.39 |
|  | EF201839 | 278 | Buenaventura virus | 0.54 | 0 | -0.67 |
|  | GQ847511 | 278 | Sandfly fever Turkey virus | 0.54 | 0 | -2.21 |
|  | HM566168 | 278 | Sandfly fever Naples virus | 0.54 | 0 | -1.12 |
|  | EF201836 | 278 | Phlebovirus sp. Co Ar 171616 | 0.54 | 0 | -1.09 |
|  | EF201838 | 278 | Phlebovirus sp. VP-366G | 0.54 | 0 | 1.4 |
|  | EF201842 | 278 | Phlebovirus sp. VP-334K | 0.54 | 0 | 0.18 |
|  | EF201816 | 307 | Phlebovirus sp. Be An 416992 | 0.35 | 0 | -0.96 |
|  | EF201815 | 307 | Phlebovirus sp. Be An 578142 | 0.35 | 0 | -0.52 |
|  | DQ380150 | 307 | Rift Valley fever virus | 0.35 | 0 | -1.35 |
|  | HM566187 | 307 | Phlebovirus GGP-2011a | 0.35 | 0 | 1.78 |
|  | HM566190 | 307 | Uriurana virus | 0.35 | 0 | 0.15 |
|  | EF201829 | 307 | Sandfly fever Naples virus | 0.35 | 0 | 0.18 |
|  | EF201831 | 318 | Sandfly fever Naples virus | 0.71 | 0.18 | -0.29 |
|  | EF201818 | 318 | Phlebovirus sp. Be An 24262 | 0.71 | 0.18 | 1.03 |
|  | EF201834 | 318 | Punta Toro virus | 0.71 | 0.18 | -1.25 |
|  | EF201843 | 318 | Phlebovirus sp. PAN 479603 | 0.71 | 0.18 | -0.91 |
|  | EF201844 | 318 | Phlebovirus sp. PAN 483391 | 0.71 | 0.18 | -2.69 |
|  | EF201837 | 318 | Phlebovirus sp. GML 902878 | 0.71 | 0.18 | -2.44 |
|  | EF201835 | 318 | Punta Toro virus | 0.71 | 0.18 | -2.48 |
|  | DQ363406 | 318 | Punta Toro virus | 0.71 | 0.18 | -1.22 |
|  | EF201841 | 318 | Phlebovirus sp. Pa Ar 2381 | 0.71 | 0.18 | -2.12 |

Table S1A: Table showing all clusters of all data sets from the RNAclust analysis. Cluster ID corresponds to the node ID in the hirarchical tree.

| Dataset | Cluster ID | Virus list |  |
| --- | --- | --- | --- |
| arenavirus - L | 9 | Whitewater Arroyo virus(2), Tamiami virus(1), Oliveros virus(1), Parana virus(1) |  |
|  | 19 | Pichinde virus(1), Allpahuayo virus(1) |  |
|  | 22 | Pirital virus(2) |  |
|  | 25 | Bear Canyon virus(2) |  |
|  | 28 | Cupixi virus(1), Latino virus(1) |  |
|  | 31 | Chapare virus(1), Sabia virus(2) |  |
|  | 38 | Machupo virus(3), Tacaribe virus(1) |  |
|  | 45 | Junin virus(3) |  |
|  | 50 | Amapari virus(1), Guanarito virus(2) |  |
|  | 55 | Luna virus(3), Mopeia Lassa virus reassortant 29(1), Mopeia virus(1), Lassa virus(4), Mobala virus(1) |  |
|  | 74 | Lujo virus(2) |  |
|  | 77 | Pichinde virus(1), Flexal virus(1) |  |
|  | 92 | Lymphocytic choriomeningitis virus(3) |  |
|  | 97 | Lymphocytic choriomeningitis virus(3) |  |
|  | 102 | Lymphocytic choriomeningitis virus(1) |  |
|  | 103 | Lymphocytic choriomeningitis virus(1) |  |
|  | 104 | Lymphocytic choriomeningitis virus(1) |  |
|  | 105 | Lymphocytic choriomeningitis virus(1) |  |
|  | 106 | Lymphocytic choriomeningitis virus(3) |  |
|  | 111 | Lymphocytic choriomeningitis virus(1) |  |
|  | 112 | Lymphocytic choriomeningitis virus(1) |  |
|  | 113 | Lunk virus NKS-1(1), Lymphocytic choriomeningitis virus(2) |  |
|  | 118 | Lymphocytic choriomeningitis virus(1) |  |
|  | 119 | Ippy virus(1) |  |
|  | 120 | Lymphocytic choriomeningitis virus(1) |  |
| arenavirus - L_complement | 5 | Lymphocytic choriomeningitis virus(1) |  |
|  | 8 | Lymphocytic choriomeningitis virus(3) |  |
|  | 16 | Lymphocytic choriomeningitis virus(3) |  |
|  | 21 | Lymphocytic choriomeningitis virus(4) |  |
|  | 28 | Lymphocytic choriomeningitis virus(3) |  |
|  | 33 | Lymphocytic choriomeningitis virus(1) |  |
|  | 34 | Lymphocytic choriomeningitis virus(1) |  |
|  | 35 | Lunk virus NKS-1(1), Lymphocytic choriomeningitis virus(2) |  |
|  | 40 | Ippy virus(1) |  |
|  | 41 | Lymphocytic choriomeningitis virus(1) |  |
|  | 49 | Tacaribe virus(1), Machupo virus(3) |  |
|  | 56 | Junin virus(3) |  |
|  | 61 | Guanarito virus(2), Amapari virus(1) |  |
|  | 66 | Luna virus(3), Mopeia Lassa virus reassortant 29(1), Lassa virus(4), Mobala virus(1), Mopeia virus(1) |  |
|  | 85 | Cupixi virus(1) |  |
|  | 90 | Pirital virus(2), Oliveros virus(1), Whitewater Arroyo virus(2), Tamiami virus(1) |  |
|  | 101 | Allpahuayo virus(1), Pichinde virus(1), Parana virus(1) |  |
|  | 106 | Bear Canyon virus(2) |  |
|  | 109 | Latino virus(1) |  |
|  | 110 | Chapare virus(1), Sabia virus(2) |  |
|  | 115 | Lujo virus(2) |  |
|  | 118 | Pichinde virus(1), Flexal virus(1) |  |
| arenavirus - S | 3 | Lassa virus(6), Lunk virus NKS-1(1), Mopeia Lassa virus reassortant 29(1), Luna virus(3), Lujo virus(1), Whitewater Arroyo virus(2), Mopeia virus(1), Mobala virus(1), Pirital virus(6), Ippy virus(1), Lymphocytic choriomeningitis virus(23), Parana virus(1), Big brushy tank virus(1), North American arenavirus(2), Pichinde virus(2), Bear Canyon virus(2), Arenavirus H0380005(1), Allpahuayo virus(1) |  |
|  | 119 | Junin virus(10) |  |
|  | 138 | Machupo virus(9) |  |
|  | 155 | Guanarito virus(9), Sabia virus(1), Cupixi virus(1), Chapare virus(1), Amapari virus(1) |  |
|  | 180 | Flexal virus(2), Oliveros virus(1), Morogoro virus(1), Latino virus(1) |  |
|  | 189 | Mopeia virus AN20410(1) |  |
|  | 190 | Tacaribe virus(1) |  |
|  | 191 | Arenavirus AV 96010025(1) |  |
|  | 192 | Tamiami virus(1) |  |
| arenavirus - S_complement | 8 | Junin virus(10) |  |
|  | 27 | Machupo virus(9) |  |
|  | 47 | Guanarito virus(6) |  |
|  | 58 | Guanarito virus(3) |  |
|  | 63 | Amapari virus(1) |  |
|  | 64 | Sabia virus(1), Cupixi virus(1), Chapare virus(1) |  |
|  | 69 | Flexal virus(2), Morogoro virus(1), Oliveros virus(1), Latino virus(1) |  |
|  | 78 | Mopeia virus AN20410(1) |  |
|  | 79 | Tacaribe virus(1) |  |
|  | 83 | Whitewater Arroyo virus(2), Lujo virus(1), Lunk virus NKS-1(1), Pirital virus(1), Arenavirus H0380005(1), Pichinde virus(2), North American arenavirus(2), Big brushy tank virus(1), Parana virus(1) |  |
|  | 107 | Lymphocytic choriomeningitis virus(23) |  |
|  | 152 | Pirital virus(2), Allpahuayo virus(1) |  |
|  | 157 | Ippy virus(1), Mobala virus(1), Lassa virus(6), Luna virus(3), Mopeia Lassa virus reassortant 29(1) |  |
|  | 180 | Pirital virus(3), Mopeia virus(1), Bear Canyon virus(2) |  |
|  | 191 | Arenavirus AV 96010025(1) |  |
|  | 192 | Tamiami virus(1) |  |
| phlebovirus - S | 6 | Corfou virus(1), Rift Valley fever virus(55), Salobo virus(1), Odrenisrou virus(1) |  |
|  | 122 | Chize virus(1) |  |
|  | 125 | Grand Arbaud virus(1) |  |
|  | 127 | Murre virus(1), EgAN 1825-61 virus(1) |  |
|  | 130 | Zaliv Terpenia virus(3) |  |
|  | 135 | Heartland virus(2) |  |
|  | 138 | Nique virus(1), Alenquer virus(1) |  |
|  | 146 | Toscana virus(3) |  |
|  | 151 | Sandfly fever Naples virus(1), Toscana virus(2) |  |
|  | 156 | Sand fever Naples-like virus(1), Sandfly fever Naples virus(3) |  |
|  | 163 | Sandfly fever Sicilian virus(1) |  |
|  | 165 | Phlebovirus JS6(1), SFTS virus HNXY_186(1), Severe fever with thrombocytopenia syndrome virus(8), SFTS virus HNXY_188(1), Huaiyangshan virus(2), SFTS virus HNXY_278(1), SFTS virus HNXY_202(1), SFTS virus HNXY_31(1), SFTS virus HNXY_174(1), SFTS virus HB29(1) |  |
|  | 200 | SFTS virus HB29(1), Huaiyangshan virus(2), Severe fever with thrombocytopenia syndrome virus(1) |  |
|  | 207 | Massilia virus(1) |  |
|  | 208 | Sandfly fever Sicilian virus(1) |  |
|  | 214 | Jacunda virus(1), Mucura virus(1), Morumbi virus(1), Serra Norte virus(1) |  |
|  | 221 | Maldonado virus(1), Ariquemes virus(1), Itaituba virus(1) |  |
|  | 227 | Phlebovirus sp. Co Ar 171616(1), Sandfly fever Naples virus(1) |  |
|  | 230 | Buenaventura virus(1), Gordil virus(1), Phlebovirus CoAr 170255(1) |  |
|  | 235 | Oriximina virus(1), Chagres virus(1), Sandfly fever Turkey virus(1) |  |
|  | 240 | Phlebovirus sp. VP-161A(1), Munguba virus(1), Sandfly fever Sicilian virus(4), Aguacate virus(1), Durania virus(1), Armero virus(1), Gabek Forest virus(1), Arumowot virus(1), Ixcanal virus(1), Phlebovirus sp. Be An 356637(1), Phlebovirus CoAr 171616(1), Sandfly fever Turkey virus(1) |  |
|  | 271 | Turuna virus(1), Echarate virus(1) |  |
|  | 279 | Razdan virus(1), Bhanja virus(1) |  |
|  | 282 | Palma virus(1), Bhanja virus(1) |  |
|  | 285 | Bhanja virus(1) |  |
|  | 286 | Forecariah virus(1), Bhanja virus(1) |  |
|  | 289 | Razdan virus(1), Bhanja virus(1), Palma virus(1) |  |
|  | 294 | Precarious point virus(1) |  |
|  | 296 | Phlebovirus sp. Be Ar 371637(1), Karimabad virus(1) |  |
|  | 299 | Sandfly fever Naples virus(1), Sand fever Naples-like virus(1) |  |
|  | 306 | Rift Valley fever virus(1), Sandfly fever Naples virus(1) |  |
|  | 309 | Phlebovirus sp. Be An 578142(1), Phlebovirus sp. Be An 416992(1) |  |
|  | 312 | Phlebovirus sp. VP-334K(1), Uriurana virus(1), Phlebovirus sp. VP-366G(1), Phlebovirus GGP-2011a(1) |  |
|  | 319 | Punta Toro virus(3), Phlebovirus sp. Pa Ar 2381(1), Phlebovirus sp. PAN 479603(1), Phlebovirus sp. GML 902878(1), Phlebovirus sp. PAN 483391(1) |  |
|  | 332 | Phlebovirus sp. Be An 24262(1), Sandfly fever Naples virus(1) |  |
| phlebovirus - S_complement | 7 | SFTS virus HNXY_174(1), SFTS virus HB29(1), SFTS virus HNXY_202(1), SFTS virus HNXY_31(1), SFTS virus HNXY_278(1), Severe fever with thrombocytopenia syndrome virus(8), SFTS virus HNXY_186(1), Phlebovirus JS6(1), Huaiyangshan virus(2), SFTS virus HNXY_188(1) |  |
|  | 42 | Huaiyangshan virus(2), SFTS virus HB29(1), Severe fever with thrombocytopenia syndrome virus(1) |  |
|  | 49 | Massilia virus(1) |  |
|  | 53 | Toscana virus(3) |  |
|  | 58 | Sandfly fever Naples virus(1), Toscana virus(2) |  |
|  | 63 | Sand fever Naples-like virus(1), Sandfly fever Naples virus(3) |  |
|  | 70 | Sandfly fever Sicilian virus(1) |  |
|  | 74 | Chize virus(1), Heartland virus(2) |  |
|  | 79 | Murre virus(1), Grand Arbaud virus(1), Zaliv Terpenia virus(3), EgAN 1825-61 virus(1) |  |
|  | 93 | Rift Valley fever virus(52) |  |
|  | 196 | Rift Valley fever virus(3) |  |
|  | 201 | Odrenisrou virus(1) |  |
|  | 202 | Salobo virus(1), Corfou virus(1) |  |
|  | 205 | Nique virus(1), Alenquer virus(1) |  |
|  | 208 | Sandfly fever Sicilian virus(1) |  |
|  | 212 | Ixcanal virus(1), Gabek Forest virus(1), Arumowot virus(1), Sandfly fever Turkey virus(1), Phlebovirus CoAr 171616(1), Phlebovirus sp. Be An 356637(1), Aguacate virus(1), Sandfly fever Sicilian virus(4), Phlebovirus sp. VP-161A(1), Munguba virus(1), Armero virus(1), Durania virus(1) |  |
|  | 246 | Razdan virus(1), Bhanja virus(1), Palma virus(1) |  |
|  | 251 | Forecariah virus(1), Razdan virus(1), Bhanja virus(4), Palma virus(1) |  |
|  | 264 | Precarious point virus(1) |  |
|  | 265 | Chagres virus(1), Oriximina virus(1) |  |
|  | 268 | Sandfly fever Naples virus(1), Phlebovirus sp. Be Ar 371637(1), Sand fever Naples-like virus(1), Karimabad virus(1) |  |
|  | 275 | Echarate virus(1), Turuna virus(1) |  |
|  | 278 | Buenaventura virus(1), Morumbi virus(1), Itaituba virus(1), Serra Norte virus(1), Sandfly fever Naples virus(1), Phlebovirus sp. Co Ar 171616(1), Ariquemes virus(1), Mucura virus(1), Maldonado virus(1), Gordil virus(1), Phlebovirus CoAr 170255(1), Phlebovirus sp. VP-366G(1), Sandfly fever Turkey virus(1), Phlebovirus sp. VP-334K(1), Jacunda virus(1) |  |
|  | 307 | Phlebovirus sp. Be An 578142(1), Phlebovirus sp. Be An 416992(1), Uriurana virus(1), Phlebovirus GGP-2011a(1), Sandfly fever Naples virus(1), Rift Valley fever virus(1) |  |
|  | 318 | Phlebovirus sp. Pa Ar 2381(1), Sandfly fever Naples virus(1), Punta Toro virus(3), Phlebovirus sp. PAN 483391(1), Phlebovirus sp. GML 902878(1), Phlebovirus sp. PAN 479603(1), Phlebovirus sp. Be An 24262(1) |  |
| tenuivirus - 2 | 4 | Rice stripe virus(24) |  |
|  | 51 | Rice stripe virus(6) |  |
|  | 62 | Iranian wheat stripe virus(1) |  |
|  | 63 | Maize stripe virus(1) |  |
|  | 64 | Rice grassy stunt virus(3) |  |
| tenuivirus - 2_complement | 0 | Rice stripe virus(30), Rice grassy stunt virus(3), Iranian wheat stripe virus(1), Maize stripe virus(1) |  |
| tenuivirus - 4 | 0 | Iranian wheat stripe virus(1), Rice stripe virus(49), Rice grassy stunt virus(3) |  |
| tenuivirus - 4_complement | 0 | Iranian wheat stripe virus(1), Rice stripe virus(49), Rice grassy stunt virus(3) |  |
| tospovirus - M | 5 | Tomato spotted wilt virus(13) |  |
|  | 31 | Tomato spotted wilt virus(20) |  |
|  | 71 | Tomato spotted wilt virus(10) |  |
|  | 90 | Tomato spotted wilt virus(10) |  |
|  | 109 | Soybean vein necrosis virus(1) |  |
|  | 110 | Calla lily chlorotic spot virus(1), Tomato necrotic ringspot virus(1), Bean necrotic mosaic virus(1) |  |
|  | 115 | Capsicum chlorosis virus(3), Groundnut bud necrosis virus(1), Spider lily necrotic spot virus(1), Tomato yellow ring virus(1), Watermelon silver mottle virus(3), Watermelon bud necrosis virus(2), Melon yellow spot virus(1), Iris yellow spot virus(2), Groundnut ringspot and Tomato chlorotic spot virus reassortant(1), Impatiens necrotic spot virus(4) |  |
|  | 152 | Tomato zonate spot virus(1) |  |
| tospovirus - M_complement | 6 | Tomato spotted wilt virus(20) |  |
|  | 49 | Tomato spotted wilt virus(4) |  |
|  | 56 | Tomato spotted wilt virus(4) |  |
|  | 63 | Tomato spotted wilt virus(1) |  |
|  | 64 | Tomato spotted wilt virus(10) |  |
|  | 83 | Tomato spotted wilt virus(1) |  |
|  | 84 | Tomato spotted wilt virus(13) |  |
|  | 109 | Soybean vein necrosis virus(1) |  |
|  | 110 | Bean necrotic mosaic virus(1) |  |
|  | 111 | Spider lily necrotic spot virus(1), Calla lily chlorotic spot virus(1), Tomato necrotic ringspot virus(1), Tomato yellow ring virus(1), Groundnut ringspot and Tomato chlorotic spot virus reassortant(1) |  |
|  | 122 | Iris yellow spot virus(2), Melon yellow spot virus(1), Capsicum chlorosis virus(3), Watermelon bud necrosis virus(2), Groundnut bud necrosis virus(1) |  |
|  | 140 | Impatiens necrotic spot virus(4) |  |
|  | 147 | Watermelon silver mottle virus(3) |  |
|  | 152 | Tomato zonate spot virus(1) |  |

Table S1B: Table showing all clusters of all data sets from the RNAclust analysis. Cluster ID corresponds to the node ID in the hirarchical tree. Virus list shows the viruses within a cluster, with the number of sequences belonging to this virus in parenthesis.


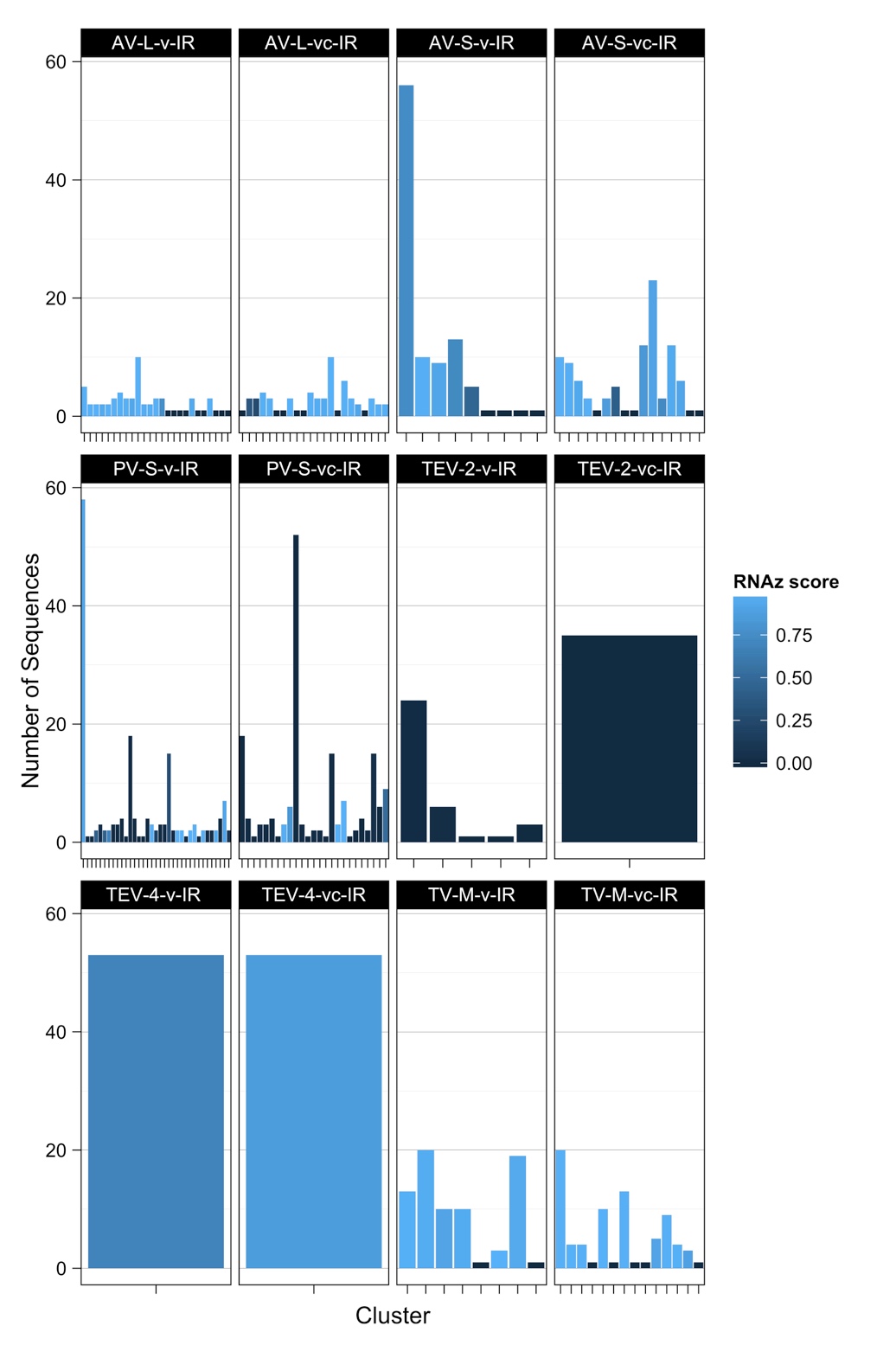


Figure S2: Histograms of cluster sizes for each cluster of each data set, as listed in Table 2. Each bar of a histogram represents one cluster of sequences/structures of a data set, with the bar height showing the amount of sequences in that cluster. Colors represent RNAz functionality prediction of the structures. Dark blue corresponds to not functional, light blue corresponds to functional. The default cut-off in RNAz is 0.5. Clusters of size 1 are not functional by definition, as RNAz relies on MSAs, and thus cannot predict the functionality of a single sequence.

| Data set | ^a^Cluster ID | Number of sequences | ^b^mPID | ^b^SCI | ^c^RNAz prediction | Loop motifs in the consensus structure |
| --- | --- | --- | --- | --- | --- | --- |
| AV-S-v-IGR | 3 | 56 | 0.65 | 0.94 | RNA(0.75) | CCUAAAGG |
|  | 155 | 13 | 0.76 | 0.97 | RNA(0.73) | GAAACC, GCAAUG-C |
|  | 119 | 10 | 0.94 | 1.01 | RNA(0.99) | UCCUGAG, CACUG, CCUGG |
|  | 138 | 9 | 0.94 | 0.99 | RNA(0.93) | CCCAG, CUUUG |
|  | 180 | 5 | 0.68 | 1.02 | OTHER(0.47) | C---GACG, C-UAAG |
|  | 189 | 1 | - | - | - | CUUCG, ACUGUU, CAGAG |
|  | 190 | 1 | - | - | - | CUCCCACG, GUUGCC |
|  | 191 | 1 | - | - | - | GGCCC |
|  | 192 | 1 | - | - | - | CUGUG,AACGCAGU,CAUAUG, CAUCUG |
| AV-S-vc-IGR | 107 | 23 | 0.92 | 0.97 | RNA(0.91) | CUUUCG |
|  | 83 | 12 | 0.55 | 0.93 | RNA(0.91) | CC-AAAGG |
|  | 157 | 12 | 0.87 | 0.92 | RNA(0.96) | CAAUAG |
|  | 8 | 10 | 0.95 | 0.97 | RNA(0.99) | CCAGC, CAGUG |
|  | 27 | 9 | 0.88 | 0.97 | RNA(0.98) | CAAAG, CUGGG |
|  | 47 | 6 | 0.92 | 0.96 | RNA(0.94) | G-CAUUGC, GGUUUC |
|  | 180 | 6 | 0.46 | 0.92 | RNA(0.92) | C-AUUG |
|  | 69 | 5 | 0.46 | 1.01 | OTHER(0.42) | CCAA-G, CCCUC---G |
|  | 58 | 3 | 0.93 | 0.98 | RNA(0.99) | CACUG, GGUUUC, GGGGGC |
|  | 64 | 3 | 0.93 | 1.03 | RNA(0.85) | CAUUUG, CAAUG, GAGG-AU |
|  | 152 | 3 | 0.68 | 1.07 | RNA(0.73) | AA---U, CUUACG, GAGUGC |
|  | 63 | 1 | - | - | - | CCCAGG,GAUGUC,CUGAG |
|  | 78 | 1 | - | - | - | CUCUG,AACAGUCACU,CGAAG |
|  | 79 | 1 | - | - | - | CACAG, GGCAAC, CACACCG, CAAAGCG, CAAAUG |
|  | 191 | 1 | - | - | - | GCCUC |
|  | 192 | 1 | - | - | - | CAGAUG,CAUAUG,CACAUG, CACAG |
| AV-L-v-IGR | 55 | 10 | 0.57 | 0.74 | RNA(0.99) | CCGCCG |
|  | 9 | 5 | 0.65 | 1.00 | RNA(0.99) | CAAAUG, CCCG---GG |
|  | 38 | 4 | 0.74 | 0.85 | RNA(0.99) | GGCCC |
|  | 31 | 3 | 0.80 | 0.91 | RNA(0.99) | CACAG |
|  | 45 | 3 | 0.93 | 0.91 | RNA(0.99) | AGACU, GGUCAGAC, GACCC |
|  | 50 | 3 | 0.69 | 0.81 | RNA(0.93) | CA-GUG, GGAUGC |
|  | 113 | 3 | 0.58 | 0.58 | RNA(0.99) | CCCCCG, CCCAGGCCGAG |
|  | 106 | 3 | 0.74 | 0.68 | RNA(0.96) | CACACCCCG, GGCG---GC, CCCACG |
|  | 92 | 3 | 0.90 | 0.74 | RNA(0.99) | CACACCG, GCGGUC, CGGCCG |
|  | 19 | 2 | 0.43 | 0.97 | RNA(0.99) | CUCC-, CCAGGG |
|  | 22 | 2 | 0.74 | 0.94 | RNA(0.99) | CCAAAG, GGCCCC |
|  | 25 | 2 | 0.69 | 1.01 | RNA(0.96) | CACU-G, GGGCC |
|  | 28 | 2 | 0.29 | 1.04 | RNA(0.99) | CAGCC, CCCCC |
|  | 74 | 2 | 0.54 | 0.74 | RNA(0.98) | CAACCAAG, CCGUCC |
|  | 77 | 2 | 0.19 | 0.76 | RNA(0.99) | CCGCC- |
|  | 102 | 1 | - | - | - | CACCAG,GGAGAAC,CACCACAG,CACCG,GGGCCC, CGGAG |
|  | 103 | 1 | - | - | - | CAACGCAG,CACCCAACG,CACACACACG,CCACACCAG,GGAGGC |
|  | 104 | 1 | - | - | - | GAAGAC, CACAAACAAACACAG, CACAGAG, CACACCCACACCCACACACCCCCAG, GCGGC, CAGCGG |
|  | 105 | 1 | - | - | - | CCACACACCG, CACACCGG, CGCCG, GGGAUC |
|  | 111 | 1 | - | - | - | CAACAG, CAGCAAAAG, GAGCCC, CACACCCG, CGCAGGG, GGAGAC |
|  | 112 | 1 | - | - | - | CACAG, GAGAAGAC, CCACAG, CAAACAGACCCACACACACAG, GCCCCC, CCCGG |
|  | 118 | 1 | - | - | - | CACCCAG, CAAAAG, CAAACACACAGACAGAGAAG, CGACAG,CCCGG |
|  | 119 | 1 | - | - | - | CAAAAACAAAG, GCCAAAAC, CGCAAG, CCCCUG |
|  | 120 | 1 | - | - | - | CAAGACAG, CGAAGCACACCACACACAAG, GACAACCC, AACAACAG, CCCGCG, CAUGCG, GGAGCC |
| AV-L-vc-IGR | 66 | 10 | 0.64 | 0.68 | RNA(0.99) | CGGCGG |
|  | 90 | 6 | 0.77 | 1.00 | RNA(0.99) | CCC---G, CCA-U---G |
|  | 21 | 4 | 0.76 | 0.83 | RNA(0.99) | GACCGC, CGGUGUG, CUUGUCUG, UGUCUGUG |
|  | 49 | 4 | 0.87 | 0.79 | RNA(0.99) | CGUUG |
|  | 8 | 3 | 0.66 | 0.63 | OTHER(0.37) | GCUCCGCC, GCC-C-CC, CG---GGGUGUG |
|  | 16 | 3 | 0.81 | 0.81 | OTHER(0.27) | CGGGG, UGGGUG |
|  | 28 | 3 | 0.61 | 0.83 | RNA(0.99) | GCCGCC, G---GUGC, CUGUGU---UGUG |
|  | 35 | 3 | 0.53 | 0.62 | RNA(0.99) | CGGCCCG, GGGCG-C, CUGGUCCU |
|  | 56 | 3 | 0.95 | 0.88 | RNA(0.98) | CCAUGG |
|  | 61 | 3 | 0.63 | 0.70 | RNA(0.96) | CACAGAG, GGGCC, GAUGAUCUAC |
|  | 101 | 3 | 0.55 | 0.99 | RNA(0.99) | CCC-GG, CGGAG |
|  | 110 | 3 | 0.80 | 0.91 | RNA(0.99) | CUGUG |
|  | 106 | 2 | 0.69 | 1.02 | RNA(0.93) | GGCCC, C-AAUG |
|  | 115 | 2 | 0.55 | 0.79 | RNA(0.99) | UUGGUG |
|  | 118 | 2 | 0.27 | 0.98 | RNA(0.99) | CCACA |
|  | 5 | 1 | - | - | - | CCGAG, CCGGG, GUGGUGUUU, GGGCUGGU |
|  | 33 | 1 | - | - | - | GCCUCC, CCGGUGUG, GUGUGUGUU, UUGUUG |
|  | 34 | 1 | - | - | - | GCCGCC, CCGAGG, UGUUUG, CGCUGG, CUGUGUGUG, GUCUUCUC |
|  | 40 | 1 | - | - | - | GCGGC, UUUGG, CUGUUGUUUG |
|  | 41 | 1 | - | - | - | CGCAUG, CGCGGG, GUGUUGUGUUUGUGUGUGUUUGUC, CUGUGUUG, CUUGUGUG |
|  | 85 | 1 | - | - | - | CACGG, GGACC |
|  | 109 | 1 | - | - | - | GGGCC, CACUG |
| TV-M-v-IGR | 5 | 13 | 0.89 | 0.67 | RNA(0.99) |  |
|  | 31 | 20 | 0.88 | 0.61 | RNA(0.97) |  |
|  | 71 | 10 | 0.96 | 0.82 | RNA(0.92) |  |
|  | 90 | 10 | 0.97 | 0.81 | RNA(0.97) |  |
|  | 109 | 1 | - | - | - |  |
|  | 110 | 3 | 0.48 | 0.17 | RNA(0.99) |  |
|  | 115 | 19 | 0.41 | 0.00 | RNA(0.95) |  |
|  | 152 | 1 | - | - | - |  |
| TV-M-vc-IGR | 6 | 20 | 0.91 | 0.59 | RNA(0.99) |  |
|  | 49 | 4 | 0.99 | 0.91 | RNA(0.99) |  |
|  | 56 | 4 | 0.98 | 0.91 | RNA(0.99) |  |
|  | 63 | 1 | - | - | - |  |
|  | 64 | 10 | 0.96 | 0.92 | RNA(0.99) |  |
|  | 83 | 1 | - | - | - |  |
|  | 84 | 13 | 0.96 | 0.70 | RNA(0.99) |  |
|  | 109 | 1 | - | - | - |  |
|  | 110 | 1 | - | - | - |  |
|  | 111 | 5 | 0.43 | 0.10 | RNA(0.92) |  |
|  | 122 | 9 | 0.35 | 0.00 | RNA(0.99) |  |
|  | 140 | 4 | 0.95 | 0.76 | RNA(0.99) |  |
|  | 147 | 3 | 0.83 | 0.57 | RNA(0.90) |  |
|  | 152 | 1 | - | - | - |  |
| PV-S-v-IGR | 6 | 58 | 0.93 | 0.93 | RNA(0.93) |  |
|  | 165 | 18 | 0.94 | 0.92 | OTHER(0.01) | GAUCAUGAAAUUUAGCC |
|  | 240 | 15 | 0.41 | 0.00 |  |  |
|  | 319 | 7 | 0.64 | 0.46 | RNA(0.93) | AGAUAAGC-U, UGUUGAA, ACAUGUU, UCAUCAA, |
|  | 200 | 4 | 0.95 | 0.75 | OTHER(0.00) | GACAUAUCC |
|  | 312 | 4 | 0.27 | 0.00 | OTHER(0.00) |  |
|  | 130 | 3 | 0.92 | 0.84 | OTHER(0.03) | UUAGAGUCCAAUUA |
|  | 289 | 3 | 0.72 | 0.85 | RNA(0.99) | AUGAUCAUACUU, CAUAUG, GAG-GAC |
|  | 127 | 2 | 0.52 | 0.95 | OTHER(0.43) | CCUAAG, AAAGG |
|  | 135 | 2 | 0.73 | 0.75 | OTHER(0.45) | AUUCAU, CAAUUG |
|  | 138 | 2 | 0.40 | 0.58 | RNA(0.68) | CAAUCAG |
|  | 271 | 2 | 0.37 | 0.35 |  | AAUUUUCUGGUUUUAA, C---UAAUG |
|  | 306 | 2 | 0.24 | 0.77 | OTHER(0.13) | G-ACGCC, CAGUC, GGCUGA |
|  | 309 | 2 | 0.70 | 0.85 | RNA(0.96) | UUACA, AAUAGCU |
|  | 332 | 2 | 0.31 | 0.30 | OTHER(0.11) | ACCCCAUC, CAAA-A, ACAAACACAC, CAAAAAA |
|  | 122 | 1 | - | - | - |  |
|  | 125 | 1 | - | - | - |  |
|  | 146 | 3 | 0.92 | 0.67 | OTHER(0.00) |  |
|  | 151 | 3 | 0.92 | 0.25 | OTHER(0.00) |  |
|  | 156 | 4 | 0.88 | 0.21 | OTHER(0.00) |  |
|  | 163 | 1 | - | - | - |  |
|  | 207 | 1 | - | - | - |  |
|  | 208 | 1 | - | - | - |  |
|  | 214 | 4 | 0.76 | 0.60 | OTHER(0.00) |  |
|  | 221 | 3 | 0.53 | 0.60 | RNA(0.99) |  |
|  | 227 | 2 | 0.75 | 0.86 | OTHER(0.23) |  |
|  | 230 | 3 | 0.50 | 0.32 | OTHER(0.00) |  |
|  | 235 | 3 | 0.23 | 0.08 | OTHER(0.00) |  |
|  | 279 | 2 | 0.58 | 0.89 | RNA(0.99) |  |
|  | 282 | 2 | 0.69 | 0.93 | RNA(0.99) |  |
|  | 285 | 1 | - | - | - |  |
|  | 286 | 2 | 0.72 | 0.90 | RNA(0.99) |  |
|  | 294 | 1 | - | - | - |  |
|  | 296 | 2 | 0.35 | 0.83 | RNA(0.99) |  |
|  | 299 | 2 | 0.74 | 0.96 | OTHER(0.02) |  |
| PV-S-vc-IGR | 93 | 52 | 0.88 | 0.93 | OTHER(0.00) | UGGGGGAAA-G, GGGGC |
|  | 7 | 18 | 0.95 | 0.66 | OTHER(0.00) | GACAUAUCC |
|  | 212 | 15 | 0.37 | 0.00 | OTHER(0.03) |  |
|  | 278 | 15 | 0.54 | 0.00 | OTHER(0.04) |  |
|  | 318 | 9 | 0.71 | 0.18 | RNA(0.52) | GCUUAUC |
|  | 251 | 7 | 0.75 | 0.75 | RNA(0.99) | AUGAUCAGAUUU, CAUAUG |
|  | 307 | 6 | 0.35 | 0.00 | OTHER(0.00) |  |
|  | 42 | 4 | 0.95 | 0.90 | OTHER(0.00) | GAUCAUGAAAUUUAGCC |
|  | 63 | 4 | 0.88 | 0.68 | OTHER(0.00) | CAAAGUGG, UAAUUUGG |
|  | 268 | 4 | 0.54 | 0.03 | OTHER(0.00) |  |
|  | 53 | 3 | 0.92 | 0.98 | OTHER(0.05) | UGGGAUUAGGGGAAGGGG |
|  | 58 | 3 | 0.91 | 1.05 | OTHER(0.00) | UGGGAUGGGG |
|  | 196 | 3 | 0.95 | 1.05 | OTHER(0.02) | UGGGGAAAA-G, GGGGGGUGGGGC |
|  | 246 | 3 | 0.71 | 0.81 | RNA(0.99) | CAUAUG, UUAGCCUA, UUAGCCUG |
|  | 202 | 2 | 0.26 | 0.66 | OTHER(0.00) | AGGGGAGAAGGGGAAAAU, AGGGAC |
|  | 205 | 2 | 0.40 | 0.63 | OTHER(0.01) | AAACAAUCC-UCACACCUCCCACCCCC |
|  | 265 | 2 | 0.34 | 0.46 | OTHER(0.00) | GAAUU-C, AAUGAAA, AAAGUG---G, UAAAUUA |
|  | 275 | 2 | 0.39 | 0.24 | OTHER(0.00) |  |
|  | 49 | 1 | - | - | - |  |
|  | 70 | 1 | - | - | - |  |
|  | 74 | 3 | 0.32 | 0.70 | RNA(0.99) |  |
|  | 79 | 6 | 0.81 | 0.48 | RNA(0.83) |  |
|  | 201 | 1 | - | - | - |  |
|  | 208 | 1 | - | - | - |  |
|  | 264 | 1 | - | - | - |  |
| TEV-2-v-IGR | 4 | 24 | 0.96 | 0.78 | OTHER(0.01) | CUACAGGCACAUACACACACUG, CUAUG, CUACG, CCUAG, AGUAAAU, AGUAUAAU, AAACUACACACUUAAGCU, CUAUUG |
|  | 51 | 6 | 0.93 | 0.77 | OTHER(0.03) | CUAUG, CUACG, CCUAG, GCUUUCU |
|  | 64 | 3 | 0.83 | 0.93 | OTHER(0.00) | AGUAAUUGAU, AUAAGU, GAAAAUAUGCC, UUAAAGUGAAAUA, CUUCGG, GAGAUAUUGUACACC, CCGAAG, UCUCAA, GACUCCC, UUUCAA, UUACAA, AGAUUU |
|  | 62 | 1 | - | - | - |  |
|  | 63 | 1 | - | - | - |  |
| TEV-2-vc-IGR | 0 | 35 | 0.93 | 0.25 | OTHER(0.00) | CGUAG, CUGUAG |
| TEV-4-v-IGR | 0 | 53 | 0.81 | 0.04 | RNA(0.69) | AUACAU-ACUU, CAUCCGGAUG |
| TEV-4-vc-IGR | 0 | 53 | 0.87 | 0.16 | RNA(0.86) | GUGCUACGCAC, UCACGUA, GUAUC, UCGAUAUGGG, CGAUUCG, ACGCAUCGU, UAAAAACAAAGUUAUUAGUAUUUUUG, AACCCAAACU |

Table S 2 The list of clusters for each data set, calculated with RNAclust at k=0.60.
^a^Node ID in the hierarchical tree computed by RNAclust.
^b^mPID and SCI are only available for clusters containing more than one sequence.
^c^Functionality prediction by RNAz with the class probability p-value in parentheses.

| Data set | ^a^Cluster ID | ^b^Structural features | ^c^Representative structure |
| --- | --- | --- | --- |
| AV-S-v-IGR | 3 | stem(18), loop(6) | 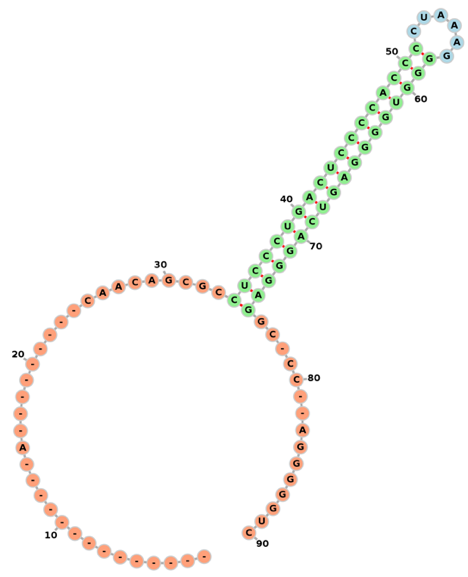structure 1 |
|  | 138 | stem(15,17), loop(3,3) | 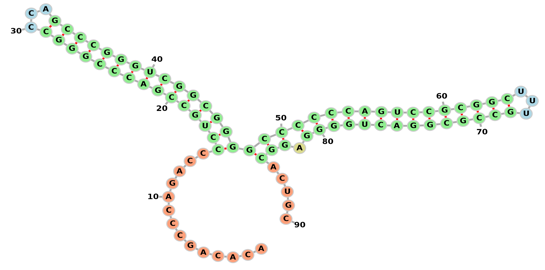 structure 2 |
|  | 155 | stem(15,16), loop(4,6) |
|  | 180 | stem(16,18), loop(7,4) |
|  | 119 | stem(5,13,17), loop(5,3,3) | 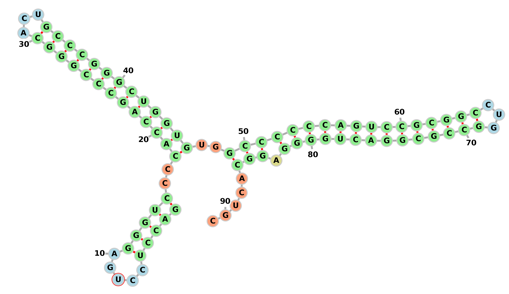 structure 3 |
| AV-S-vc-IGR | 8 | stem(6,12,19), loop(3,3), internal loop(2), bulge(1), mloop(3bp, 2free) | 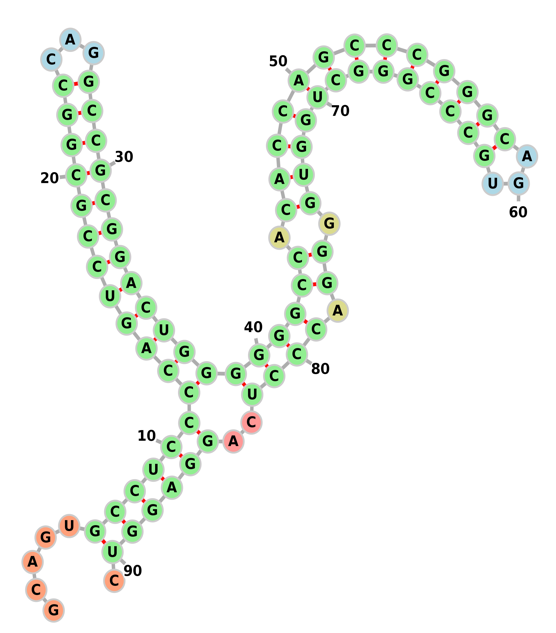 structure 4 |
|  | 27 | stem(5,14,18), loop(3,3), bulge(1), mloop(3bp, 7free) |
|  | 58 | stem(17,14,3), loop(3,4,4), internal loop(2), bulge(1) | 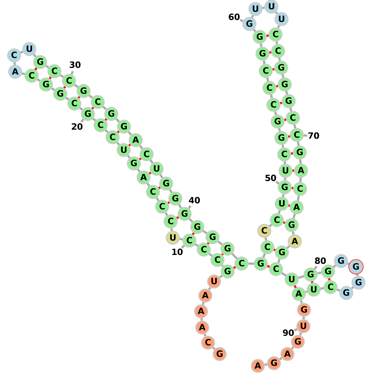 structure 5 |
|  | 64 | stem(17,14,4), loop(4,3,5), internal loop(2), bulge(1) |
|  | 152 | stem(6,17,5), loop(3,4,4) |
|  | 69 | stem(18,16), loop(4,7) | 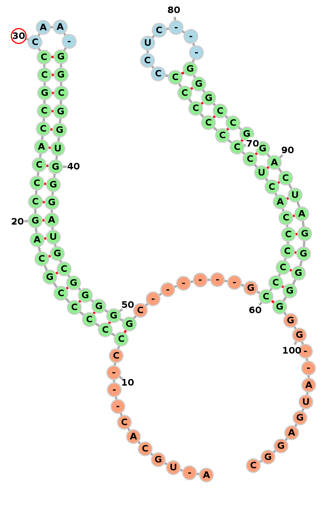 structure 6 |
|  | 47 | stem(16,15), loop(3,4), bulge(1,1) |
|  | 83 | stem(19), loop(6), bulge(1) | 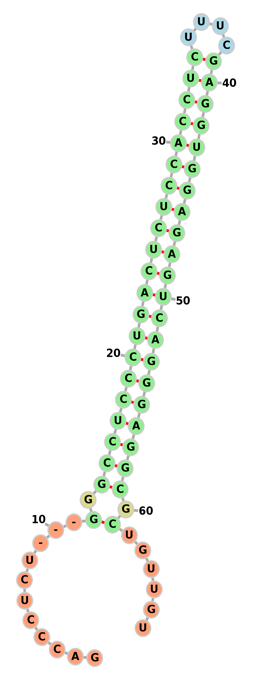 structure 7 |
|  | 107 | stem(22), loop(4), internal loop(2) |
|  | 157 | stem(19), loop(4) |
|  | 180 | stem(19), loop(4) |
| AV-L-v-IGR | 38 | stem(27), loop(3)  internal loop(2,5,6) | 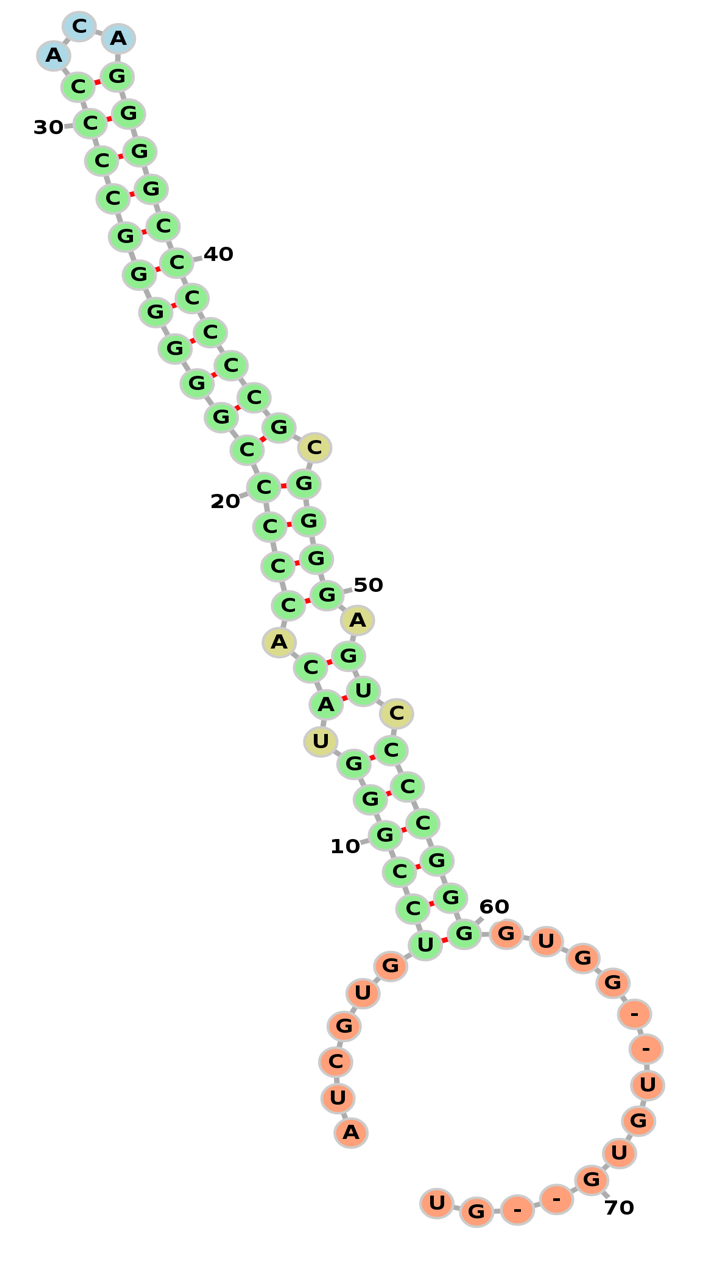 structure 8 |
|  | 31 | stem(24), loop(3)  internal loop(2, 2)  bulge(1) |
|  | 55 | stem(15), loop(4), internal loop(4) |
|  | 77 | stem(16), loop(4) |
|  | 113 | stem(12,7), loop(4,9) | 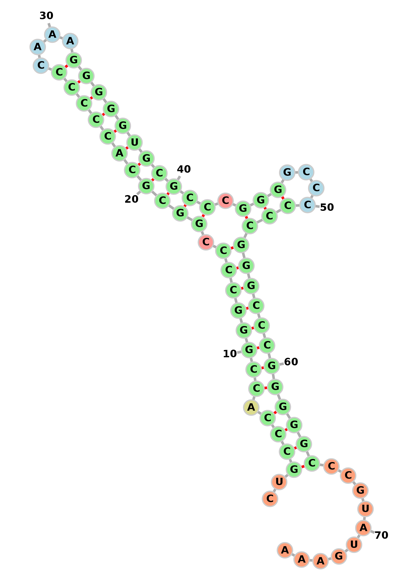 structure 9 |
|  | 50 | stem(16,5,9), loop(4,4)  mloop(3bp, 7free), internal loop(2,9,2) |
|  | 28 | stem(9,9,12), loop(3,3)  mloop(3bp, 5free) |
|  | 9 | stem(7,21), loop(6,4) |
|  | 25 | stem(19,11), loop(4,3)  internal loop(2) |
|  | 19 | stem(3,5,22), loop(4,3)  mloop(3bp, 2free) |
|  | 22 | stem(12,3,11), loop(4,4)  mloop(3bp, 2free) |
|  | 45 | stem(3,4,20), loop(3,6,3)  internal loop(3,4) | 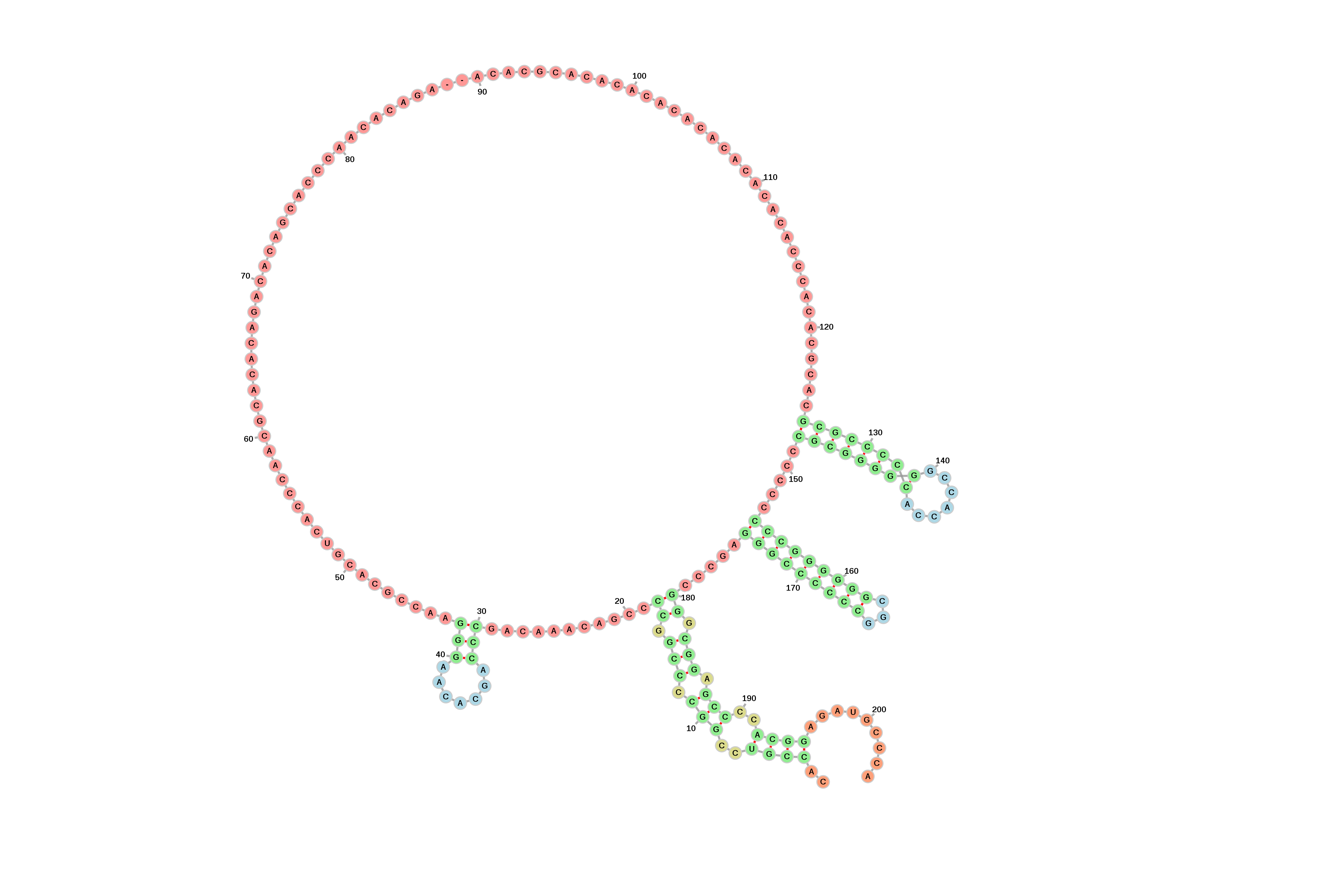 structure 10 |
|  | 74 | stem(6,3,26,6), loop(6,4,3)  mloop(3bp, 21free)  internal loop(2,4,3) |
|  | 97 | stem(12,3,8,9), loop(6,7,3)  mloop(3bp, >20free)  internal loop(4,2) |
|  | 92 | stem(7,2,2,8,10,4), loop(9,8,5,4,4)  mloop(6bp, 95free) | 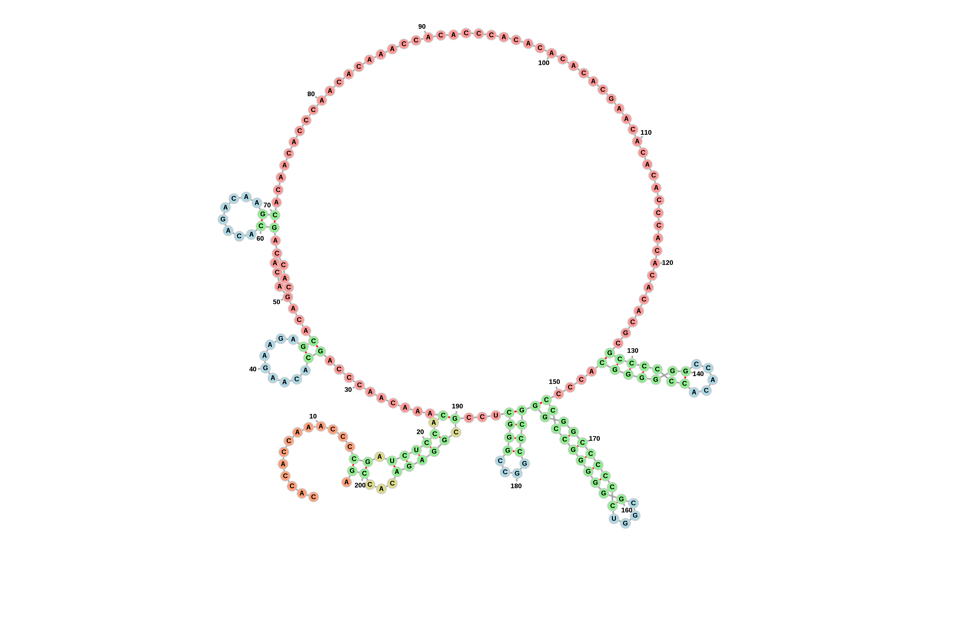 structure 11 |
|  | 106 | stem(3,10,11,6), loop(>10,7,6,4)  mloop(4bp, >20free)  internal loop(6) |
| AV-L-vc-IGR | 8 | stem(6,5,11,11,17,), loop(6,6,9,25), internal loop(4,2,9), bulge(4), mloop(5bp, >20free) | 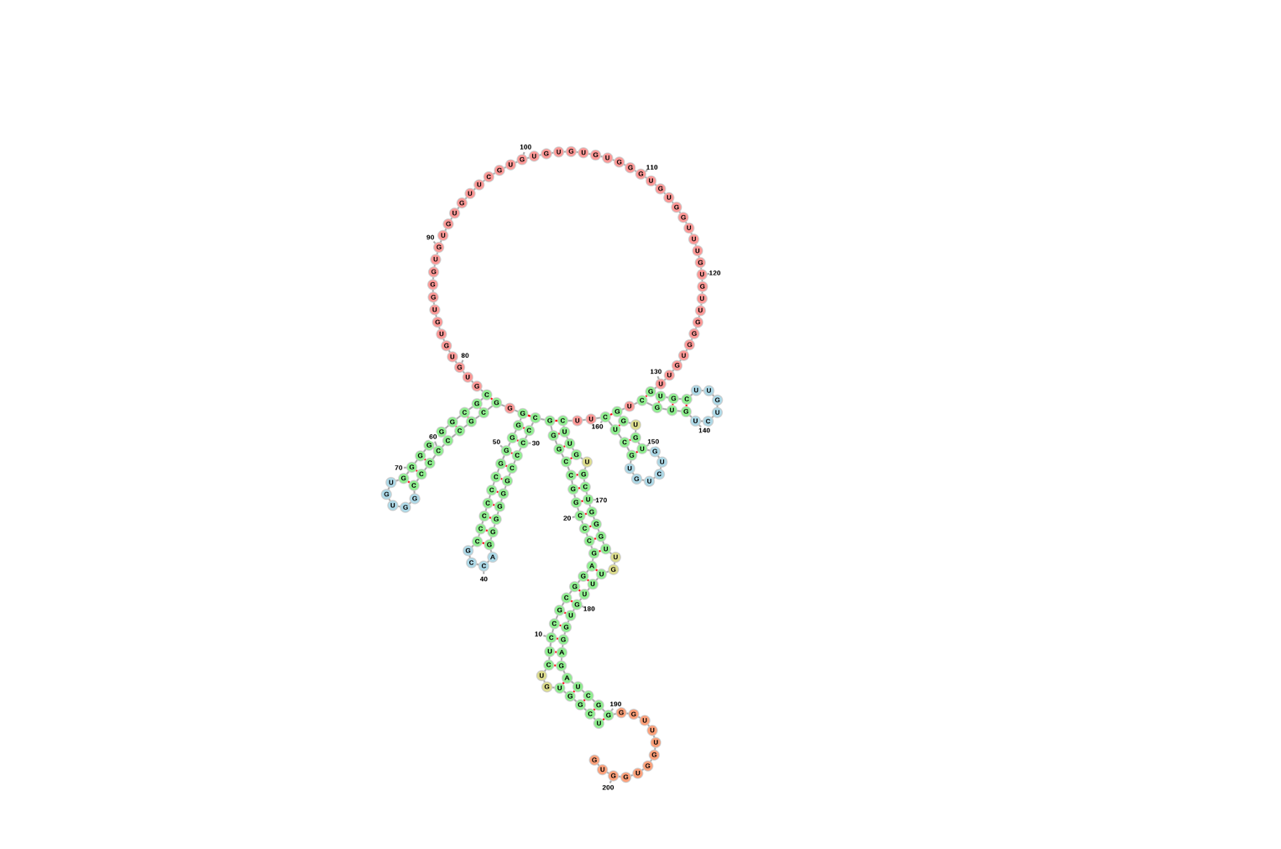 structure 12 |
|  | 21 | stem(25,11,9,4,4), loop(4,5,6,6), bulge(2,2,1,1) |
|  | 28 | stem(18,11,11,16,11), loop(3,6,10,11), internal loop(2,2,3,5,2,2), bulge(1,1), mloop(3bp, 3free) |
|  | 35 | stem(17,13,5), loop(5,5,6), bulge(2,1,4) | 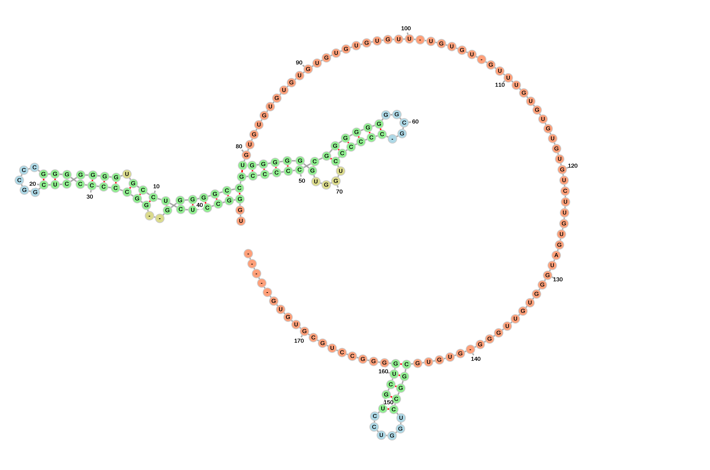 structure 13 |
|  | 16 | stem(17,18,11,16), loop(3,24,4), internal loop(4,2,2,15), bulge(1), mloop(4bp,9free) |
|  | 61 | stem(2,18,5), loop(5,3,6), internal loop(5,4) |
|  | 49 | stem(32), loop(3), internal loop(3,6), bulge(1,2,3) | 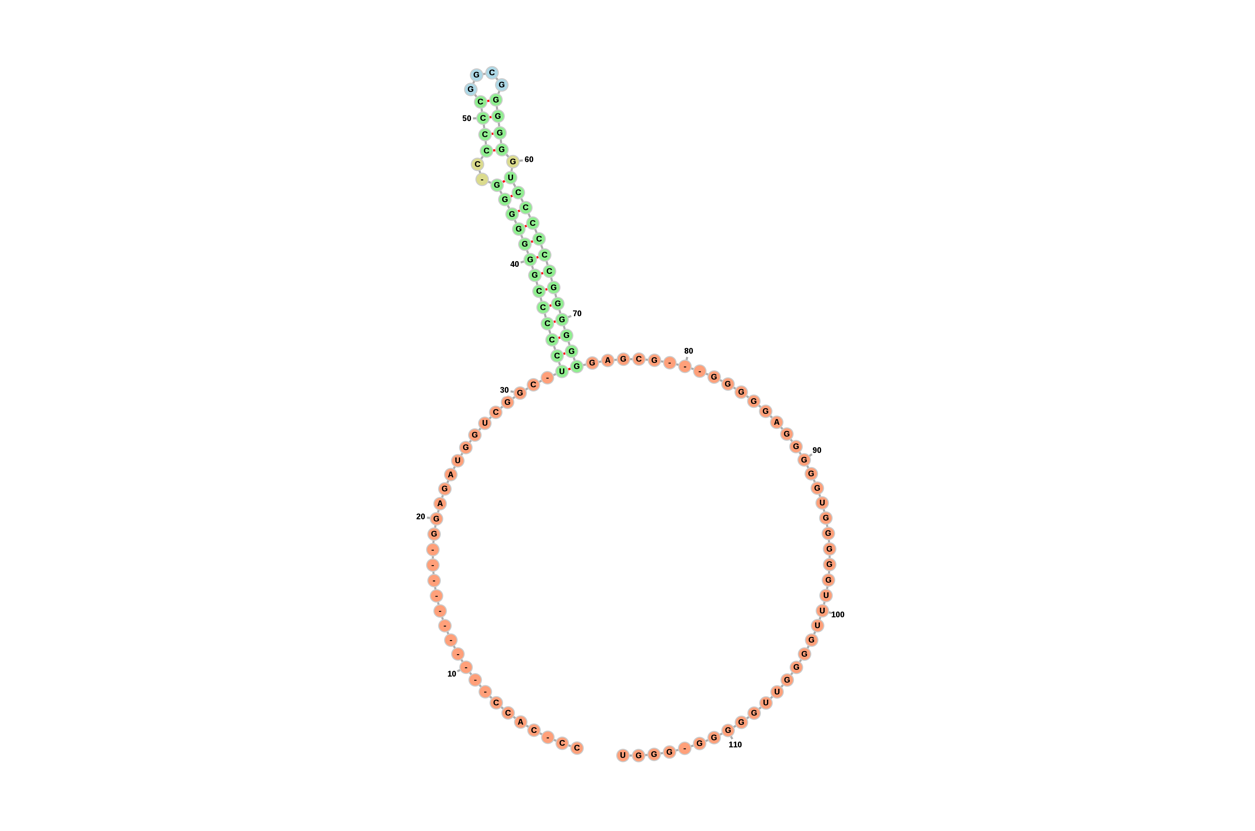 structure 14 |
|  | 56 | stem(26), loop(4), bulge(1,8,9) |
|  | 110 | stem(22), loop(3), internal loop(2,2), bulge(1) |
|  | 66 | stem(17), loop(4), internal loop(3) |
|  | 90 | stem(9,19), loop(4,6), bulge(1), | 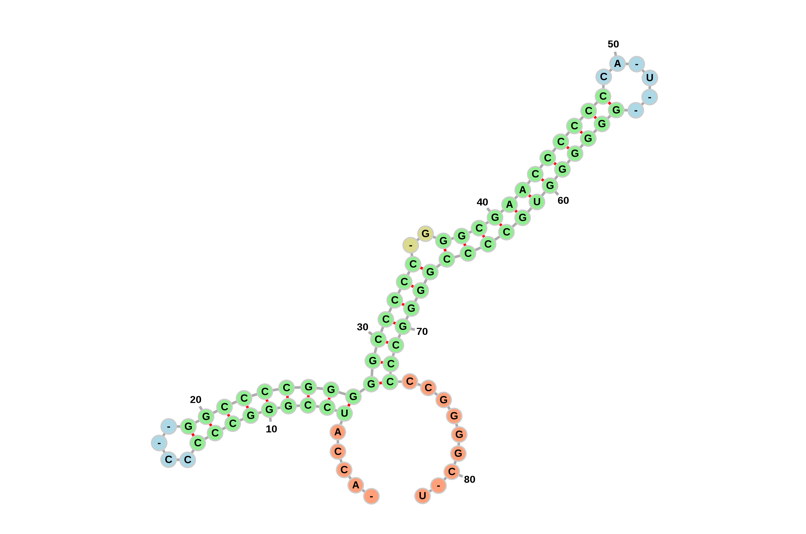 structure 15 |
|  | 101 | stem(7,20), loop(4,3), internal loop(2), bulge(1), |
|  | 106 | stem(11,20), loop(3,4), internal loop(2), |
|  | 115 | stem(10,42), loop(4,4), internal loop(2,2,2,4,2), bulge(1,10,.1,1) |
|  | 118 | stem(10,6), loop(4,3) |
| TV-M-v-IGR | 5 | stem(11,10,11,31,5,4,4), loop(3,3,4,4), mloop(3bp,>20free,2bp, 3free,2bp, 3free,3bp,11free), internal loop(14,7,5) | 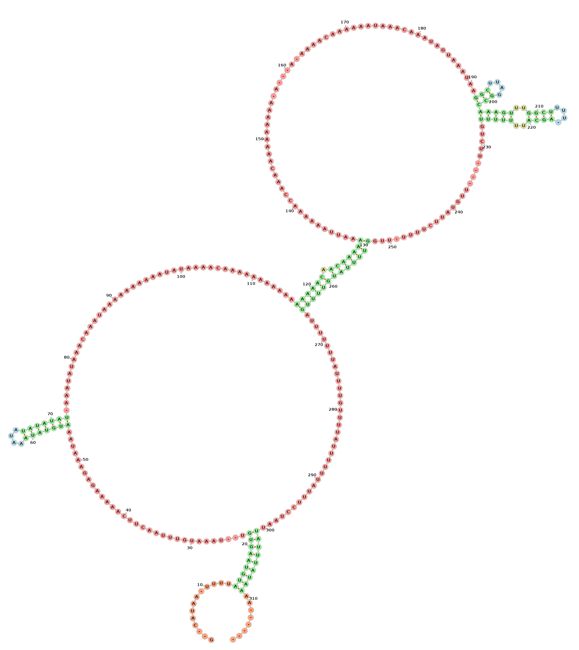 structure 16 |
|  | 31 | stem(4,9,7,11,38,4.4), loop(3,3,4,4), mloop(3bp,14free,3bp>20free,3bp,12free), internal loop(7,5,2,7,5),bulge(3,3) |
|  | 71 | stem(17,5,41,4,4), loop(10,4,4), mloop(3bp, >20free,3bp,>20free), internal loop(6,4,2,5), bulge(1,4,1) |
|  | 90 | stem(4,16,5,44,4,4), loop(5,9,4,4), mloop(3bp,11free,3bp,>20free), internal loop(6,4,11), bulge(1,3,2,1) |
|  | 110 | stem(9,7,13,3,9), loop(4,4,4), mloop(3bp,>20free,3bp,>20free), internal loop(4), bulge(1) |
|  | 115 |  | - |
| TV-M-vc-IGR | 140 | stem(10, 9, 5, 6, 68, 6, 5, 9, 10, 7), loop(7,15, 4, 8, 4, 4, 18, 4), mloop(4bp, 9free, 5bp, >20 free), internal loop(4, 7, 4, 2, 2, 9, 9, 25), bulge(3) | 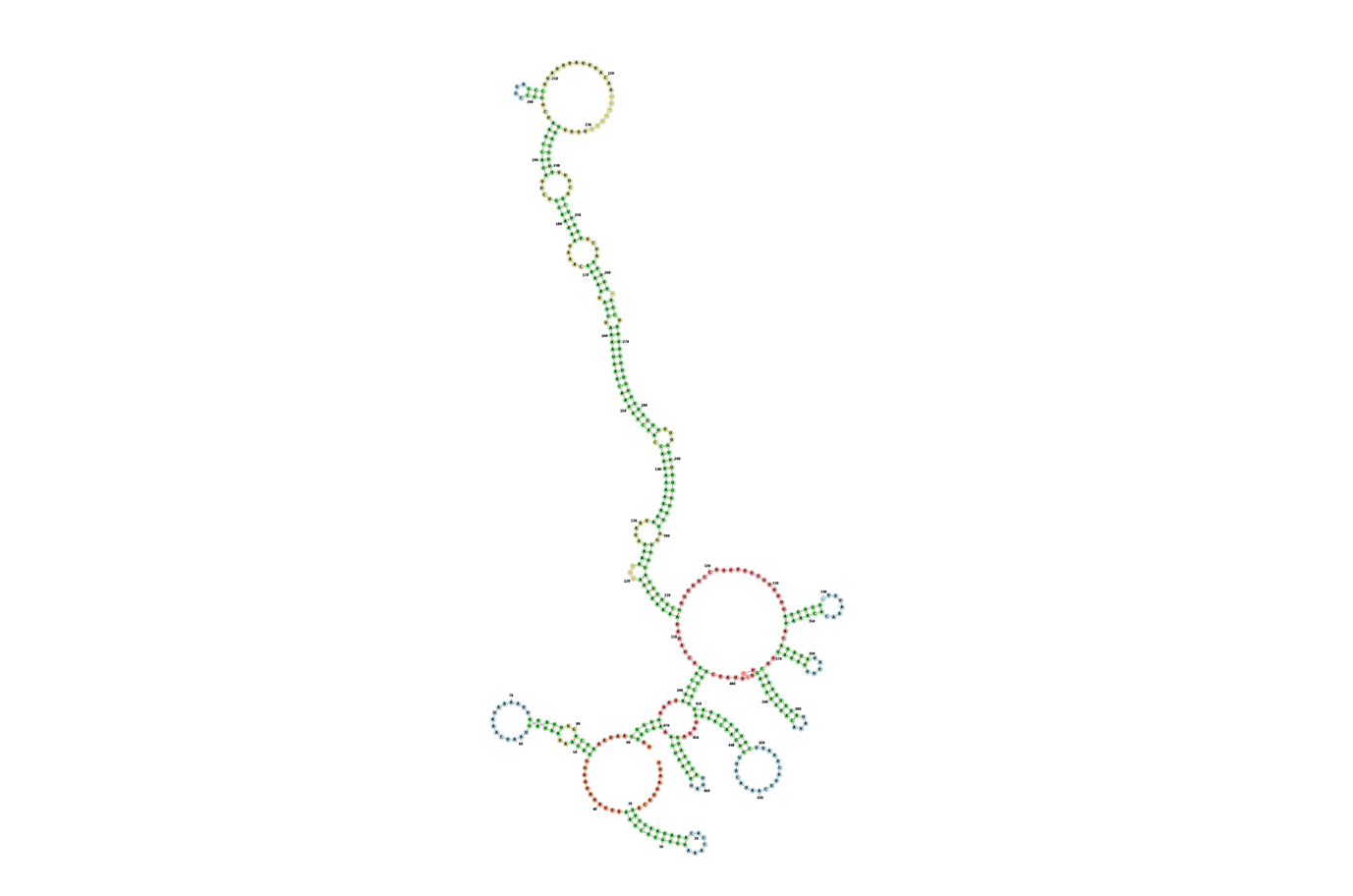 structure 17 |
|  | 84 | stem(20, 9, 8, 46, 3, 10, 8), loop(3, 3, 4, 4, 3), mloop(5bp, >20free, 3bp, 14free), internal loop(6, 6, 9, 3, 8, 8, 2), bulge(1, 1, 2, 3) | 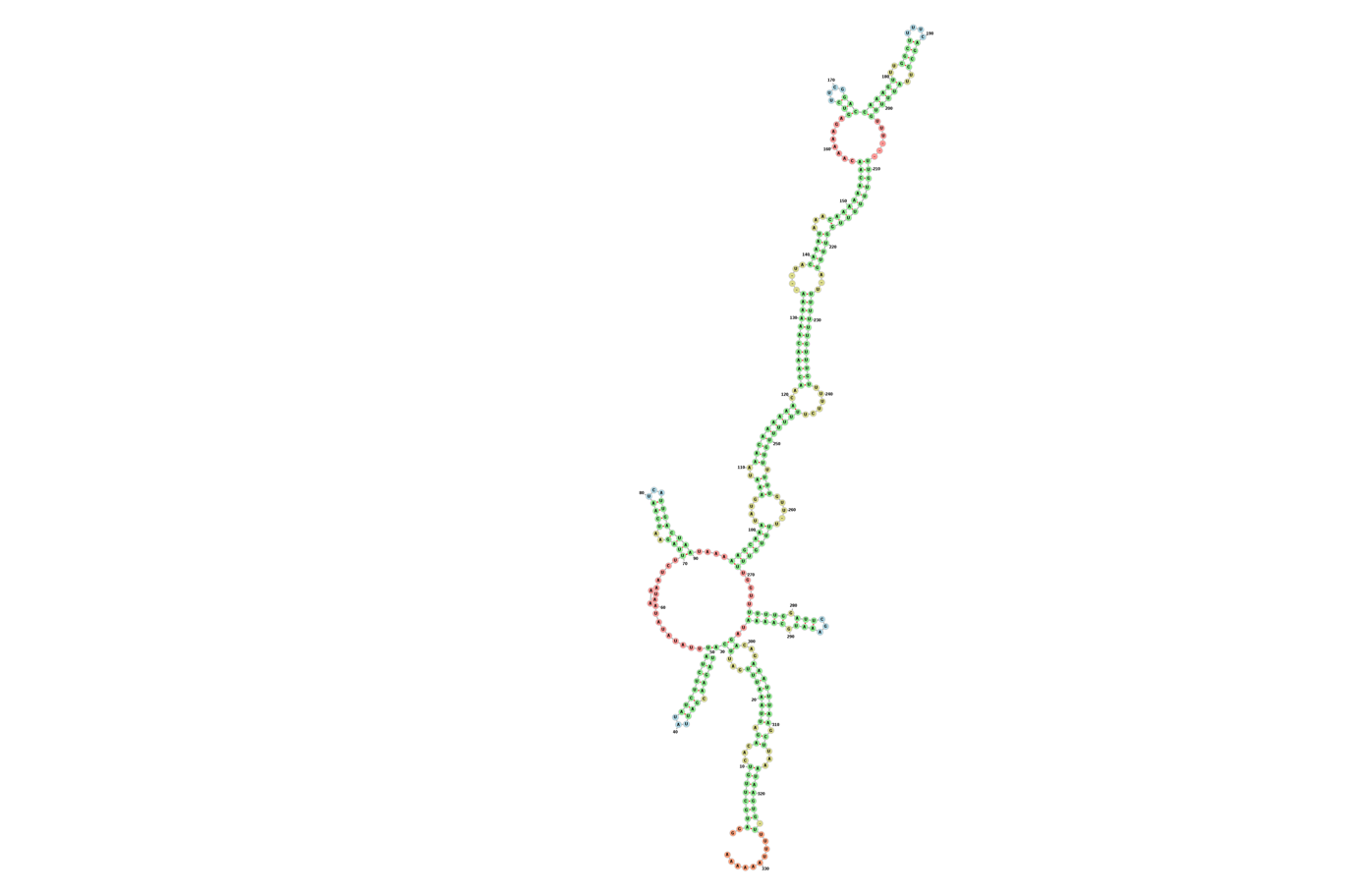 structure 18 |
|  | 147 | stem(17, 12, 42, 12, 8, 5, 12), loop(13, 3, 3, 4, 6), mloop(4bp, >20free, 3bü, >20free), internal loop(5, 8, 6, 2, 6, 2, 6), bulge(2, 4, 7, 1, 2) | 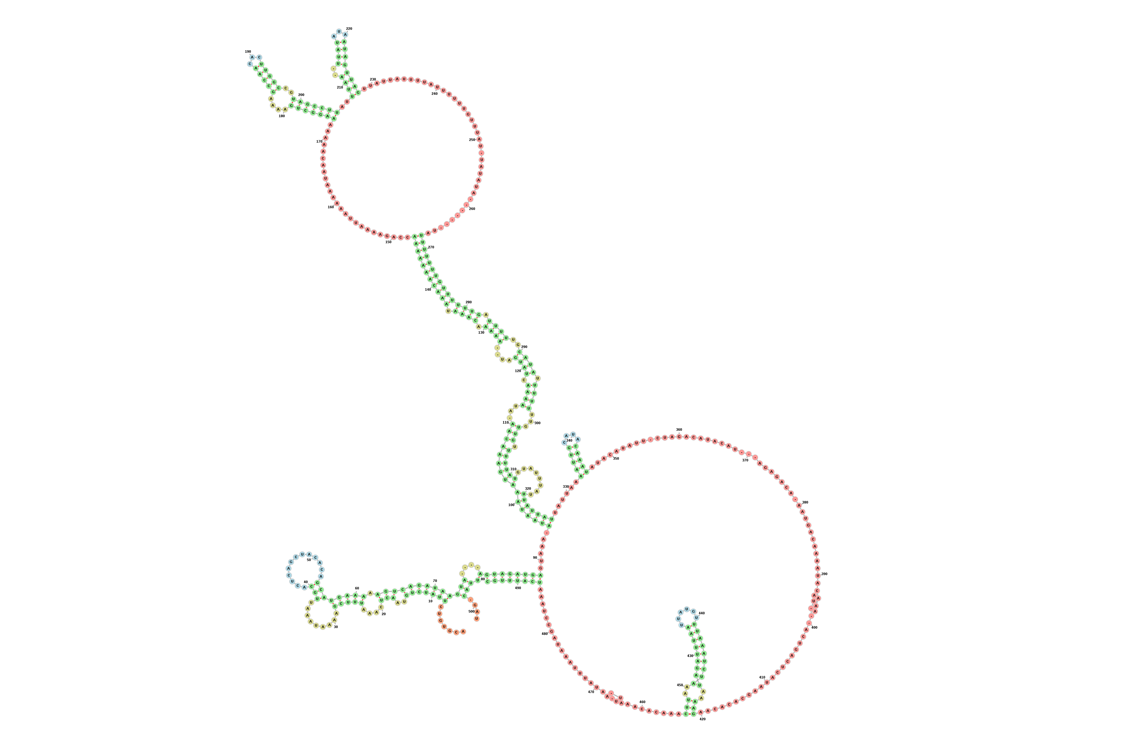 structure 19 |
|  | 49 | stem(14, 12, 6, 54, 17), loop(7, 5, 4, 6), mloop(5bp, 6free), internal loop(5, 2, 2, 2, 2, 5, 8, 3, 4, 2), bulge(1, 2, 1, 3, 4) | 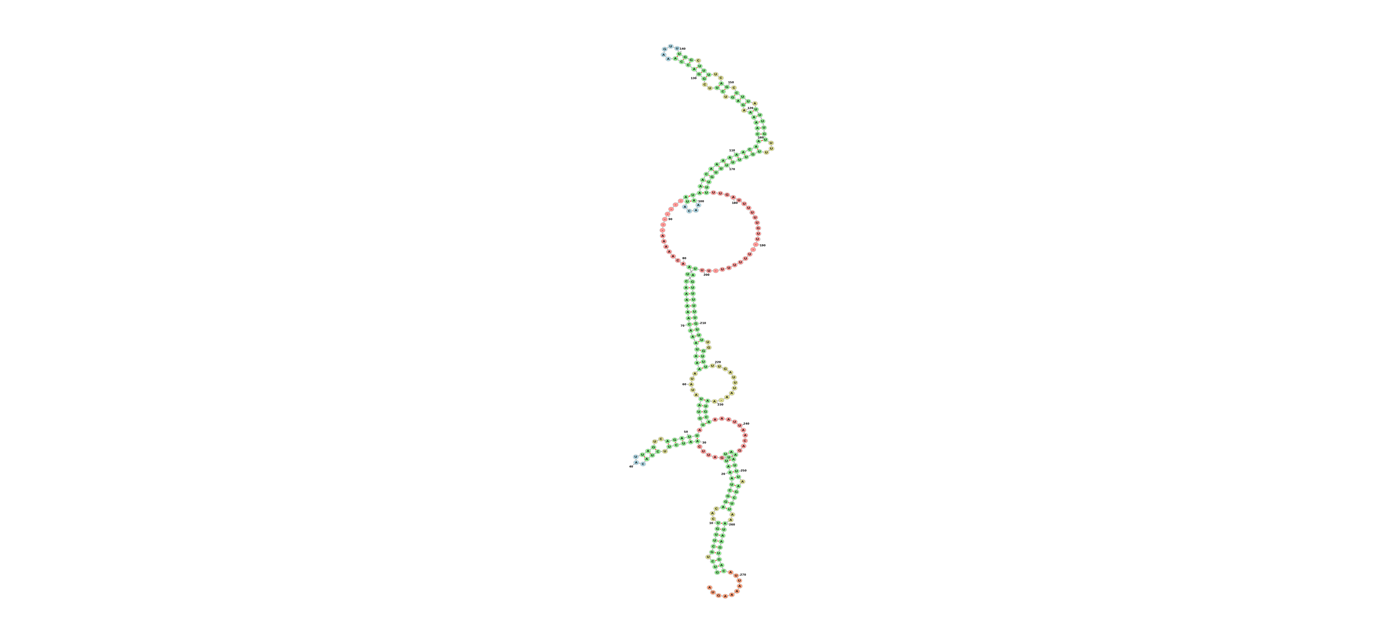 structure 20 |
|  | 56 | stem(14, 12, 55, 11), loop(6, 4, 6), mloop(4bp, >20free), internal loop(5, 2, 2, 5, 10, 7, 4, 2), bulge(1, 2, 1) |
|  | 6 | stem(20, 8, 22, 2, 29), loop(3, 4, 5), mloop(3bp, 16free, 3bp, >20free), internal loop(5, 3, 16, 2, 2, 4), bulge(1, 1, 2, 3, 1) |
|  | 111 | stem(29, 3, 5), loop(8, 6), mloop(3bp, >20free), internal loop(2, 4), bulge(4) | 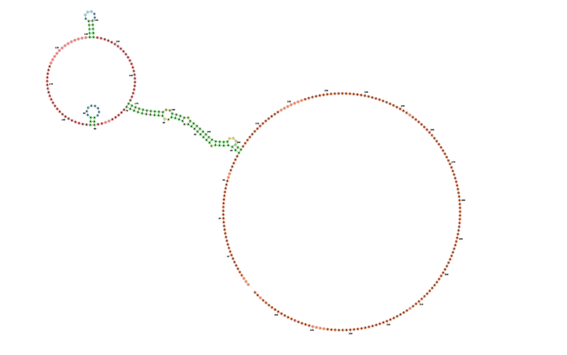 structure 21 |
|  | 64 | stem(100), loop(4), internal loop(5, 10, 4, 4, 6, 15, 6, 2, 8, 3, 4), bulge(4, 12, 1) | 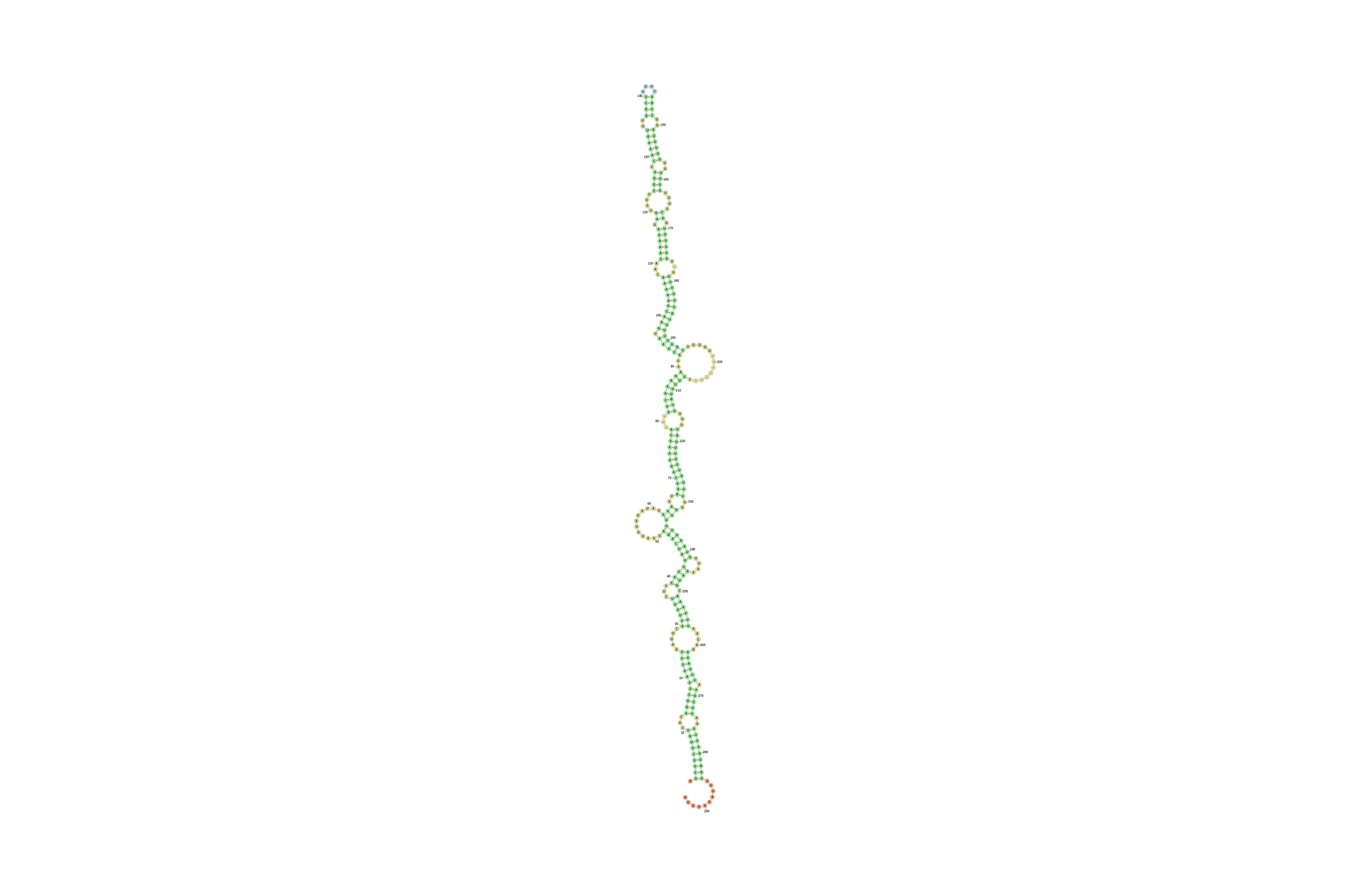 structure 22 |
|  | 122 |  | - |
| PV-S-v-IGR | 6 | stem(8), loop(>20) | 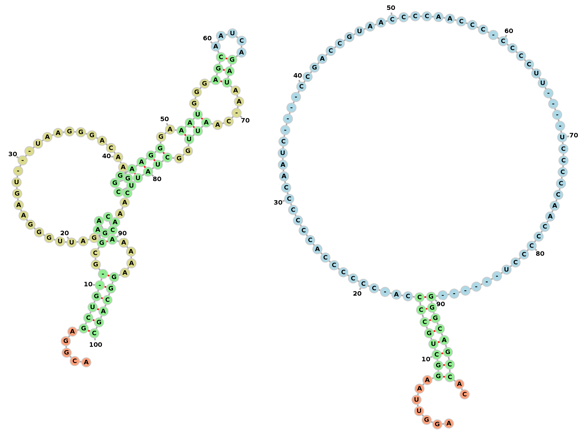 structure 23 |
|  | 296 | stem(7), loop(>20), |
|  | 299 | stem(3,5), loop(5,>20) |
|  | 130 | stem(15), loop(12), internal loop(2,12) |
|  | 135 | stem(15), loop(12), internal loop(2,12) |
|  | 138 | stem(10,13), loop(5), internal loop(6,4,8) |
|  | 146 | stem(7), loop(4), internal loop(10) |
|  | 151 | stem(4), loop(5) |
|  | 156 | stem(4), loop(6), internal loop(2) |
|  | 165 | stem(9), loop(15), internal loop(2) |
|  | 200 | stem(6), loop(7), internal loop(7) |
|  | 282 | stem(8), loop(>20) |
|  | 221 | stem(32,21,10), loop(6,4), internal loop(4,2,2,2,5,4), bulge(1,1,2), mloop(3bp,17free) | 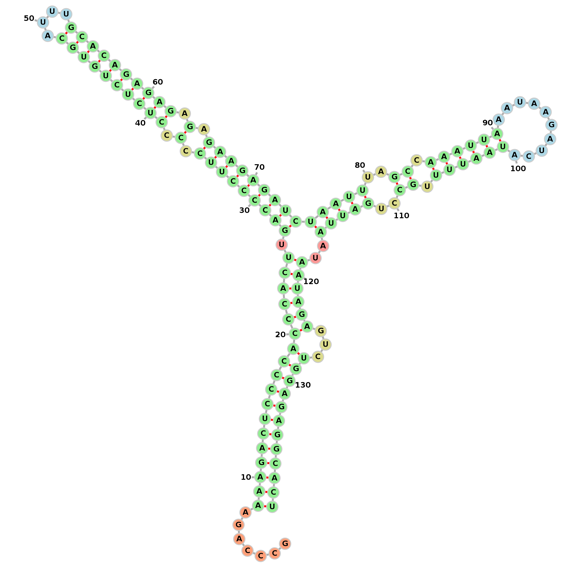 structure 24 |
|  | 235 | stem(5,5,5), loop(7,12), mloop(3bp,>20free) |
|  | 279 | stem(18,20,13), loop(4,10), internal loop(2,2,4,2), bulge(3), mloop(3bp,3free) |
|  | 286 | stem(6,19,17), loop(4,11), internal loop(2,2,2,3), mloop(3bp, 10free), bulge(2) |
|  | 306 | stem(31,7,25,10,6), loop(5,3,4,>20), internal loop(3,3,2,2,2), mloop(3bp,17free), bulge(3,1) |
|  | 309 | stem(2,4,6,13,5), loop(3,3,5,5), internal loop(2,3,2), mloop(4bp,>20free), bulge(1) |
|  | 332 | stem(4,2,2,13,8,5), loop(6,4,8,5,>20), internal loop(4,2,>20,2), mloop(6bp,>20free) |
|  | 289 | stem(9,25,5), loop(10,4,5), internal loop(2,2) | 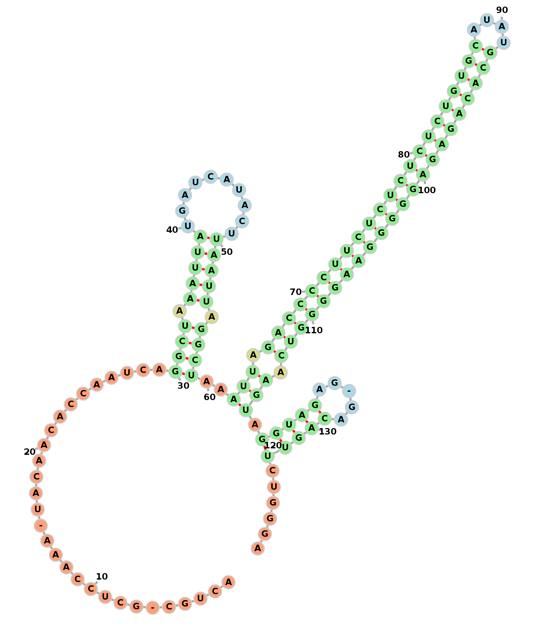 structure 25 |
|  | 319 | stem(5,10,12,7,3,6), loop(5,>20,5,5,5), internal loop(2,2), bulge(1) |
|  | 271 | stem(16,6,6), loop(14,6,4), internal loop(6) |
|  | 227 | stem(11,10,40), loop(7,9,5), internal loop(2,2,2,2,3,11,2), bulge(2,1) |
|  | 127 | stem(4,18), loop(4,3), bulge(3,2) |
|  | 230 | stem(4,10), loop(19,14), internal loop(9) |
|  | 214 | stem(5,21,17,13), loop(63,6), internal loop(2,1,6,2,2,4), bulge(1,1) |
|  | 240 |  | - |
|  | 312 |  | - |
| PV-S-vc-IGR | 265 | stem(), loop(), mloop(), internal loop(), bulge() | 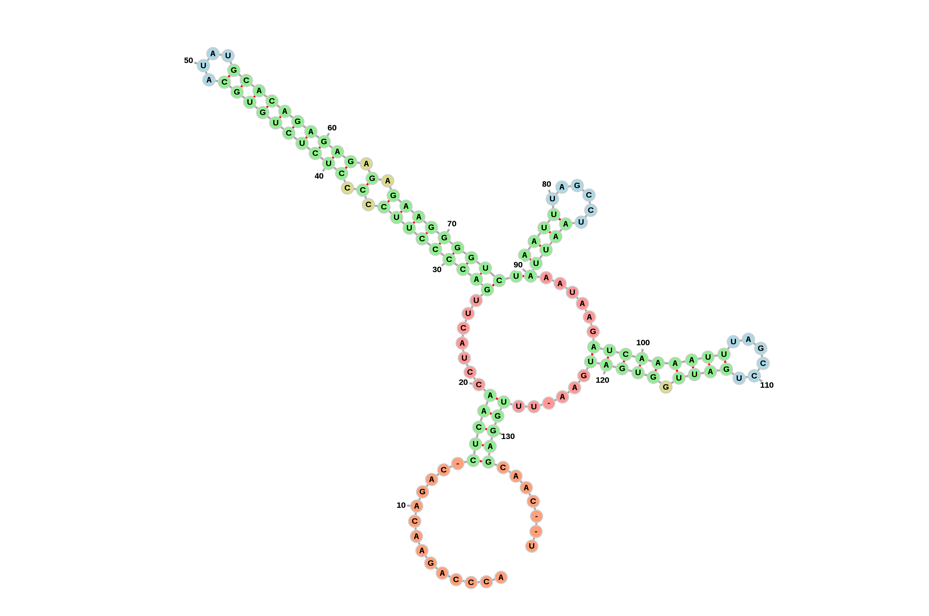 structure 26 |
|  | 246 | stem(5, 20, 5, 9), loop(4, 6, 6), mloop(4bp, 19free), internal loop(2, 2), bulge(1) |
|  | 93 | stem(13, 10), loop(9, 3), internal loop(9, 3, 2), bulge(4) | 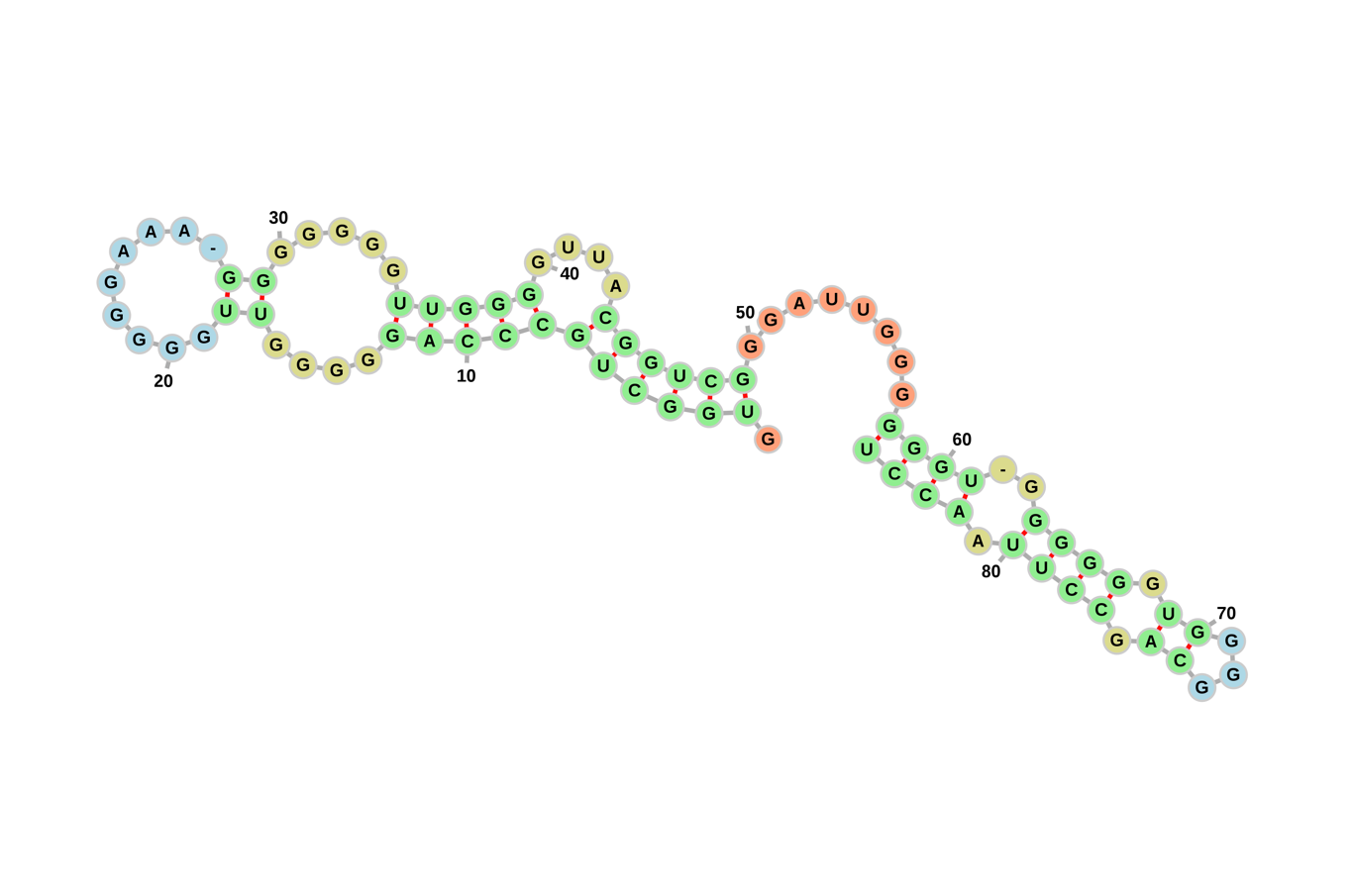 structure 27 |
|  | 251 | stem(7, 25), loop(10, 4), internal loop(6, 2) |
|  | 63 | stem(4, 5), loop(6, 6), internal loop(2) |
|  | 196 | stem(15, 11), loop(9, 10), internal loop(3, 2, 2), bulge(4) |
|  | 202 | stem(13, 9), loop(16, 4), internal loop(2, 4, 6) |
|  | 79 | stem(4, 4), loop(18, 4) |
|  | 318 | stem(23), loop(5), internal loop(6, 6, 2), bulge(1) | 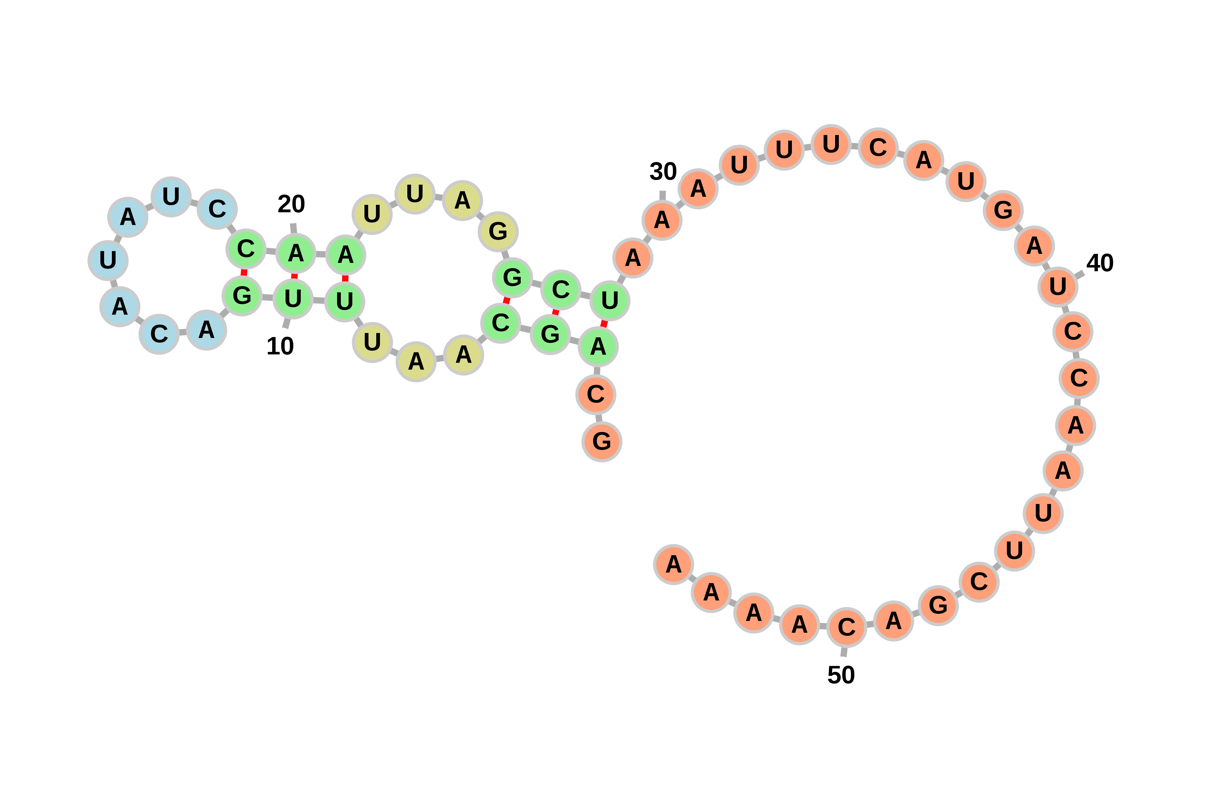 structure 28 |
|  | 53 | stem(17), loop(16), internal loop(3, 6) |
|  | 268 | stem(5), loop(>20) |
|  | 58 | stem(14), loop(8), internal loop(8, 16) |
|  | 7 | stem(6), loop(7), internal loop(7), |
|  | 42 | stem(10), loop(15), internal loop(2) |
|  | 205 | stem(20), loop(>20), internal loop(4, >20), bulge(2) |
|  | 74 | stem(21), loop(7), internal loop(10, 13) |
|  | 275 | stem(18), loop(>20), internal loop(7, 2), bulge(1) |
|  | 278 |  | - |
|  | 307 |  | - |
|  | 212 |  | - |
| TEV-2-v-IGR | 4 | stem(6,4,6,11,7,9,4,21,2), loop(>20,3,3,3,5,6,4), internal loop(2,3,4,4,11,9,15), bulge(1), mloop(7bp,>20free) | 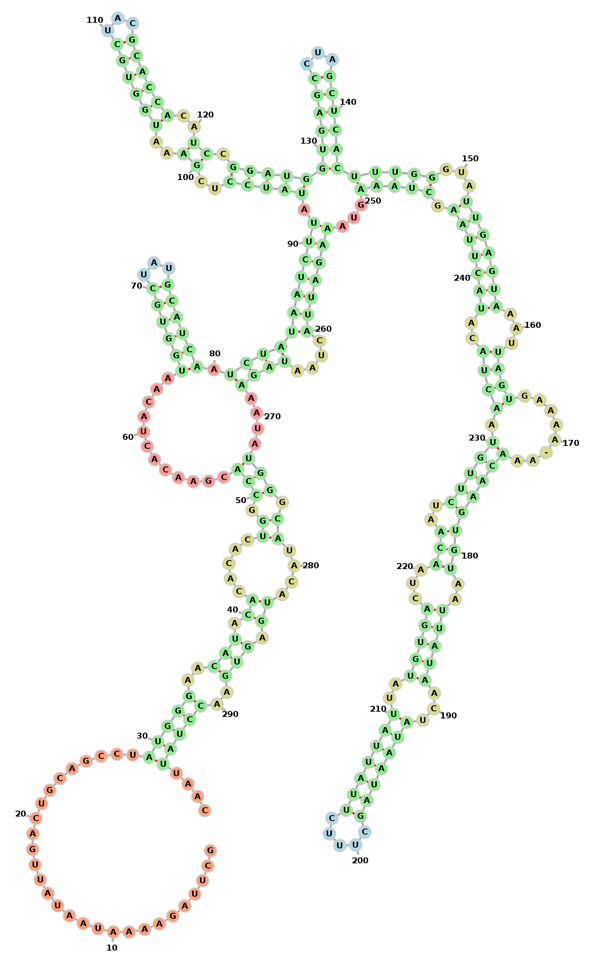 structure 29 |
|  | 51 | stem(15,6,11,13,6,37), loop(3,3,3,4), internal loop(4,2,9,2,3,4,4,5,9,5,5), bulge(2), mloop(3bp,16free,4bp,4free) |
|  | 64 | stem(5,7,6,6,7,38,5,31,4,6,5,6,6,4,3,9,7), loop(8,4,9,11,4,12,4,4,5,4,4,4), internal loop(2,9,10,3,5,4,3,5), bulge(1), mloop(4bp,>20free,3bp,>20free, 5bp,2free,5bp,10free) |
| TEV-2-vc-IGR | 0 | stem(4, 6, 7, 7), loop(4, 3, 4), mloop(3bp, >20free), bulge(6, 5) | 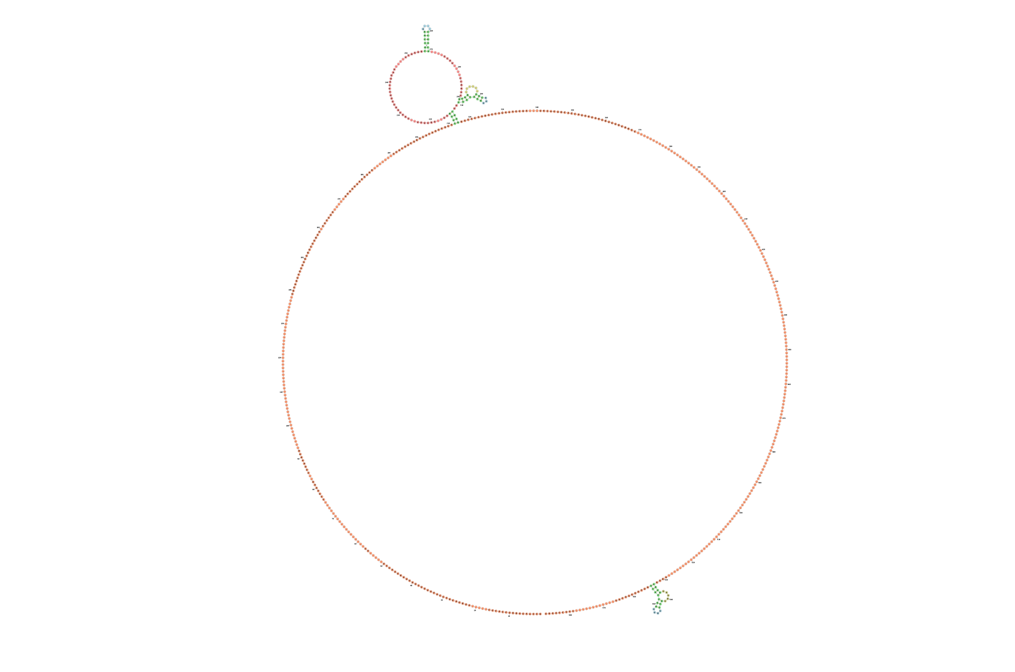 structure 30 |
| TEV-4-v-IGR | 0 | stem(14,5,5,7), loop(>20,9,8), internal loop(2,10,2,14,2), bulge(1,1), mloop(3bp,>20free) | 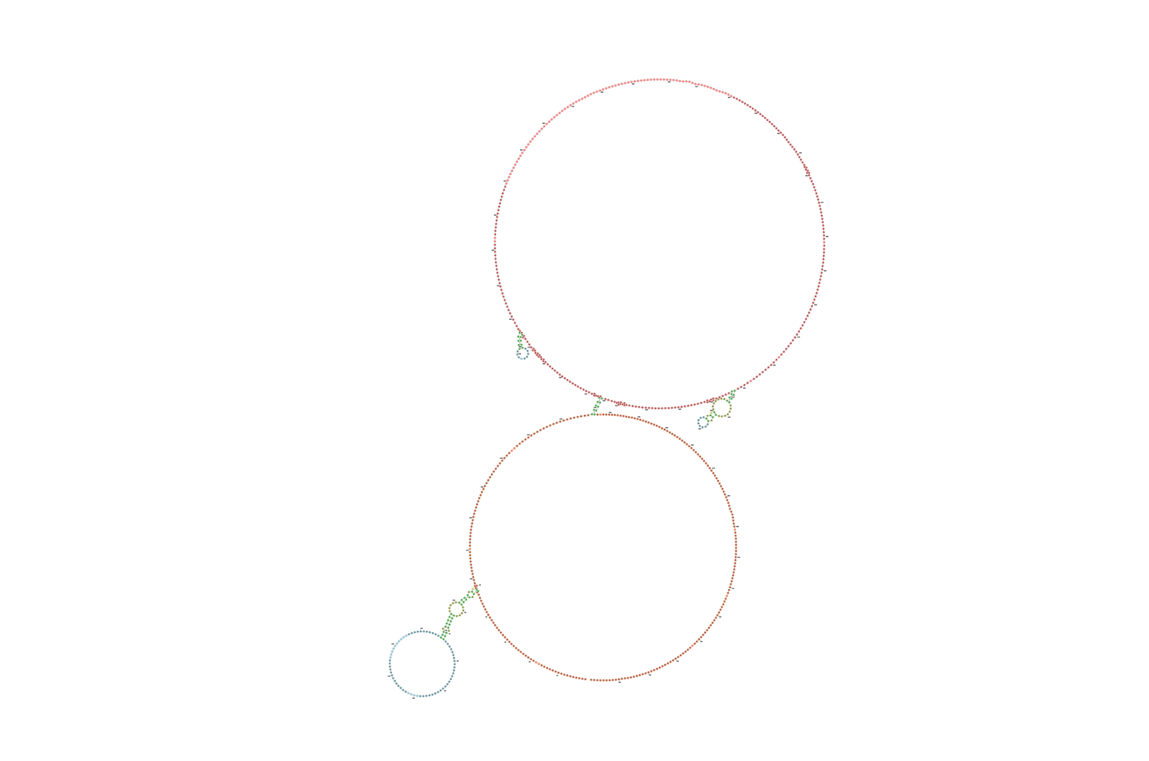 structure 31 |
| TEV-4-vc-IGR | 0 | stem(16, 4, 10, 6, 3, 5, 3, 5, 6, 4, 5), loop(9, 5, 3, 8, 7, 4, >20, >20, 8), mloop(8bp, >20free, 3bp, >20free), internal loop(2, 2, 3), bulge(1, 1) | 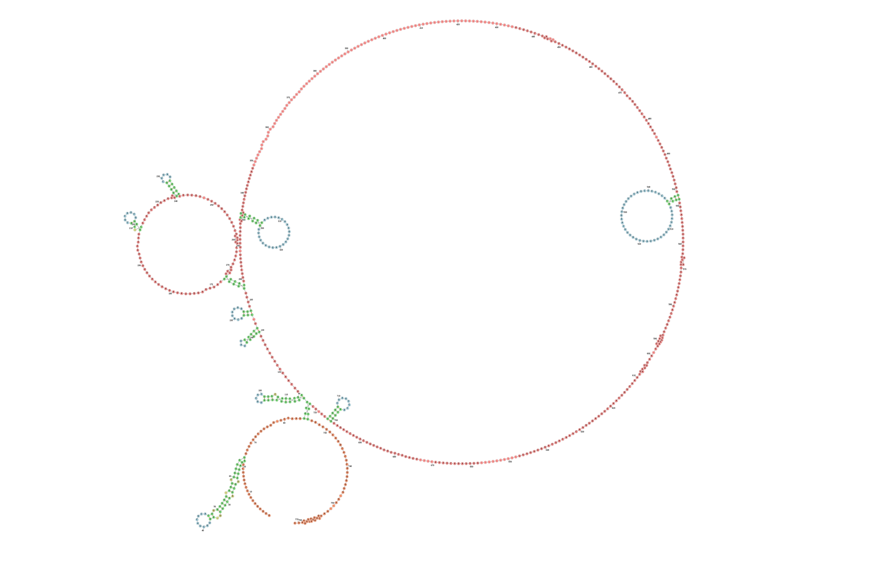 structure 32 |

Table S3: Overview over the consensus structures in each cluster.
^a^Node ID in the hierarchical tree from RNAclust.
^b^List of all structural features (stem. loop. internal loop. bulge. mloop) that are contained in the consensus structure. with the length of each feature in parentheses. Length corresponds to the number of base pairs (bp) for stems. and to the number of unpaired residues for loops. internal loops and bulges. The mloop feature is described by the number of bp and unpaired residues contained in the loop. referred to as bp and free respectively.

^c^Example of a consensus structure. visualized with FORNA.

| Data set | ^a^Cluster ID | ^b^Number of  genera | ^c^Proportion  of data set [%] | ^d^Consensus shape level 3 | ^e^Consensus shape level 5 |
| --- | --- | --- | --- | --- | --- |
| AV-S-v-IGR | 119 | 1 | 10/97 (10%) | [][][[]] | [][][] |
|  | 155 | 5 | 13/97 (13%) | [][[[[]]]] | [][] |
|  | 180 | 4 | 5/97 (5%) | [][] | [][] |
|  | 138 | 1 | 9/97 (9%) | [][[]] | [][] |
|  | 3 | 18 | 56/97 (58%) | [] | [] |
| AV-S-vc-IGR | 152 | 2 | 3/97 (3%) | [][][] | [][][] |
|  | 58 | 1 | 3/97 (3%) | [[]][[]][] | [][][] |
|  | 64 | 3 | 3/97 (3%) | [[]][[]][] | [][][] |
|  | 27 | 1 | 9/97 (9%) | [[][[]]] | [[][]] |
|  | 69 | 4 | 5/97 (5%) | [][] | [][] |
|  | 8 | 1 | 10/97 (10%) | [[][[[]]]] | [[][]] |
|  | 47 | 1 | 6/97 (6%) | [[[]]][] | [][] |
|  | 107 | 1 | 23/97 (24%) | [[]] | [] |
|  | 157 | 5 | 12/97 (12%) | [] | [] |
|  | 83 | 9 | 12/97 (12%) | [[]] | [] |
|  | 180 | 3 | 6/97 (6%) | [] | [] |
| AV-L-v-IGR | 92 | 1 | 3/61 (5%) | [[[[][][][][]]]] | [[][][][][]] |
|  | 106 | 1 | 3/61 (5%) | [][[]][][[[]]] | [][][][] |
|  | 97 | 1 | 3/61 (5%) | [[[[[][][]]]]] | [[][][]] |
|  | 74 | 1 | 2/61 (3%) | [[[][[[]]][[]]]] | [[][][]] |
|  | 45 | 1 | 3/61 (5%) | [][][[[]]] | [][][] |
|  | 22 | 1 | 2/61 (3%) | [[[][]]] | [[][]] |
|  | 19 | 2 | 2/61 (3%) | [[[]][]] | [[][]] |
|  | 25 | 1 | 2/61 (3%) | [[]][] | [][] |
|  | 9 | 4 | 5/61 (8%) | [[]][] | [][] |
|  | 28 | 1 | 2/61 (3%) | [[][]] | [[][]] |
|  | 50 | 2 | 3/61 (5%) | [[[[[[][]]]]]] | [[][]] |
|  | 113 | 2 | 3/61 (5%) | [[]][[[]]] | [][] |
|  | 55 | 5 | 10/61 (16%) | [[]] | [] |
|  | 38 | 2 | 4/61 (7%) | [[[[]]]] | [] |
|  | 31 | 2 | 3/61 (5%) | [[[[]]]] | [] |
|  | 77 | 2 | 2/61 (3%) | [[]] | [] |
| AV-L-vc-IGR | 28 | 1 | 3/61 (5%) | [[[[[[][]]]]]][[[]]][[[]]] | [[][]][][] |
|  | 8 | 1 | 3/61 (5%) | [[][][[]][[[[]]]]] | [[][][][]] |
|  | 21 | 1 | 4/61 (7%) | [[[[[][][][[]]]]]] | [[][][][]] |
|  | 35 | 2 | 3/61 (5%) | [[[]]][[]][] | [][][] |
|  | 16 | 1 | 3/61 (5%) | [[[[[[[]]][][[[]]]]]]] | [[][][]] |
|  | 61 | 2 | 3/61 (5%) | [][[]][[]] | [][][] |
|  | 101 | 3 | 3/61 (5%) | [][[[]]] | [][] |
|  | 90 | 4 | 6/61 (10%) | [][[]] | [][] |
|  | 115 | 1 | 2/61 (3%) | [[[[]]]][[[[[[[]]]]]]] | [][] |
|  | 118 | 2 | 2/61 (3%) | [][] | [][] |
|  | 106 | 1 | 2/61 (3%) | [][[]] | [][] |
|  | 110 | 2 | 3/61 (5%) | [[[[]]]] | [] |
|  | 66 | 5 | 10/61 (16%) | [[]] | [] |
|  | 49 | 2 | 4/61 (7%) | [[[[[[]]]]]] | [] |
|  | 56 | 1 | 3/61 (5%) | [[[[]]]] | [] |
| TV-M-v-IGR | 5 | 1 | 13/77 (17%) | [[[[[[[[[[][]]]]]]][[]]]][]] | [[[[][]][]][]] |
|  | 71 | 1 | 10/77 (13%) | [[[[[[[[[[][]]]]]]][]]]][] | [[[][]][]][] |
|  | 90 | 1 | 10/77 (13%) | [[[[[[[[[[][]]]]]]][]]]][] | [[[][]][]][] |
|  | 110 | 3 | 3/77 (4%) | [[][[[][[]]]]] | [[][[][]]] |
|  | 31 | 1 | 20/77 (26%) | [[[[[[[[[][]]][]]]]]]] | [[[][]][]] |
|  | 115 | 10 | 19/77 (25%) | _ | _ |
| TV-M-vc-IGR | 140 | 1 | 4/77 (5%) | [][[]][[[[[[[[[[[]]]]]]]]][][][]][][]] | [][][[[][][][]][][]] |
|  | 84 | 1 | 13/77 (17%) | [[[[[[[]][[]][[[[[[[][[]]]]]]]][[]]]]]]] | [[][][[][]][]] |
|  | 147 | 1 | 3/77 (4%) | [[[[]]]][[[[[[[[[[[[]][[]]]]]]]]]][][[]]]] | [][[[][]][][]] |
|  | 49 | 1 | 4/77 (5%) | [[[[]][[]][[[[[[[[[[[]]]]]]]]]]][[[]]]]] | [[][][][]] |
|  | 56 | 1 | 4/77 (5%) | [[[[]][[[[[[[[[]]]]]]]]][[]]]] | [[][][]] |
|  | 6 | 1 | 20/77 (26%) | [[[[[[]][[[[][[[[[[]]]]]]]]]]]]] | [[][[][]]] |
|  | 111 | 5 | 5/77 (6%) | [[[[[[][]]]]]] | [[][]] |
|  | 64 | 1 | 10/77 (13%) | [[[[[[[[[[[[[[[[]]]]]]]]]]]]]]]] | [] |
|  | 122 | 5 | 9/77 (12%) | _ | _ |
| PV-S-v-IGR | 332 | 2 | 2/168 (1%) | [[[][][[[]]][[]][]]] | [[][][][][]] |
|  | 319 | 5 | 7/168 (4%) | [[[[]]][][[]][][]] | [[][][][][]] |
|  | 306 | 2 | 2/168 (1%) | [[[[][[[[[]]]]]]]][[]][] | [[][]][][] |
|  | 309 | 2 | 2/168 (1%) | [[][[]][[[]]]][[]] | [[][][]][] |
|  | 271 | 2 | 2/168 (1%) | [[[]]][][] | [][][] |
|  | 227 | 2 | 2/168 (1%) | [[[]]][[]][[[[[[[]]]]]]] | [][][] |
|  | 214 | 4 | 4/168 (2%) | [][[[[[[[]]][[[[]]]]]]]] | [][[][]] |
|  | 289 | 3 | 3/168 (2%) | [[]][[]][] | [][][] |
|  | 235 | 3 | 3/168 (2%) | [[][]] | [[][]] |
|  | 286 | 2 | 2/168 (1%) | [[[[[]]]][[[]]]] | [[][]] |
|  | 221 | 3 | 3/168 (2%) | [[[[[[[[[[]]]][[]]]]]]]] | [[][]] |
|  | 279 | 2 | 2/168 (1%) | [[[[[]]][[[]]]]] | [[][]] |
|  | 135 | 1 | 2/168 (1%) | [[[][]]] | [[][]] |
|  | 230 | 3 | 3/168 (2%) | [][[]] | [][] |
|  | 127 | 2 | 2/168 (1%) | [][[[]]] | [][] |
|  | 299 | 2 | 2/168 (1%) | [][] | [][] |
|  | 282 | 2 | 2/168 (1%) | [[[[[]]]]][[[]]] | [][] |
|  | 200 | 3 | 4/168 (2%) | [[]] | [] |
|  | 130 | 1 | 3/168 (2%) | [[[]]] | [] |
|  | 138 | 2 | 2/168 (1%) | [[[[[]]]]] | [] |
|  | 296 | 2 | 2/168 (1%) | [] | [] |
|  | 146 | 1 | 3/168 (2%) | [[]] | [] |
|  | 156 | 2 | 4/168 (2%) | [[]] | [] |
|  | 6 | 4 | 58/168 (35%) | [] | [] |
|  | 151 | 2 | 3/168 (2%) | [] | [] |
|  | 165 | 10 | 18/168 (11%) | [[]] | [] |
|  | 312 | 4 | 4/168 (2%) | _ | _ |
|  | 240 | 12 | 15/168 (9%) | _ | _ |
| PV-S-vc-IGR | 265 | 2 | 2/168 (1%) | [[[]][][[]][]] | [[][][][]] |
|  | 246 | 3 | 3/168 (2%) | [[[[]]][][[]]] | [[][][]] |
|  | 93 | 1 | 52/168 (31%) | [[[]]][[[]]] | [][] |
|  | 251 | 4 | 7/168 (4%) | [[]][[]] | [][] |
|  | 63 | 2 | 4/168 (2%) | [[]][] | [][] |
|  | 196 | 1 | 3/168 (2%) | [[[[]]]][[]] | [][] |
|  | 202 | 2 | 2/168 (1%) | [[[[]]]][[]] | [][] |
|  | 79 | 4 | 6/168 (4%) | [][] | [][] |
|  | 318 | 7 | 9/168 (5%) | [[[[[]]]]] | [] |
|  | 53 | 1 | 3/168 (2%) | [[[[]]]] | [] |
|  | 268 | 4 | 4/168 (2%) | [] | [] |
|  | 58 | 2 | 3/168 (2%) | [[[]]] | [] |
|  | 7 | 10 | 18/168 (11%) | [[]] | [] |
|  | 42 | 3 | 4/168 (2%) | [[]] | [] |
|  | 205 | 2 | 2/168 (1%) | [[[[]]]] | [] |
|  | 74 | 2 | 3/168 (2%) | [[[]]] | [] |
|  | 275 | 2 | 2/168 (1%) | [[[[]]]] | [] |
|  | 278 | 15 | 15/168 (9%) | _ | _ |
|  | 307 | 6 | 6/168 (4%) | _ | _ |
|  | 212 | 12 | 15/168 (9%) | _ | _ |
| TEV-2-v-IGR | 64 | 1 | 3/35 (9%) | [[[[[]][[[]][[[[[[[[][[[[[[[][][][]]]]]]][][]]]]]]]]][]]][[]]][] | [[[][[][[][[][][][]][][]]][]][]][] |
|  | 4 | 4 | 24/35 (69%) | [[]][[][[[]]][][[]][][[[[]]]]][] | [][[][][][][][]][] |
|  | 51 | 1 | 6/35 (17%) | [[[[[[][[[[[]]][][[[[[[[]]]]]]]]]]]]]] | [[][[][][]]] |
| TEV-2-vc-IGR | 0 | 4 | 35/35 (100%) | [[][[]]][[]] | [[][]][] |
| TEV-4-v-IGR | 0 | 3 | 53/53 (100%) | [[[[[]]]]][[][[[[]]]]] | [][[][]] |
| TEV-4-vc-IGR | 0 | 3 | 53/53 (100%) | [[[[]]]][[[]][][][[[]][]][][][]] | [][[][][][[][]][][][]] |

Table S4: Consensus shapes of all clusters obtained by RNAclust analysis.
^a^Node ID in the hirarchical tree from the RNAclust analysis.
^b^Number of different genera contained in the cluster.
^c^Number of sequences contained in the cluster, compared to the total data set size. The rounded percentage value is given in parentheses.
^d^Level 3 shape representation of the consensus structure.
^e^Level 5 shape representation of the clusters consensus structure.
